# Optimizing a long‐term monitoring strategy to track emerging cyanobacterial blooms in a Great Lakes estuary

**DOI:** 10.1111/jpy.70215

**Published:** 2026-08-07

**Authors:** Peter A. Birschbach, Euan D. Reavie, Christopher T. Filstrup, Hannah Nicklay

**Affiliations:** ^1^ University of Minnesota, Natural Resources Research Institute Hermantown Minnesota USA; ^2^ Division of Extension, Lake Superior National Estuarine Research Reserve University of Wisconsin – Madison Superior Wisconsin USA

**Keywords:** biological monitoring optimization, climate change, cyanobacteria, drought, environmental predictors, model, nitrogen, nutrients, phytoplankton community ecology, spatiotemporal redundancy

## Abstract

Cyanobacterial harmful algal blooms (cHABs) are increasingly frequent and severe globally. Recently, the St. Louis River Estuary (SLRE) has experienced numerous cHABs, but the locations, timing, and environmental conditions associated with them are unknown. We first investigated environmental predictors of cyanobacteria through a multifaceted approach, highlighting key relationships to understand past cHABs, and then performed a detailed spatiotemporal redundancy analysis of sampling data to optimize site selection and sampling frequency for future monitoring. We employed an oversampling approach over 2 years at eight spatially heterogeneous stations. Random forest analysis paired with constrained ordination indicated that warm, late‐summer temperatures were an important predictor of cyanobacterial abundance (biovolume), and drought‐mediated stoichiometric nitrogen limitation may have facilitated dominance by diazotrophic cyanobacteria in 2023. Temporal redundancy analysis revealed no ideal sampling frequency, but late summer, weekly sampling, and as‐needed bloom response collections were deemed critical for tracking cHABs. A 30‐ to 40‐day lag effect between water temperature and cyanobacterial abundance suggested temperature as an early warning predictor of summer bloom intensity. Water quality was more spatially heterogeneous than phytoplankton communities, informing future monitoring site selection designed to comprehensively capture water quality variability and minimize phytoplankton community redundancy while prioritizing sites with past blooms. Our findings inform an optimized monitoring program for the SLRE and demonstrate a feasible monitoring optimization approach that could be developed and applied elsewhere to track risks from cHABs and other stressors.

**Abbreviations**
ANC: acid‐neutralizing capacity
AOC: area of concern
CCA: canonical correspondence analysis
cHAB: cyanobacterial harmful algal bloom
Chl *a*
: chlorophyll *a*
DCA: detrended correspondence analysis
DIN: dissolved inorganic nitrogen
DO: dissolved oxygen
DOC: dissolved organic carbon
EPA: U.S. Environmental Protection Agency
LSNERR: Lake Superior National Estuarine Research Reserve
NMDS: non‐metric multidimensional scaling
NRRI: Natural Resources Research Institute
OOB: out‐of‐bag
PAR_kd_
: photosynthetically active radiation extinction coefficient
PERMANOVA: permutational multivariate analysis of variance
RFA: random forest analysis
SLRE: St. Louis River Estuary
spcond: specific conductivity
SRP: soluble reactive phosphorus
SWMP: system‐wide monitoring program
TN: total nitrogen
TN, TP: total nitrogen to total phosphorus molar ratio
TOC: total organic carbon
TP: total phosphorus
TSS: total suspended solids
VIF: variance inflation factor
VSS: volatile suspended solids

## INTRODUCTION

The frequency and intensity of cyanobacterial harmful algal blooms (cHABs) appear to be globally increasing (Huisman et al., 2018). Cyanobacterial harmful algal blooms are marked by short‐term increases in cyanobacterial abundance and dominance, often leading to odorous and visible surface accumulations (scums; aggregates of planktonic and/or benthic cyanobacteria) and/or macroscopic colonies suspended in the upper water column. Many bloom‐forming taxa can produce cyanotoxins (Carmichael et al., 2001), a potent array of neurotoxins and hepatotoxins including microcystin, saxitoxin, cylindrospermopsin, and anatoxin. Reported human and animal cyanotoxin poisonings have increased 10‐fold over the past century (Wood, 2016). There is no known effective method for removal of cyanotoxins from drinking water at‐scale, so cHABs occurring near municipal intakes have caused unpredictable and immediate crisis‐level incidents, such as in the 2014 Toledo, Ohio, drinking water crisis in western Lake Erie (Bullerjahn et al., 2016). Additionally, decomposition of cHAB biomass can contribute to hypoxia or anoxia, leading to internal nutrient loading, thus initiating a positive feedback loop fueling future cHABs and further aquatic habitat degradation (Jane et al., 2021; Nürnberg, 1984).

Because cHABs pose a serious public health risk and disrupt aquatic ecosystems, research and monitoring efforts have focused on identifying underlying environmental drivers that facilitate their occurrence. Perhaps the most classically studied and commonly observed driver associated with algal proliferation and cHABs is cultural eutrophication, which occurs when the anthropogenic loading of nutrients such as phosphorus results in phytoplankton increases and shifts toward cyanobacterial dominance (Schindler, 1974; Vollenweider, 1968). Through management intervention, cultural eutrophication has stabilized or reversed in many lakes in recent decades, but it remains prevalent in estuarine and coastal waters, in part owing to high human population densities (Cloern & Jassby, 2025).

Not all systems with increasing cHAB occurrences suffer from eutrophication (Reinl et al., 2021). Recent studies have highlighted different drivers, often climate related (O'Neil et al., 2012; Paerl & Paul, 2012). Temperature is a key factor, with the warming waters of global lakes (Woolway et al., 2020) and estuaries (Prum et al., 2024) favoring bloom‐forming cyanobacteria with higher thermal optima than competing phytoplankters (Carey et al., 2012; Paerl & Huisman, 2009) and sometimes increasing cHAB toxicity (Davis et al., 2009; Walls et al., 2018). Temperature effects are highly variable globally (O'Reilly et al., 2015) but are pronounced in northern temperate regions. Lake Superior, for example, despite its cold oligotrophic nature, has experienced recent bloom intensification (Reinl et al., 2023; Sheik et al., 2022; Sterner et al., 2020) and is the fastest warming lake on the planet (Austin & Colman, 2008). In other rapidly warming regions, like subarctic Canada and Sweden, temperature has interplayed with certain climate‐mediated nutrients to promote cyanobacteria through complex mechanisms (Freeman et al., 2020; Przytulska et al., 2017).

The frequency and intensity of droughts and floods have been on variable climate‐driven trajectories globally (Hirabayashi et al., 2008), and aquatic systems are impacted when both occur. Floods deliver large pulses of allochthonous nutrients and sediment, whereas droughts may increase water clarity by settling suspended solids and reducing allochthonous dissolved organic carbon compounds. Droughts can facilitate nutrient depletion as they are consumed by biota and not replenished, which can create conditions favorable for diazotrophic cyanobacteria species through their unique abilities to access dinitrogen (Fogg, 1971). These complex interactions between nutrients, temperature, and multiple climate‐mediated mechanisms complicate large‐scale dataset interpretations and make system‐specific analysis pivotal (Richardson et al., 2018; Wilkinson et al., 2022). Without understanding system‐specific dynamics, managers cannot design monitoring programs or interventions that effectively reduce bloom risk.

**FIGURE 1:**
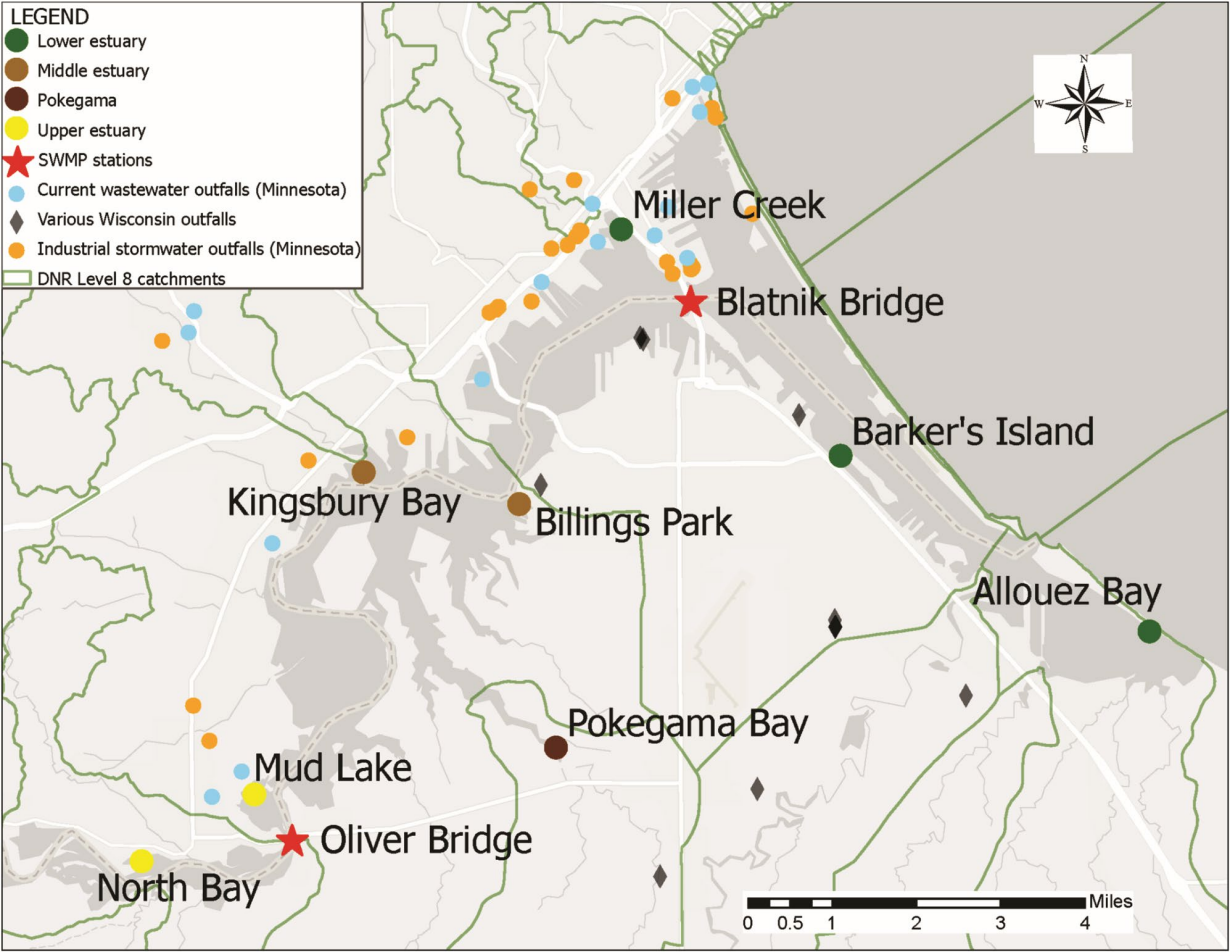
St. Louis River Estuary (SLRE) map showing the eight project sample locations (large circles), additional long‐term monitoring stations from the System‐wide Monitoring Program (stars), catchment regions and documented industrial and wastewater outfalls. Outfall data were sourced from the Wisconsin DNR's Surface Water Data Viewer (https://dnr.wisconsin.gov/topic/SurfaceWater/swdv) and the Minnesota Natural Resource Atlas (https://mnatlas.org).

The St. Louis River Estuary (SLRE), located at the western tip of Lake Superior (Figure 1), is a mesotrophic system with a recent increase in cHABs. In 2018, the first cHAB was confirmed, though it is unknown if cHABs occurred decades ago prior to monitoring or more recently at non‐monitored SLRE locations. In 2021, a bloom of *Aphanizomenon flos*‐*aquae*, *Dolichospermum lemmermannii*, and *Microcystis aeruginosa* produced microcystin levels above the U.S. Environmental Protection Agency (EPA) recommended exposure threshold of 8 μg · L^−1^, resulting in a public beach closure (Lafrancois & Reinl, 2025). As for other nearshore areas around the Great Lakes (e.g., Green Bay: Bartlett et al., 2018, western Lake Erie: Allinger & Reavie, 2013; Watson et al., 2016, and Saginaw Bay: Fishman et al., 2009; Wynne et al., 2021), it has become clear that cHABs are an unresolved issue in the SLRE, but unlike for these other locations, the primary drivers remain unknown. The onset and intensification of SLRE cHABs has occurred despite decades of successful nutrient reduction and other remedial and restorative actions undertaken due to its designation as an area of concern (AOC) under the Great Lakes Water Quality Agreement (International Joint Commission, 1987). Because phosphorus, ammonium, and suspended solid concentrations have generally declined in the SLRE over the past ~50 years (Bellinger et al., 2016; Nicklay, Filstrup, et al., 2025), managers are left with few actionable targets to address cHABs.

There is currently no established cHAB‐oriented monitoring program in the SLRE, so spatiotemporal dynamics and drivers of cHABs are poorly understood. Real‐time bloom warnings and cyanotoxin testing are dependent upon happenstance observation, contrary to other modern cHAB‐affected systems with advanced remote sensing (e.g., Qiu et al., 2025) and predictive early warning (e.g., Qian et al., 2024) networks. The Lake Superior National Estuarine Research Reserve (LSNERR) conducts a monthly system‐wide monitoring program (SWMP) to routinely measure water quality, nutrients, and chlorophyll *a* (Chl *a*) at four SLRE locations during the open‐water (i.e., ice‐free) season, but the program lacks the spatial and temporal flexibility required to detect and respond to cHABs in real time and does not include routine cyanotoxin monitoring. Effective management of future cHAB scenarios requires detailed monitoring data to better understand cHAB drivers, timing, and spatial distribution. This project aimed to make recommendations for these parameters for a future SLRE monitoring program and to serve as a model for implementing a novel approach to optimize biological monitoring programs.

Monitoring programs generate time‐series data at defined spatiotemporal scales, with two important criteria being consistency in both sampling and analysis efforts and flexibility to adapt to changing objectives (Wenner & Geist, 2001). Thus, initially an aquatic monitoring program may have arbitrarily predefined sampling variables, frequency, and locations, but analysis of data generated can support refinement. Analyses of relationships between environmental variables and cyanobacterial variability can reveal which variables are the most essential to monitor in the future. Analyses of spatiotemporal redundancy in data can highlight redundant locations and can help pinpoint an optimal sampling frequency.

Our study had two overarching goals. Primarily, we sought to investigate the most important environmental predictors of cyanobacterial biovolume in the SLRE and how these predictors related to phytoplankton communities and individual species using a combination of machine learning, ordination, and lagged effect analyses. To maximize data availability, we applied an oversampling approach, characterized by the highest feasible sampling frequency, number of sites, and number of variables measured within our study period. This also facilitated the secondary goals of our study: multifaceted spatiotemporal redundancy analyses of the oversampling dataset to optimize temporal resolution, sampling window, and sampling locations in future cHAB‐oriented monitoring within the SLRE. Our approach to biological monitoring optimization may serve as a useful example to researchers and managers working in other ecological systems lacking monitoring data or needing program refinement.

## MATERIALS AND METHODS

### Project design

*Site selection*. Sampling locations were selected by a collaborative team of scientists and natural resource professionals from local, state, federal, and tribal agencies. Locations were selected based on several criteria, including previously documented cHABs, increased prevalence of eutrophication‐indicator taxa in phytoplankton communities (Alexson et al., 2018), hypoxia, and nutrient enrichment (Bellinger et al., 2016). Other considerations included proximity to impaired urban streams and stormwater outfalls and recent or planned remediation and restoration projects (Table 1). Locations also spanned anticipated water quality gradients (Figure 1). Two sites, Barker's Island and Pokegama Bay, are also LSNERR SWMP sampling locations, where continuous (15‐min) water quality and monthly nutrient and Chl *a* samples have been collected from 2013 to 2025. More detailed information on the collaborative site selection process is provided in a project‐associated technical report (Nicklay, Filstrup, et al., 2025).

**TABLE 1:** Study sites in the SLRE and their associated characteristics that informed site selection. Characterization is based on what was known prior to the study when site selection took place.

Sampling location	Site code	Hypoxia	cHAB	Nutrient enrichment	Proximal to impaired water	Current or planned remediation or restoration
Allouez Bay	AL	Y	N	Y	N	Y
Barker's Island	BA	N	Y	Y	Y	Y
Miller Creek / WLSSD	MI	N	N	Y	Y	Y
Billing's Park	BI	Y	N	N	N	N
Kingsbury Bay	KI	N	N	N	Y	Y
Pokegama Bay	PO	Y	N	Y	Y	N
Mud Lake	MU	Y	N	N	Y	Y
North Bay	NO	N	N	N	Y	Y

*Sampling design*. Routine sampling was conducted at all eight sites during the 2023 open‐water season. Sampling frequency varied with expected cyanobacteria phenology: biweekly during spring and late fall and weekly during the bloom‐prone summer months (July–early October). In 2024, open‐water routine sampling was reduced to biweekly through August 13. Winter sampling was planned monthly for both years and was completed in winter 2022–2023. Low ice cover in winter of 2023–2024 limited safe access; therefore, weekly to biweekly under ice sampling was conducted at Barker's Island and Billing's Park only. Overall, 50 routine sampling events were completed, 28 of which included all eight sites. Response sampling sets were reserved for major storm events, heat waves, or visible blooms. Herein, we use “bloom” in reference to visible accumulations of phytoplankton in the water column or at the surface, whereas we use “cHAB” in reference to confirmed toxin‐producing blooms. One additional response sample was collected due to observed high dissolved oxygen (DO) and pH levels thought to be driven by phytoplankton.

### Field and laboratory methods

#### Field sampling

All measured variables and their abbreviations are listed by collection method in Table 2. With each sample set, we collected a vertical water column profile at 1 Hz frequency using a YSI/Xylem EXO2 or EXO2s multiparameter sonde, calibrated following SWMP protocols (Mesinger, 2025). Values reported are averages of the entire vertical profile, after verification that thermal stratification was rare, with thermoclines >1°C · m^−1^ occurring in <4% of profiles. Although water level fluctuates in the SLRE, profile depths at sample locations rarely exceeded 3 m.

**TABLE 2:** All study environmental and biological variables measured or calculated, and their associated abbreviation used throughout the report.

Category	Variable	Abbreviation
Vertical Profile (YSI/EXO Sonde)	Temperature	Temp
	Dissolved oxygen concentration	DO
	Dissolved oxygen saturation	DO %
	pH	pH
	Specific conductivity	SpCond
	Salinity	Sal
	Turbidity	Turb
Surface Sensor	Carbon dioxide	CO_2_
Water Clarity & Light Attenuation	Secchi Disk Depth	Secchi
	Photosynthetically Active Radiation—Light Extinction Coefficient	PAR_kd_
Water Chemistry (1.5 m sample)	Chlorophyll *a*	Chl *a*
	Total Suspended Solids	TSS
	Volatile Suspended Solids	VSS
	Nitrate + Nitrite	NO_2_ + NO_3_
	Ammonium	NH_4_
	Dissolved Inorganic Nitrogen	DIN
	Total Nitrogen	TN
	Total Phosphorus	TP
	Soluble Reactive Phosphorus	SRP
	Total Nitrogen to Total Phosphorus Molar Ratio	TN:TP
	Total Organic Carbon	TOC
	Dissolved Organic Carbon	DOC
	Acid‐Neutralizing Capacity / Alkalinity	ANC
Phytoplankton (depth‐integrated Sample)	Phytoplankton Biovolume	

Photosynthetically active radiation (PAR) was measured using a spherical underwater quantum sensor (Li‐COR Model LI‐193) in sequence at the following depth increments: slightly above the water surface, just below the water surface, and at 50‐cm intervals from the surface to bottom. Using the Beer–Lambert law, PAR values were used to calculate the light extinction coefficient (PAR_kd_; Ingle & Crouch, 1988). Secchi disk depth (Secchi) was measured as an average of the disappear‐while‐lowering and reappear‐while‐raising water depths.

Carbon dioxide concentrations were measured using a specially modified Vaisala *p*CO_2_ sensor (Johnson et al., 2010), which was directly attached to the bottom of a float at immediate subsurface depth for uniform depth‐pressure corrections. Generally, 15–30 min of equilibration time was required for readings to stabilize at each site prior to recording values.

Discrete water chemistry samples were collected at 1.5‐m depth using a horizontal‐style Van Dorn sampler to be consistent with SWMP protocols (Mesinger, 2025). Water chemistry samples were stored on ice in the dark until laboratory arrival. Phytoplankton taxonomic samples were collected as integrated water column samples to ensure they were representative of phytoplankters distributed throughout the water column, including surface‐scum‐forming and understory low‐light‐adapted species. Phytoplankton samples were collected from the water's surface to the deepest 0.5‐m depth increment above the sediment or to a maximum of 2‐m depth. Samples were preserved with Lugol's solution in the field and stored at room temperature until microscopic analysis, which was done at random within the sample set, resulting in hold times from several days to approximately 1 year.

#### Laboratory procedures

Field samples collected for dissolved nutrients and Chl *a* were filtered in the LSNERR laboratory within 8 h of collection. The Natural Resources Research Institute (NRRI) Central Analytical Laboratory analyzed the following water quality variables following standard methods (Baird & Bridgewater, 2017). Total nitrogen (TN) and total phosphorus (TP) were measured on unfiltered water samples following potassium persulfate digestion (SM 4500‐P J) using the automated cadmium reduction (SM 4500‐NO_3_ F) and ascorbic acid (SM 4500‐P E) methods, respectively. Ammonium (NH_4_; SM 4500‐NH_3_ G) and nitrate + nitrite (NO_2_ + NO_3_; SM 4500‐NO_3_ F) were measured on filtered water samples (0.45‐μm pore size). Soluble reactive phosphorus (SRP) was measured on filtered water samples (0.45‐μm pore size) using the ascorbic acid method (SM 4500‐P E). Measured total and dissolved forms of N and P were stored frozen until analysis within recommended hold times (28 days for all dissolved forms; indefinite for total forms). Total organic carbon (TOC) and dissolved organic carbon (DOC) were measured on unfiltered and filtered water samples (0.7‐μm pore size), respectively, using a Shimadzu TOC‐L analyzer (SM 5310 B). The TOC and DOC samples were acidified with 6 N HCl to pH ≤ 2 and refrigerated in amber glass vials between collection and analysis. Acid neutralizing capacity (ANC) was measured on unfiltered refrigerated water samples using the 2‐endpoint method (SM 2320 B) within 48 h of collection. The following additional analyses were completed by the LSNERR laboratory. Total and volatile suspended solids (TSS/VSS) were measured as dry weight of particulates on GF/C filters (1.2‐μm pore size) using the loss‐on‐ignition method (SM 2540 D, SM 2540 E). Chlorophyll *a* was measured using the unacidified fluorometric method (SM 445.0) following a 24‐h, 90% acetone extraction. At project sampling sites overlapping with SWMP stations (Barker's Island and Pokegama Bay), field replicate sample sets were collected and analyzed monthly during the open‐water season. Laboratory replicates were analyzed within each analysis set for nutrients, TSS/VSS, and Chl *a* to verify accuracy of measurements. Cyanotoxin samples were collected for a small subset of bloom response samples using Wisconsin DNR‐provided kits and analyzed by the Wisconsin State Lab of Hygiene (as a part of a separately funded monitoring effort) for total microcystins and saxitoxin; cyanotoxin data from these five samples were shared with our project team by the Wisconsin DNR after laboratory analysis and QAQC were complete.

Phytoplankton taxonomic samples were analyzed at NRRI for total protists, which were primarily phytoplankton, but benthic taxa, protozoans, and zooplankton were also documented if present. The highest possible taxonomic resolution was used for identification, which resulted in a mixture of species, genera, and lesser defined groups (e.g., “unidentified coccoid chrysophyte”). Analysis followed the quantitative Utermöhl settling chamber method (Utermöhl, 1958) unless protist concentrations or debris were high, in which case a measured aliquot of sample water was placed in a Sedgewick‐Rafter counting chamber, sometimes employing dilution with 0.2‐μm‐filtered estuary water. The Utermöhl method required settling samples for at least 24 h in a chamber to concentrate protists for counting. Counting of settled samples occurred on an inverted microscope (Olympus BX51) at 400× magnification. At least 300 phytoplankton were counted per settled sample, and the transect area was recorded for quantitative calculations. To account for less common large taxa (≥30 μm; e.g., large colonies, *Ceratium hirundinella*), the full slide area was assessed at 100× magnification and all large entities were counted. In calculations, the abundance of these large taxa was downweighted by the actual area counted during 400× assessment. For taxonomic group population biovolume assessments, up to the first 10 specimens of each taxon observed were measured based on visible dimensions (length, width, diameter, depth, as necessary), cell biovolume calculations for each taxon were conducted based on standardized cell shape formulae for each taxon (Reavie et al., 2010), and then, the cell biovolume was multiplied by the number of cells in a sample. Throughout, this is referred to as biovolume (μm^3^ · mL^−1^). We follow taxonomic terminology for the genera included, using “sp.” to indicate a single, unidentified species, and “spp.” to represent a like combination of more than one species.

### Data analysis

All data analyses were performed in R (R Core Team, 2024, version 4.4.1 or 4.4.2). All manually entered electronic data were verified by a second individual for quality control purposes. Data were also examined to identify any outliers or unusual patterns from erroneous readings or recordings with standard exploratory plots (e.g., time series plots, box and whisker plots) prior to proceeding with analyses. Project data are publicly available for download (Nicklay, Reavie, et al., 2025).

#### Correlation matrix

To evaluate relationships among environmental variables and support removal of redundant variables prior to multivariate analyses, we generated a Kendall rank correlation matrix using all observations from the eight sampling sites. Kendall's tau was used due to its robustness to non‐normality and missing values. The matrix was visualized using the pairs function in R.

#### Environmental predictors of cyanobacterial biovolume

We used random forest analysis (RFA; Breiman, 2001) to predict cyanobacterial biovolume from a suite of environmental variables, assess relative importances of environmental predictors in models, and evaluate marginal predictor–response relationships over predictor distributions. Random forest analysis constructs ensembles of decision trees, with each tree built on subsets of input data and predictors, to make predictions, then self‐assesses performance through out‐of‐bag (OOB) error estimation by making predictions for observations not used in tree construction. We selected RFA because it is well suited for complex ecological datasets; it can handle many variables at once, is quite robust against multicollinearity, and can rank which predictor variables are most important, even when relationships are not linear.

Three RFA models were fit using the R function randomForest in package randomForest version 4.7.1.1 (Liaw & Wiener, 2002); a “whole estuary” model with all eight sites, an “upper‐middle estuary” model, and a “lower estuary” model. These groupings were selected based upon preliminary analyses that identified distinct water quality conditions and phytoplankton levels between the lower and upper middle regions and served to better isolate and examine predictor–response relationships within regions containing higher phytoplankton biomass. Pokegama Bay was excluded from regional subsets due to its unique, highly turbid conditions. Of 272 input samples, five had missing values for DOC, TOC, and ANC, and one of these five had missing Secchi values. Prior to all analyses, missing values were imputed with the randomForest function rfImpute, which first replaces missing values with variable medians, then fits an RFA model to the data, and finally applies weighting to the imputed values based upon similar observations. Replacements for missing data values were included in all subsequent analyses.

Cyanobacterial biovolume data were log_10_(*x* + 1)‐transformed to attain a normal distribution prior to modeling. Because RFA is robust to correlated predictors, we selected a large initial suite of 18 environmental predictors. Depth was excluded because sites were specifically selected to have uniform depth (mean = 1.22 m; *SD* = 0.47 m). Chlorophyll *a* was excluded as it was expected to directly reflect cyanobacteria as the response variable; the two were significantly correlated (*r* = 0.30, *p* = 7.6e‐14) and Chl *a* is commonly substituted as a proxy for phytoplankton biomass, which is generally elevated during cHABs. Volatile suspended solids and CO_2_ were excluded because they contained several zeroes (VSS) and missing data (CO_2_). Model arguments ntree (number of decision trees in forest), mtry (number of predictors randomly selected per split), and nodesize (number of observations defined for terminal nodes) were evaluated with a custom function and optimized from default when necessary. Predictor importances in each model were ranked by mean squared error percentage increase (%inc*MSE*, Breiman, 2001), with a higher value for a predictor indicating a greater relative decrease in model performance when it is randomly permuted. The least important predictor was iteratively dropped from subsequent model versions until model performance, as assessed by OOB mean square of residuals and percent variance explained, worsened, or did not improve. Partial dependence plots were generated with the randomForest function partialPlot to assess marginal effects of environmental variables on predicted cyanobacterial biovolume.

As a post hoc analysis in the interest of future real‐time monitoring, the relationship between Chl *a* (predictor) and cyanobacterial biovolume (response) was assessed visually and with simple linear regression. Both variables were log_10_(*x* + 1)‐transformed to obtain normality in the final regression model, and standard diagnostics plots were assessed in base R to ensure assumptions were satisfied. Regression model outputs were then superimposed onto a scatterplot of Chl *a* versus cyanobacterial biovolume with symbology denoting site and month to assess spatiotemporal variability in the relationship.

#### Phytoplankton responses along environmental gradients

To examine how the phytoplankton community related to environmental gradients, we performed a canonical correspondence analysis (CCA) using the vegan package (Oksanen et al., 2022). All routine and response sample sets with environmental and phytoplankton data were included (*n* = 272). An overabundance of environmental variables can limit the “constrained” nature of CCA, so we evaluated correlation matrices (Figure S1) to reduce explanatory variables to TP, TN, NH_4_, NO_2_ + NO_3_, DOC, DO concentration (DO), specific conductivity (spcond), pH, temperature, Secchi, and TN:TP. Predictor variables were selectively transformed with either square root or log_10_(*x* + 1)‐transformation, if necessary, to achieve approximately normal distributions. Phytoplankton community data included biovolume concentrations for 107 unique taxa. Taxa from groups containing strictly zooplankton were excluded, but some mixotrophic and/or heterotrophic species were likely present in the dinoflagellates and cryptophytes, as all taxa within these two groups were included. Several highly morphologically similar taxa were combined into single taxa, and all reported values were log_10_(*x* + 1)‐transformed to more closely approximate normality in distributions of biovolumes to satisfy CCA assumptions (Ter Braak, 1986).

Prior to CCA, we conducted a detrended correspondence analysis (DCA) using the decorana function to determine if species data were responding unimodally along environmental gradients and therefore appropriate for use in CCA. A gradient (DCA axis 1) length slightly greater than two standard deviation units (*L* = 2.01) indicated that CCA was justified (Ter Braak & Smilauer, 2002), and we proceeded with CCA. The CCA models were fit using the cca function. Variables in the environmental dataset were iteratively dropped until the final model included only environmental variables with significant effects and *VIF*s < 10 identified by the vif.cca function (Borcard et al., 2018). Total constrained variance explained by the model was reported, along with associated statistics from permutational testing (999 permutations) of the null hypothesis of no greater constrained variance in the species data explained by the environmental data than expected by chance. Total explained unconstrained variance was provided by the global model adjusted *R* ^2^. The constrained variance explained by a given axis was calculated as the constrained axis eigenvalue divided by the global total constrained inertia. Contributions of each axis and each environmental variable to explained variance in each phytoplankton taxon were calculated and tabulated. The envfit function was used to determine relative strengths and significances of individual taxon responses along the gradient each individual taxa most strongly correlated with in ordination space.

#### Lag analysis

We assessed whether environmental drivers of phytoplankton abundance were operating on temporal lags by assessing the relationship between several phytoplankton abundance proxies (Chl *a*, total algal biovolume, total cyanophyte biovolume, total diatom biovolume) and water quality variables on the day of sampling and at iterative daily intervals prior to the sampling day. Each variable from each of the eight sites was linearly interpolated over time to estimate daily values. Spearman correlations were then calculated between each phytoplankton abundance proxy value and the environmental variable on that same day, the day prior, continuing until a lag period of 50 days was reached.

#### Temporal redundancy

To assess temporal redundancy in physicochemical and nutrient variables, as well as indicators of algal abundance (Chl *a*, total algal biovolume, cyanophyte biovolume), for each variable at each site, all possible pairwise comparisons between sampling events were used to calculate the absolute difference in time between samples and the corresponding difference in variable values. This produced a full set of combinations (i.e., 1225 comparisons from 50 observations) across a temporal range from 0 to over 500 days. These data were then analyzed using LOESS (smoothed) regression to model the relationship between time difference and variable divergence. Resulting plots enabled visual assessment of when additional sampling yields diminishing returns, helping to guide optimized monitoring frequency for each environmental variable.

#### Sampling frequency reduction effects on RFA

To assess temporal redundancy in water quality–cyanobacteria relationships at the most bloom‐impacted site, an additional RFA procedure was conducted using only Barker's Island data, following the RFA methods described above. The full model used weekly data (*n* = 56), excluding winter 2022–2023 due to inconsistent sampling. Two reduced frequency models were then developed: a biweekly model using all odd‐numbered sampling events (*n* = 32) and a monthly model with manually selected events to ensure even seasonal coverage and inclusion of a late‐summer high‐cyanobacterial biovolume event (*n* = 16).

*Spatial redundancy*. As an initial step, non‐metric multidimensional scaling (NMDS) of water quality and phytoplankton data was used to identify uniqueness among sites. Based on correlation analysis (above), several variables were excluded based on either high correlation (*r* > 0.8) with another single environmental variable or moderate (0.3 > *r* < 0.8), but significant (*p* < 0.05) correlation with several other variables. This filtering process resulted in a final set of variables: TP, TN, NO_2_ + NO_3_, NH_4_, DOC, Chl *a*, spcond, pH, temperature, Secchi, and TN:TP.

The phytoplankton dataset, and therefore the corresponding NMDS, contained three fewer sample sets than the water quality dataset due to subsetting of open‐water 2024 sample enumeration. Phytoplankton biovolume (μm^3^ · mL^−1^) by taxonomic division (centric diatoms, pennate diatoms, chlorophytes, chrysophytes, cryptophytes, cyanobacteria, pyrrophytes) was ordinated using the metaMDS function in the R package vegan version 2.6.4 (Oksanen et al., 2022). Separate NMDS analyses were performed for (a) all data from the eight monitoring sites and, subsequently, for (b) summer data (July–October) so that spatial redundancy could be assessed during the likely period for cHABs. Site‐specific ellipses representing 95% confidence intervals of sample scores were used to visually identify uniqueness among sites. Ordinations were performed in three dimensions to minimize stress after evaluating scree plots, and Shepard stress plots were used to assess preservation of rank order and absolute distances among sample scores.

Sample score data from NMDS were then subjected to permutational multivariate analysis of variance (PERMANOVA) to statistically confirm uniqueness among sites, using the vegan function adonis2. Permutational multivariate analysis of variance is advantageous in this context because it optionally constrains scores by a selected “blocking variable,” so that inferred differences reflect differences constrained by sample date, much like a pairwise test but for eight sites instead of two. To justify the use of a time‐constraining blocking variable, a preliminary two‐way PERMANOVA was used to demonstrate significant independent effects of sampling event on NMDS scores for water quality (*F* = 33.95, *R* ^2^ = 0.61, *p* = 0.0001) and for phytoplankton (*F* = 2.85, *R* ^2^ = 0.26, *p* = 0.0001) along with significant independent effects of site on both water quality and phytoplankton (*F* = 57.44, *R* ^2^ = 0.27, *p* = 0.0001; *F* = 3.32, *R* ^2^ = 0.09, *p* = 0.0001). Furthermore, pairwise PERMANOVA from the R package pairwiseAdonis version 0.4 (Martinez Arbizu, 2017) provides Bonferroni‐corrected pairwise statistical comparisons (pseudo‐*F*; *p*_adj_ ɑ = 0.05) among all sites, also blocked by sampling event, to aid in identifying whether certain sites are providing redundant information about water quality and/or phytoplankton.

## RESULTS

### Correlation matrix

A correlation matrix (Figure S1) including 190 total Bonferroni‐corrected pairwise correlations among 20 environmental variables (Table 2) showed 67 significant (*p* < 0.05) positive correlations, 70 significant negative correlations, and 53 non‐significant correlations. Total and dissolved inorganic forms of nitrogen and phosphorus mostly had significant positive correlations with one another, but Chl *a* showed significant negative correlations with all forms of carbon, nitrogen, and phosphorus, except for no correlation with TP. Variables impacting light availability, including Secchi, turbidity, TSS, and PAR_kd_ were significantly correlated with one another (e.g., Secchi vs. turbidity; *r* = −0.64, *p* = 7.2e‐56). Correlations among environmental variables were considered when selecting variable sets for analyses, particularly when it was necessary to minimize multicollinearity to satisfy statistical assumptions. The final variable sets included in each analysis are detailed in the results of each analysis.

### Water quality and phytoplankton community dynamics

Total phytoplankton biovolume was lowest in the upper SLRE and highest in the lower SLRE where light and nutrients were generally most abundant (Figure S2; Table S1). In the lower estuary, Allouez Bay showed high TP, TSS, and turbidity, again consistent with red clay inputs. Miller Creek was distinguished from other lower estuary sites by persistently elevated TN, NH_4_, and conductivity, likely capturing runoff from impaired Miller Creek and wastewater discharges. Concentrations of NH_4_ at Miller Creek averaged 341 μg · L^−1^, over an order of magnitude greater than at numerous other locations (e.g., upper middle estuary sites, Allouez Bay). Total nitrogen, NH_4_, and Secchi at Barker's Island were high relative to the upper middle estuary but intermediate relative to other lower estuary sites. Most of the shallow, sheltered sites within the upper middle estuary region had similar nutrient and water quality conditions. Pokegama Bay, however, stood out from other upper middle estuary sites, with significantly higher TP, SRP, TN, TSS, and turbidity and lower DO. These differences align with the expected red clay influence from its watershed.

**FIGURE 2:**
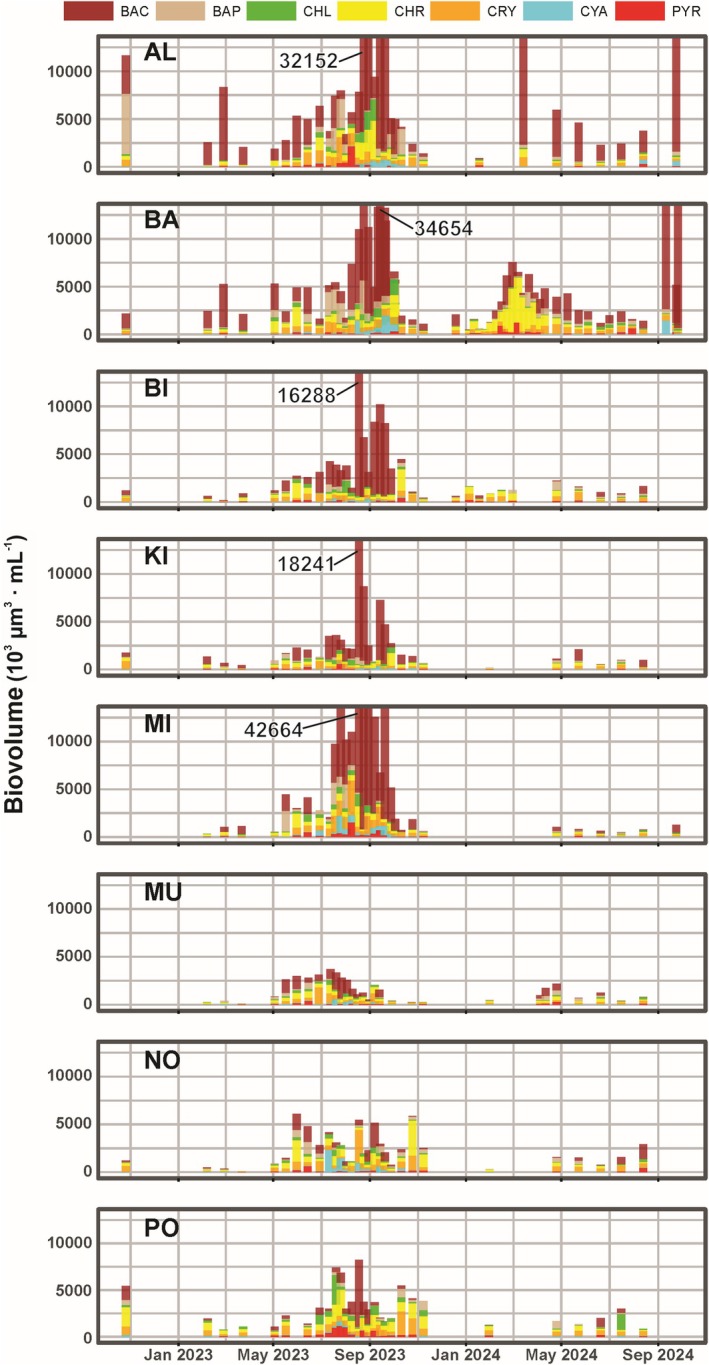
Phytoplankton biovolume in the St. Louis River Estuary (SLRE) from October 2022 to October 2024. Taxon groups are color‐coded and codes represent: BAC = centric diatoms, BAP = pennate diatoms, CHL = chlorophytes, CHR = chrysophytes, CRY = cryptophytes, CYA = cyanobacteria, PYR = pyrrophytes (dinoflagellates). To better enable visualization of phytoplankton types, some bars are cropped to minimize the effect of very high centric diatom abundance and retain the same scale in all the graphs. On cropped plots, bars corresponding to site‐specific maximum phytoplankton biovolume are labeled with biovolume values. Two letter codes for the sites are in Table 1.

Several environmental variables varied drastically between sampling years, and many showed notable seasonal patterns (Figure S2). In 2023, a summer drought resulted in an exaggerated seasonal baseflow condition that spanned June–September, over the course of which TOC/DOC declined, water clarity increased, TN:TP and nitrogen‐associated variables declined, and ANC and spcond increased. Although temperatures reached higher maxima in 2024, the duration of the seasonal thermal maximum was longer in 2023. Stacked bar plots of phytoplankton biovolume (Figure 2) identified seasonal patterns in the various taxonomic groups. In both 2023 and 2024, total phytoplankton biovolume maxima occurred in mid to late summer at all locations, with consistently higher biovolumes in 2023.

For brevity, the following description of phytoplankton groups and taxa largely focuses on the most abundant and cyanobacterial forms. Diatoms clearly dominated the phytoplankton community and displayed strong sustained summer peaks, especially in the lower SLRE (Figure 2), with biovolumes consistently surpassing cyanobacteria even during cHABs. Centric diatoms were ubiquitous at nearly all times of year and across sites, especially the cosmopolitan generalists *Aulacoseira ambigua* and *Au. granulata*, the former having the highest total biovolume among all phytoplankton taxa in our dataset. Pennate diatoms (e.g., *Nitzchia* spp., *Fragilaria* spp.) also frequently dominated communities or co‐dominated with centric diatoms but displayed strong seasonal patterns and species‐specific variability between years (Figure 2). Chrysophytes were prevalent and diverse across sites and seasons, with higher abundances in summer 2023 than in 2024. Although chlorophytes, cryptophytes, and pyrrophytes rarely dominated communities, they each were present and diverse at relatively low biovolumes in most samples and were generally most abundant in summer months (Figure 2).

Cyanobacteria were taxonomically diverse and exhibited varied spatial and seasonal distributions, including both late summer, bloom‐forming taxa and ambient taxa that occurred at low abundance during non‐bloom periods. Cyanobacteria were present in all collected samples, but peaked in July–September, particularly in the lower SLRE where their populations were sustained for longer periods (Figure 2). The highest observed cyanobacterial biovolumes occurred in samples from Barker's Island, though not all of these were bloom‐response samples; for example, the third highest observed cyanobacterial biovolume occurred in a routine sample collected at Barker's Island on August 18, 2023. At some upper middle SLRE sites, including North Bay, sparsely distributed short‐term peaks were observed. Cyanobacterial biovolume and Chl *a* showed a weak but significant positive relationship (*m* = 0.76, *b* = 3.90, *F*_1,27_ = 65.11, *R* ^2^ = 0.19, *p* = 2.34e‐14; Figure S3).

In total, 19 bloom‐response samples were collected across seven bloom events, triggered by visible cyanobacteria aggregations or surface scums. The timing of cyanobacterial blooms in 2023 and 2024 was similar and aligned with previously observed blooms (Lafrancois & Reinl, 2025). In 2023 and 2024, discrete, small‐scale surface blooms and subsurface macroscopic planktonic cyanobacterial colonies were observed in late August. By early to mid‐September in 2023 and 2024, several small‐ to medium‐sized blooms occurred, all as short‐lived surface scums in the morning when winds were calm, and dissipated by afternoon with increased winds. However, two spatially extensive blooms that persisted throughout a day or on subsequent days occurred in fall 2023 (September 20–21 and October 2–3, 2023). To our knowledge, the areal coverage of scums in the SLRE during these blooms was unprecedented and included the first scums documented upstream of the lower SLRE. The diazotroph *Aphanizomenon flos‐aquae* was the dominant genus of cyanobacteria in all 2023 blooms (Figure S4), and suspended flake‐like colonies were frequently observed in the lower SLRE in weeks precluding blooms. In contrast, 2024 blooms had lower overall cyanobacterial biovolume but more diverse communities lacking clear dominant taxa (Figure S4), often including multiple *Dolichospermum* species and the non‐diazotroph *Microcystis aeruginosa* in addition to *A. flos‐aquae*. These blooms appeared more abruptly and were more localized and ephemeral compared to those in 2023 but included new locations in the SLRE. Low levels of total microcystins and/or saxitoxins were detected at Barker's Island during three events (September 21, 2023; October 3, 2023; August 21, 2024; Table S2). All toxin values were below the World Health Organization recreational thresholds, but one result for total microcystins exceeded EPA drinking water guidelines (Table S2).

In addition to the planktonic and surface blooms observed, benthic cyanobacteria *Oscillatoria* sp., and to a lesser extent, *Phormidium* sp., were observed at locations proximal to sampling sites. Filamentous mats were aggregated densely on the benthos and eventually detached, likely buoyed by photosynthetic oxygen production on calm, sunny days. This was a novel observation in the SLRE. Planktonic genera *Synechococcus* sp., *Chroococcus* sp., and *Pseudanabaena limnetica* were ubiquitous, occurring commonly throughout the SLRE in all seasons. *Planktothrix* sp. cf. *agardhii*/*rubescens* was also present as a non‐dominant species in several routine, bloom, and winter samples. Last, some species including *Trichodesmium lacustre*, *Cuspidothrix issatschenkoi*, *Snowella* sp., *Woronichinia* sp., and *Sphaerospermopsis aphanizomenoides* were observed fewer than five times (Figure S4).

#### Environmental predictors of cyanobacterial biovolume

Cyanobacterial biovolume was highest and best predicted by environmental variables in the lower estuary (Table 3), corresponding to the region where blooms produced visible surface scums during the study period. Whole estuary and combined upper middle estuary (Table 3) RFA models had moderate performance. Iteratively dropping TN:TP and TSS yielded better performance in the combined upper middle estuary model, but in both other models, all initial predictors were retained as dropping the least important decreased performance. The Barker's Island‐only full model had the highest *R* ^2^ among all models (0.66).

**TABLE 3:** Relative importance of environmental predictors in random forest models of cyanobacterial biovolume, ranked by percent increase in mean squared error (%Inc*MSE*).

Model parameter	Whole estuary	Upper mid estuary	Lower estuary	BA	BA bi‐weekly	BA monthly
*n*	272	124	119	56	32	16
*MSE*	0.29	0.27	0.29	0.25	0.29	0.39
*R* ^2^	0.49	0.46	0.58	0.66	0.4	0.22
**predictor**						
temp	**1**	8	**1**	8	8	**2**
DO	**2**	6	7	10	9	**1**
TOC	**3**	**5**	**4**	**2**	**1**	7
pH	**4**	14	**2**	13	15	14
DOC	**5**	7	8	**3**	**5**	9
TN	6	**2**	**3**	**1**	**2**	**5**
NO_2_ + NO_3_	7	**1**	6	6	**4**	**3**
spcond	8	9	9	12	11	8
ANC	9	12	**5**	17	10	10
TN:TP	10	NA	10	**5**	**3**	**4**
PAR_kd_	11	**3**	14	9	6	6
NH_4_	12	**4**	12	7	13	13
DO %	13	16	11	14	14	11
TP	14	10	18	15	NA	15
Secchi	15	11	15	18	16	17
SRP	16	15	17	11	12	NA
TSS	17	NA	13	**4**	7	12
Turbidity	18	13	16	16	NA	16

*Note*: Rankings are shown for the whole estuary model, combined upper middle estuary model, and lower estuary model. NA indicates that the predictor was removed during iterative variable reduction in the final model. Three additional models are presented using all Barker's Island (BA) data (approximately weekly sampling), and subsets of bi‐weekly and monthly sampling for that site. Model parameters included are number of sampling dates (*n*), mean squared error of prediction (*MSE*), and multiple correlation coefficient (*R* ^2^). Bold values indicate predictors ranked by MSE, in accordance with standard reporting for RFA. These rankings do not have *p*‐values or *F*‐statistics associated with them, as, for example, predictors in multiple regression would.

In the whole estuary model, temperature was the most important predictor (19.3 %Inc*MSE*), followed by DO (14.6 %Inc*MSE*), TOC (14.4 %Inc*MSE*), and pH (14.1 %Inc*MSE*). In the lower estuary model, temperature was again the most important predictor (17.6 %Inc*MSE*), followed by pH (12.9 %Inc*MSE*), TN (11.3 %Inc*MSE*), and TOC (10.7 %Inc*MSE*). Contrary to the other models, NO_2_ + NO_3_ (14.0 %Inc*MSE*) was the most important predictor in the combined upper middle estuary model, followed by TN (13.7 %Inc*MSE*), PAR_kd_ (11.3 %Inc*MSE*), and NH_4_ (8.4 %Inc*MSE*). Total phosphorus and SRP were among the least important predictors in all three regional models. Acid‐neutralizing capacity and spcond had moderate to low predictive importance in all models. In the Barker's Island‐only all‐observations model, TN was the most important predictor, followed by DOC/TOC, TSS, and TN:TP.

**FIGURE 3:**
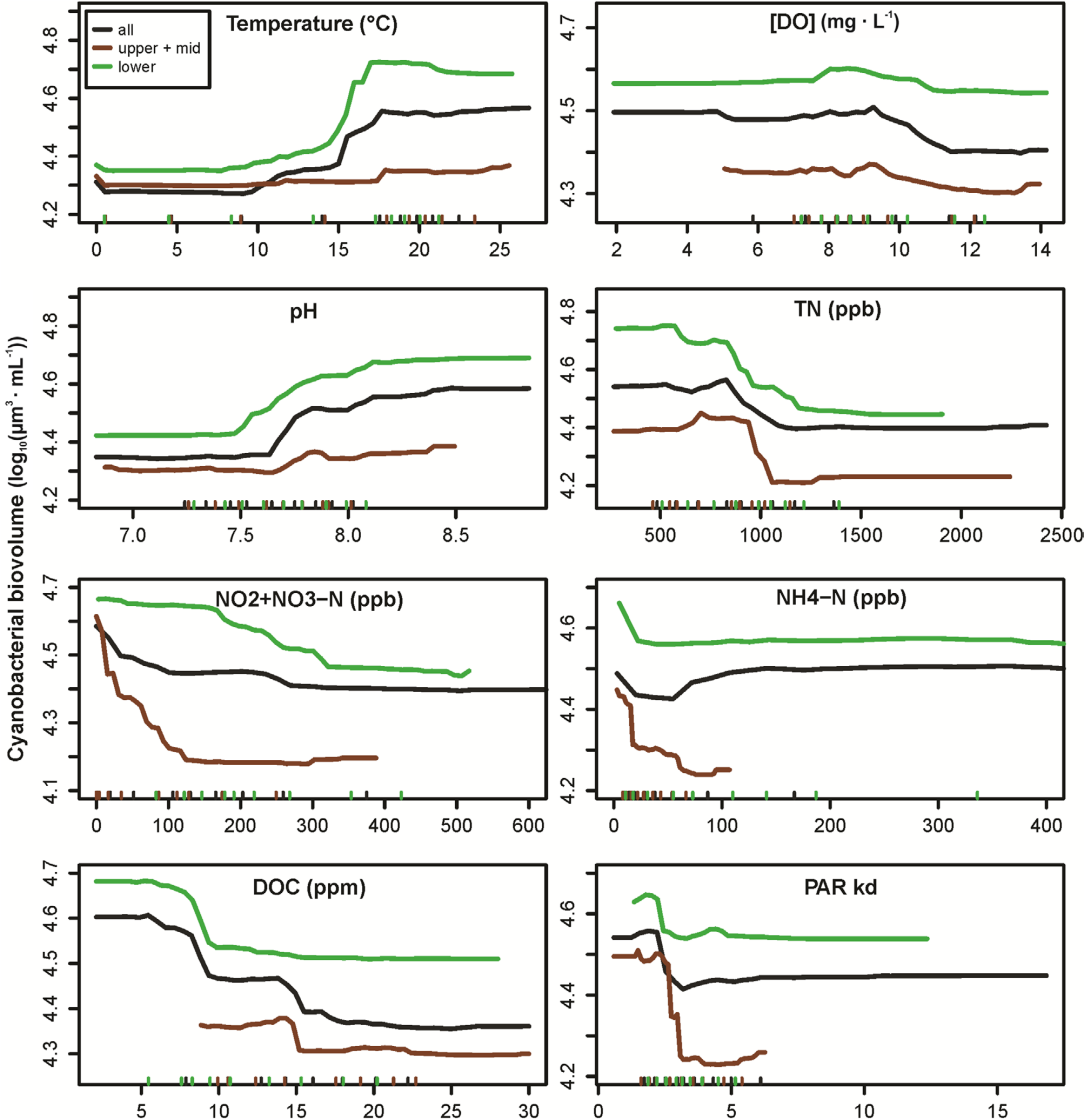
Partial dependence plots showing the marginal effects of selected environmental variables had on the predicted cyanobacterial biovolume in a random forest machine learning model. Models were separately developed for all sites and two subdivisions (upper mid, lower SLRE; Figure 1).

**FIGURE 4:**
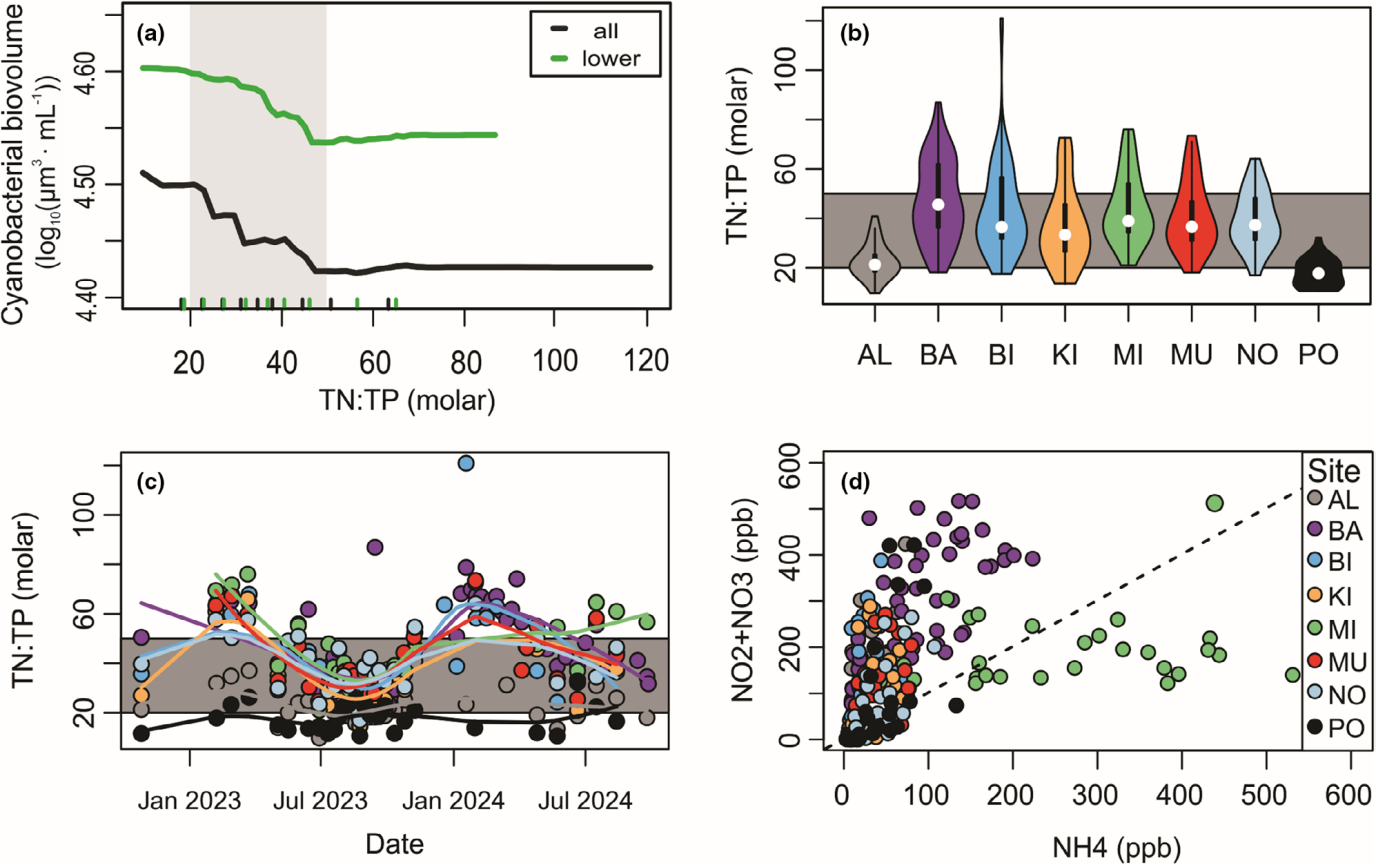
(a) Partial dependence plot showing the marginal effects of TN:TP on predicted cyanobacterial biovolume in a random forest machine learning model. Models were separately developed for all sites and the lower St. Louis River Estuary (SLRE); Figure 1). (b) Violin plot showing distributions of TN:TP at the eight sampling stations over the duration of the project (October 2022 to September 2024). (c) Time‐series plot of TN:TP at the eight sampling stations over the duration of the project, plotted as points with a LOESS smoothed line (default *span* = 0.75). (d) Biplot of NO_2_ + NO_3_ versus NH_4_ with points color‐coded by site and a 1:1 reference line, illustrating the NO_2_ + NO_3_‐dominated DIN pool at most SLRE sites at most times of year.

The marginal effects of individual predictors on cyanobacterial biovolume were similarly patterned across different regional RFA models, although the exact thresholds and magnitudes of effects differed (Figure 3; Figure 4a). In all three regional RFA models, temperature and cyanobacterial biovolume related positively, with a sharp increase in predicted biovolume above ~15°C. By contrast, total and dissolved inorganic forms of nitrogen had generally negative marginal effects, particularly in the lower estuary. Similarly, in the whole and lower estuary models, TN:TP had negative marginal effects, with the highest predicted cyanobacterial biovolume occurring in nitrogen limited (TN:TP < 20) ranges. Year‐round TN:TP distributions did not vary strongly among sites (Figure 4b) but did show strong temporal fluctuations (Figure 4c) driven by TN and the dissolved inorganic nitrogen (DIN) pool, which was generally NO_2_ + NO_3_‐dominated and rarely depleted (Figure 4d). Dissolved organic carbon and PAR_kd_, which decreased during the 2023 drought as clarity increased, had negative marginal effects in all regions, indicating maximum predicted cyanobacterial biovolume coincided with less‐stained water and greater light penetration. Maximum predicted cyanobacterial biovolume occurred at intermediate DO ranges (~8–11 mg · L^−1^) and plateaued above pH of ~8.0.

### Phytoplankton responses along environmental gradients

**FIGURE 5:**
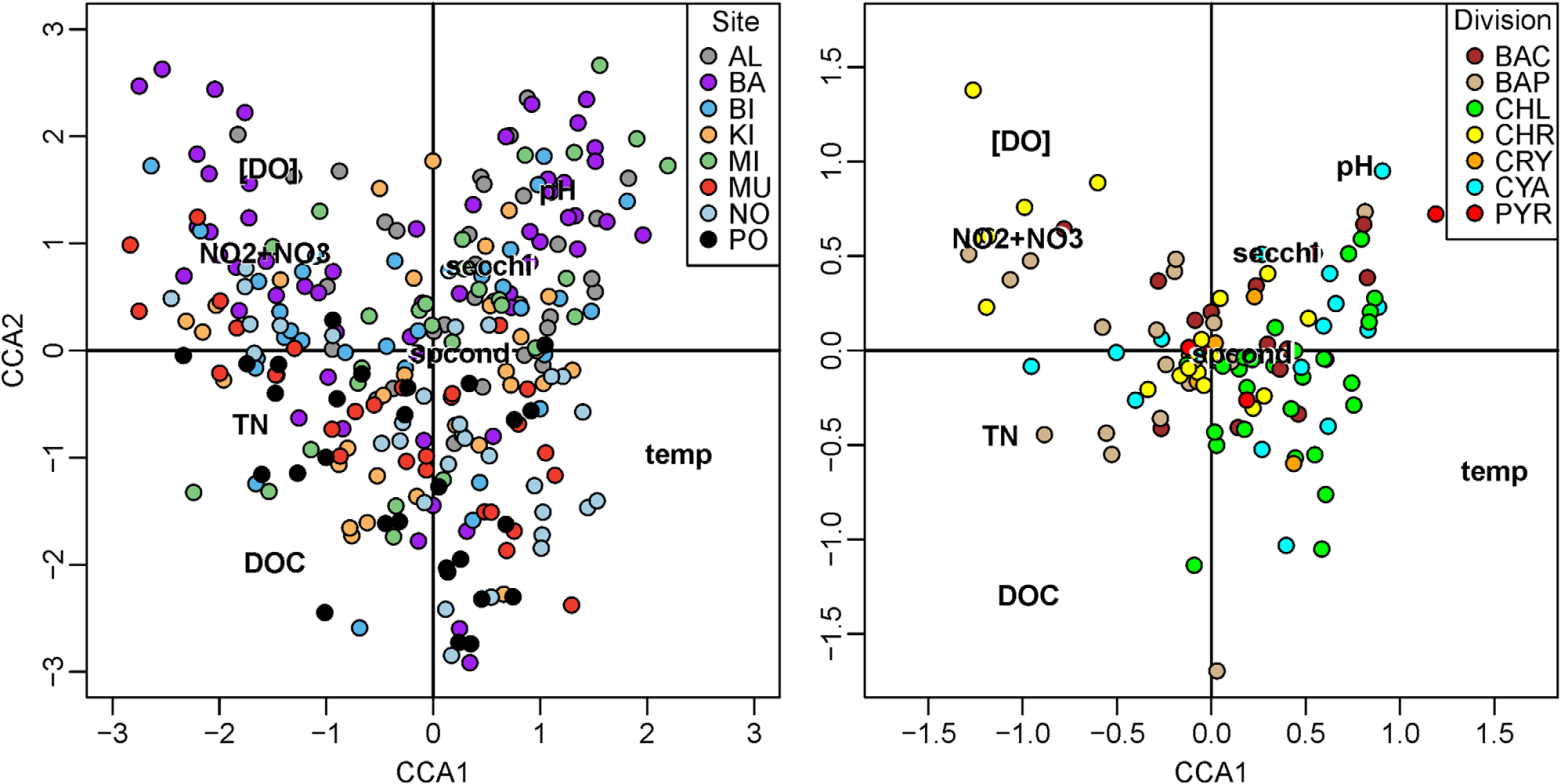
CCA (*R* ^2^_adj_ = 0.06; *chi‐square* = 0.36; df = 8; *F* = 3.14; *p* = 0.001) site scores (left) and species scores (right) on axes CCA1 (2.19% of total var explained) and CCA2 (1.01% of total variance explained) depicting ordination of individual samples and phytoplankton taxa along unimodal environmental gradients. Taxon groups are the same as for Figure 2. Two letter codes for the sites are in Table 1. Statistics on CCA axes, environmental variable effects, and phytoplankton taxa responses are included in Table 4, Table 5, and Table S3.

The first two axes of the CCA (Figure 5), respectively, captured 2.19% and 1.01% of the variation in the phytoplankton data as well as 53.98% of the cumulative constrained phytoplankton–environmental relationship; the total explained variation (*R* ^2^_adj_) was 5.94% when converted from raw total constrained inertia (chi‐square = 0.36, *df* = 8). Associated permutation testing indicated that the explained phytoplankton variation by environmental variables was significant (*F* = 3.14, *p* = 0.001). The first six of eight CCA axes significantly contributed to the explained variation (*F*‐test, *p* < 0.05; Table 4) and collectively explained 5.81% of the total phytoplankton variation along with 92.87% of the cumulative constrained phytoplankton–environmental relationship. Axis 1 represents a gradient capturing temperature and pH as positive values and higher nitrogen in the negative. Axis 2 separates scores based on higher Secchi and pH in the positive and higher DOC in the negative. Specific conductivity occurs at the origin of this two‐dimensional plot, but it has a large negative loading on axis 3 (Table 5).

**TABLE 4:** All (8) axes of a CCA examining phytoplankton responses across unimodal environmental gradients (*R* ^2^_adj_ = 0.06; *chi‐square* = 0.36; *df* = 8; *F* = 3.14; *p* = 0.001).

Constrained Axis	*df*	Eigenvalue	% tot. Variance	% constr. Variance	*F*	*Pr*(>*F*)
CCA1	1	0.134	3.53%	37.02%	9.266	0.001
CCA2	1	0.062	1.63%	17.13%	4.285	0.001
CCA3	1	0.056	1.47%	15.47%	3.845	0.001
CCA4	1	0.039	1.03%	10.77%	2.707	0.001
CCA5	1	0.024	0.63%	6.63%	1.694	0.012
CCA6	1	0.022	0.59%	6.08%	1.517	0.034
CCA7	1	0.013	0.34%	3.59%	0.930	0.93
CCA8	1	0.012	0.32%	3.31%	0.859	0.843
Residual	263	3.801	NA	NA	NA	NA

*Note*: The first six axes significantly contribute to the explained variance (*p* < 0.05), constituting 92.87% of the cumulative constrained phytoplankton–environment relationship. The proportion of total variance explained by a particular axis is obtained by dividing the axis eigenvalue by total unconstrained inertia (“Residual,” 3.801), while the proportion of constrained variance explained by a particular axis is calculated by dividing an axis eigenvalue by the sum of tabulated eigenvalues (0.362).

**TABLE 5:** All (8) environmental variables included in final CCA (*R* ^2^_adj_ = 0.06; *chi‐square* = 0.36; *df* = 8; *F* = 3.14; *p* = 0.001) and their contributions.

Variable	*df*	chi‐square	*F*	*Pr*(>*F*)	CCA1	CCA2	CCA3
DOC	1	0.08	5.83	0.001	−0.571	−0.762	0.267
DO mg · L^−1^	1	0.08	5.87	0.001	−0.594	0.646	0.065
spcond	1	0.04	2.90	0.001	0.096	−0.018	−0.690
pH	1	0.04	2.80	0.001	0.448	0.567	0.001
temp	1	0.03	2.16	0.001	0.882	−0.384	0.187
sqrt TN	1	0.04	2.56	0.001	−0.659	−0.265	0.411
sqrt Secchi	1	0.02	1.68	0.001	0.202	0.305	−0.375
sqrt NO_2_ + NO_3_	1	0.02	1.31	0.042	−0.604	0.350	0.474
Residual	263	3.80	NA	NA	NA	NA	NA

*Note*: Environmental variables were individually transformed prior to analysis to attain normal distributions and were iteratively dropped until only variables with significant (*p* < 0.05) response gradients remained. Higher chi‐square for a variable indicates stronger responses by phytoplankton across the gradient represented by the respective variable. Scores for the first three CCA axes are included for each environmental variable, representing the strongest orientations in ordination space.

There was separation among sampling sites and individual observations reflecting both spatial and seasonal variability (Figure 5). Barker's Island had conditions spreading across both major CCA gradients (axes 1 and 2), and a distinct cluster of observations projecting far into the upper left quadrant reflected its especially high DO and NO_2_ + NO_3_ in the winter and early spring. Barker's Island and Billings Park were sampled much more frequently than other sites due to conditions preventing access in winter 2024, so additional sites may have experienced similar conditions. Miller Creek was distributed similarly to Barker's Island but was less extreme in terms of DO and NO_2_ + NO_3_, though it had some cases in the lower left quadrant, reflecting high DOC and nitrogen. Allouez Bay tended to be more centrally located, overlapping with Miller Creek and Barker's Island. Billings Park mainly occupied the upper quadrants, indicating higher pH, Secchi, DO, and NO_2_ + NO_3_. Kingsbury Bay had a similar distribution, but extended into the lower quadrants, likely due to occasionally higher DOC. North Bay and Mud Lake represented a gradient stretching from the upper left quadrant (higher nitrogen) and the lower right (higher temperatures); they did not encroach on the upper right gradient representing higher pH. Pokegama tended to be constrained to the lower quadrants, driven largely by higher DOC.

All selected environmental variables had significant individual effects in the CCA (*F*‐test, *p* < 0.05; Table 5). The ranking of the contributions of environmental variables to explained variance was notably distinct from the ranking of environmental predictor importance in the RFA models built to predict cyanobacterial biovolume, indicating that the most important environmental variables related to overall phytoplankton community composition in the SLRE are not the same variables related to cyanobacterial abundance. For instance, TN and NO_2_ + NO_3_, which were both moderate to important RFA predictors of cyanobacterial biovolume with negative marginal effects, had significant but relatively weak effects on overall community composition based on their CCA axis loadings.

Of the 107 phytoplankton taxa included in CCA, 78 significantly responded along environmental gradients (*R* ^2^, *p* < 0.05; Table S3; Figure S5). Although there were too many species‐specific trends to explain in detail, we highlight some of the most notable outcomes. Abundant bloom‐forming cyanophytes such as *Aphanizomenon flos‐aquae*, *Dolichospermum* sp. cf. *circinale/lemmermannii*, and *Microcystis aeruginosa* occurred in association with higher temperatures and pH and lower nitrogen in the upper right quadrant, which captures summer in the lower estuary sites. Within these three genera, all species responded significantly along environmental gradients, and *A. flos‐aquae* had the second highest explained variance among all phytoplankton species (45%; *p* = 0.001). Clear separation in algal groups by season manifested as the majority of green and cyanobacterial taxa occupying the right quadrants (Figure 5; Figure S5), reflecting higher temperature optima and frequent co‐occurrence with seasonal TN minima and light availability maxima. Prevalent winter taxa, including some pennate diatoms and chrysophytes, generally grouped in the left quadrants (Figure 5; Figure S5) coincident with low temperature and higher DO and NO_2_ + NO_3_, where ubiquitous generalists such as *Aulacoseira ambigua* occurred near the origin.

### Lag analysis

**FIGURE 6:**
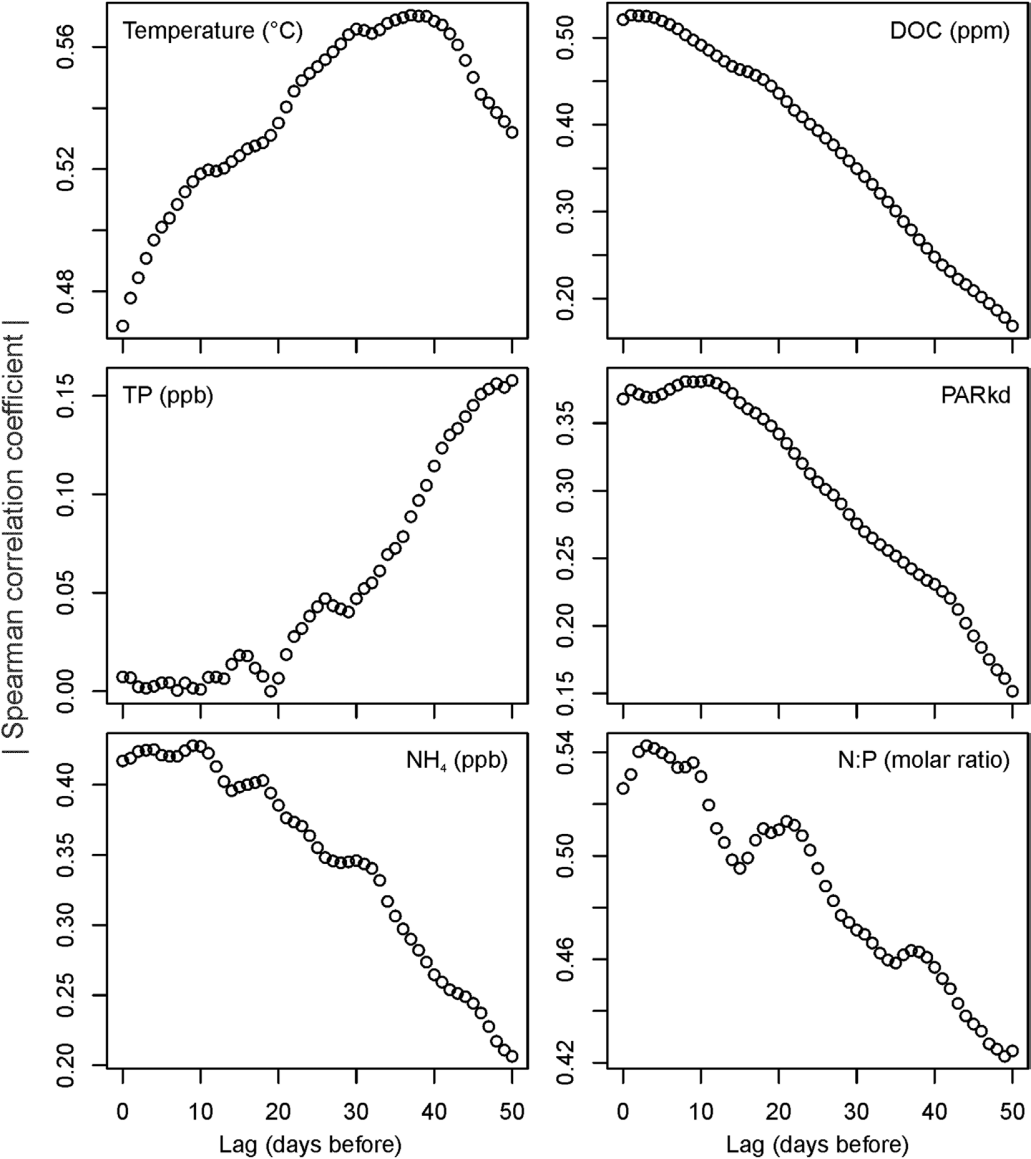
Lag analysis of the relationships between selected environmental variables and cyanobacterial biovolume. Lags in environmental data measurements from 0 to 50 days prior to cyanobacterial measurements were explored using the absolute value of the correlation coefficient between the variable and biovolume. A complete set of lag analysis plots are provided in Figure S6.

A linear method was used to interpolate daily values, so these findings should be interpreted with caution, as some variables may change nonlinearly. For environmental variables that change in a predictable manner over time, such as temperature and DOC (Figure S2), interpolation is more likely to reflect true values. Based on the lag analysis (Figure 6; Figure S6), most variables were best correlated with cyanobacterial biovolume when same‐day data were used. For example, the cyanobacteria–DOC correlation coefficient declined steadily with increasing time lag. A notable exception was the optimal temperature–cyanobacteria relationship, which suggested that water temperature 30–40 days prior had a stronger predictive relationship to cyanobacterial biovolume than a same‐day measurement. Lag analyses for variables NH_4_, TN:TP, and PAR_kd_ indicated that their measurements within ~10 days prior were indicative of cyanobacterial abundance, with TN:TP possibly optimal 4 days prior. Lag analysis for TP showed no meaningful correlations with cyanobacterial abundance, and although correlation increased slightly from 20 to 50 days prior, the relationship remained weak.

### Temporal redundancy in sampling

#### Barker's Island‐specific analysis

The Barker's Island RFA temporal subset analysis (Table 3) shows that weekly sampling yielded the highest model performance (*R* ^2^ = 0.66) and lowest predictive error (*MSE* = 0.25), indicating that higher frequency sampling better captures variability in cyanobacterial abundance. Bi‐weekly sampling showed a notable drop in *R* ^2^ (0.40), but only a slight increase in predictive error (*MSE* = 0.29), and shared a similar set of top predictors: TN and TOC remained highly ranked, with temperature relatively low in importance. This result suggests that bi‐weekly sampling is sufficient to capture major predictors of cyanobacterial biovolume. Monthly sampling, by contrast, resulted in temperature and DO emerging as the top predictors, reflecting a shift toward more seasonal patterns rather than ephemeral patterns in cyanobacteria. Marginal effects of predictors, which were evaluated but not included in this manuscript for Barker's Island‐only models, remained generally consistent across models despite changes in predictor importance and overall model performance.

#### Absolute differences versus days between measurements

**FIGURE 7:**
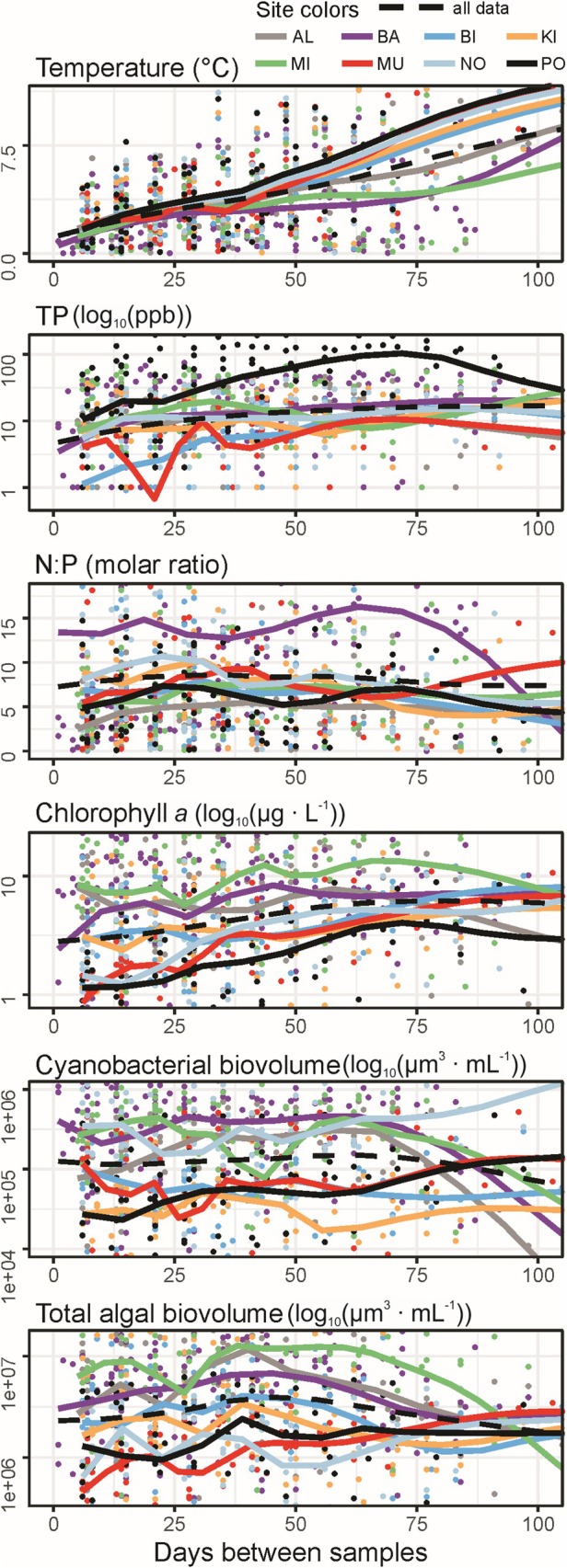
Analysis of the relationship of time between samples (x‐axis) versus associated differences in variable data (*y*‐axes). Each point in the resulting plots represents a pairwise comparison between two sampling events at the same sampling site. Analyses include summer (July–October) data, and locally weighted (LOESS) curves (*span* = 0.3) for each site and for all data combined summarize the relationships. A 1:1 relationship indicates linear convergence in a variable's value as the days between samples approaches zero, while a horizontal relationship indicates equal differences between sample values regardless of the amount of time between their collection.

We examined how differences in environmental variables and phytoplankton group biovolumes changed as time between sampling events increased to evaluate temporal redundancy in the dataset (Figure 7). For environmental variables such as temperature and nutrients, results showed no consistent minimum sampling interval. As expected, smaller differences were observed between closely spaced samples, with variability generally increasing as the time between sampling events grew. This pattern is most easily visible for temperature, which clearly converged toward 0 at lower time intervals. However, some environmental and biological variables (e.g., TN:TP; total phytoplankton) did not follow this pattern and, instead, showed more consistent or seemingly random differences between samples regardless of the amount of time between their collection. In many cases, particularly for biological indicators, temporal variability remained high regardless of the time interval between measurements.

### Spatial redundancy

#### Non‐metric multidimensional scaling ordination

**FIGURE 8:**
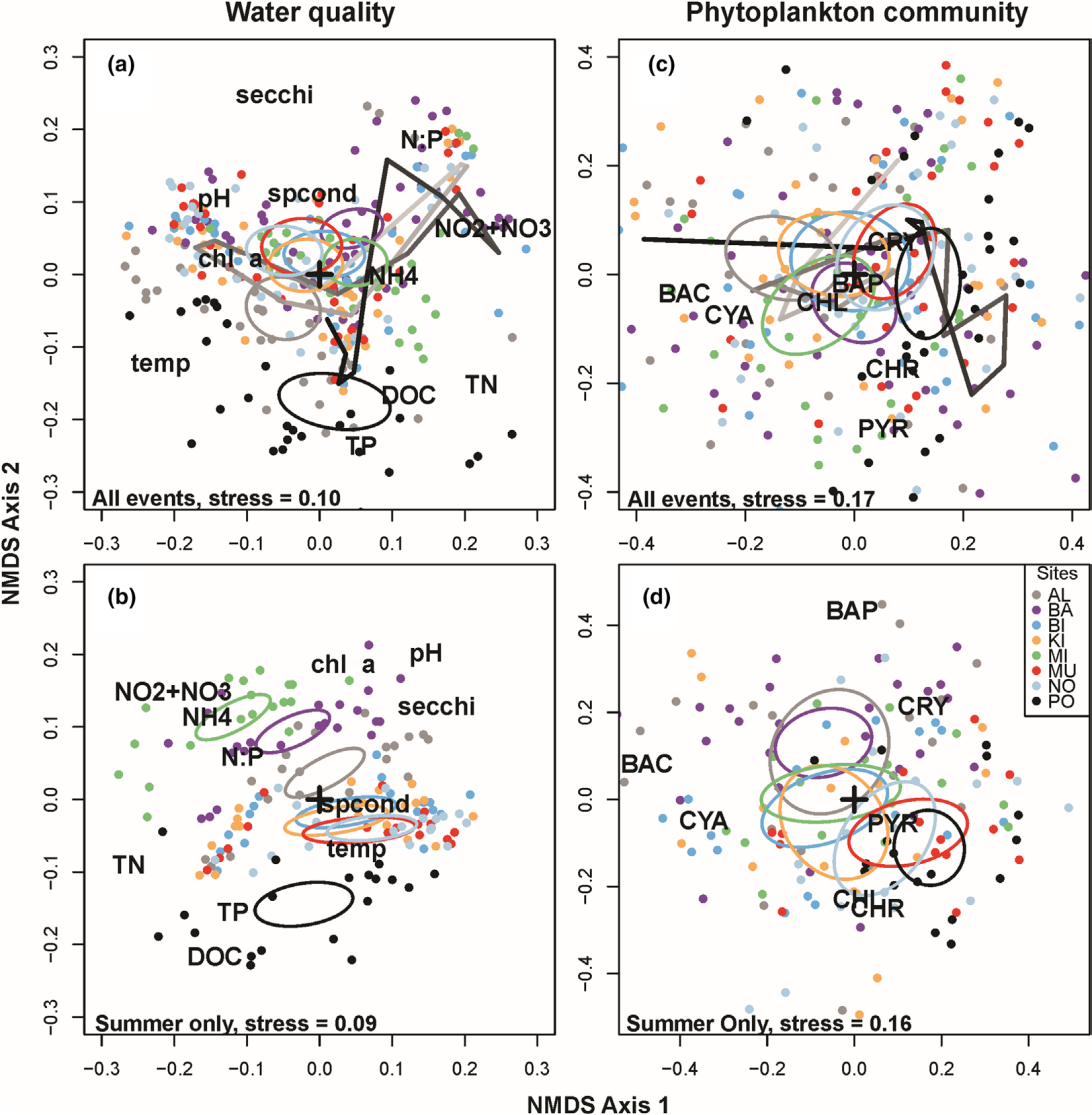
Non‐metric multidimensional scaling analyses of water quality (left plots), phytoplankton (right plots), either viewing all data (October 2022–October 2024; upper plots) or summer season only (July–October 2023; lower plots). Sample scores are shown as points while variable centroids are shown as text. Colored ellipses represent the two‐dimensional, 95% confidence interval of that site's sample scores. All‐event plots include monthly sample centroids connected by grayscale lines from earliest (light gray) to latest (black), to track temporal trajectory of scores. Two letter codes for the sites are in Table 1 and taxon group codes are the same as for Figure 2. Axis 3 plots are provided in Figure S7.

Non‐metric multidimensional scaling provided insights into spatial redundancy in environmental conditions and phytoplankton communities. Separate ordinations were done for environmental variables (Figure 8a,b) and for phytoplankton communities (Figure 8c,d) and, within these subsets, for all samples (Figure 8a,c) and for summer samples (Figure 8b,d) only. Three axes were included for the NMDS, as ordination stress approached a minimum asymptote at approximately this number; plots including axis 3 are included as supplements (Figure S7), as axes 1 and 2 illustrated the strongest patterns in the data. For summer water quality (Figure 8b), Pokegama Bay stood out with elevated TP, TN, and DOC, while Miller Creek and Barker's Island were characterized by higher Chl *a*, pH, and nitrogen‐related variables (NO_2_ + NO_3_, NH_4_, TN:TP). For summer phytoplankton (Figure 8d, stress = 0.16), Allouez Bay, Barker's Island, Billings Park, and Miller Creek were more associated with cyanobacteria and centric diatoms, whereas North Bay, Mud Lake, and Pokegama Bay supported more diverse assemblages of chrysophytes, dinoflagellates, and chlorophytes. Although phytoplankton communities overall showed greater similarity among sites than water quality metrics, upper estuary sites and Pokegama Bay consistently separated from the lower estuary. Site clusters were less distinct in the water quality and phytoplankton community plots when year‐round samples were included (Figure 8a,c), suggesting less pronounced heterogeneity in conditions and communities from autumn through spring in the system.

#### Multivariate assessment

**FIGURE 9:**
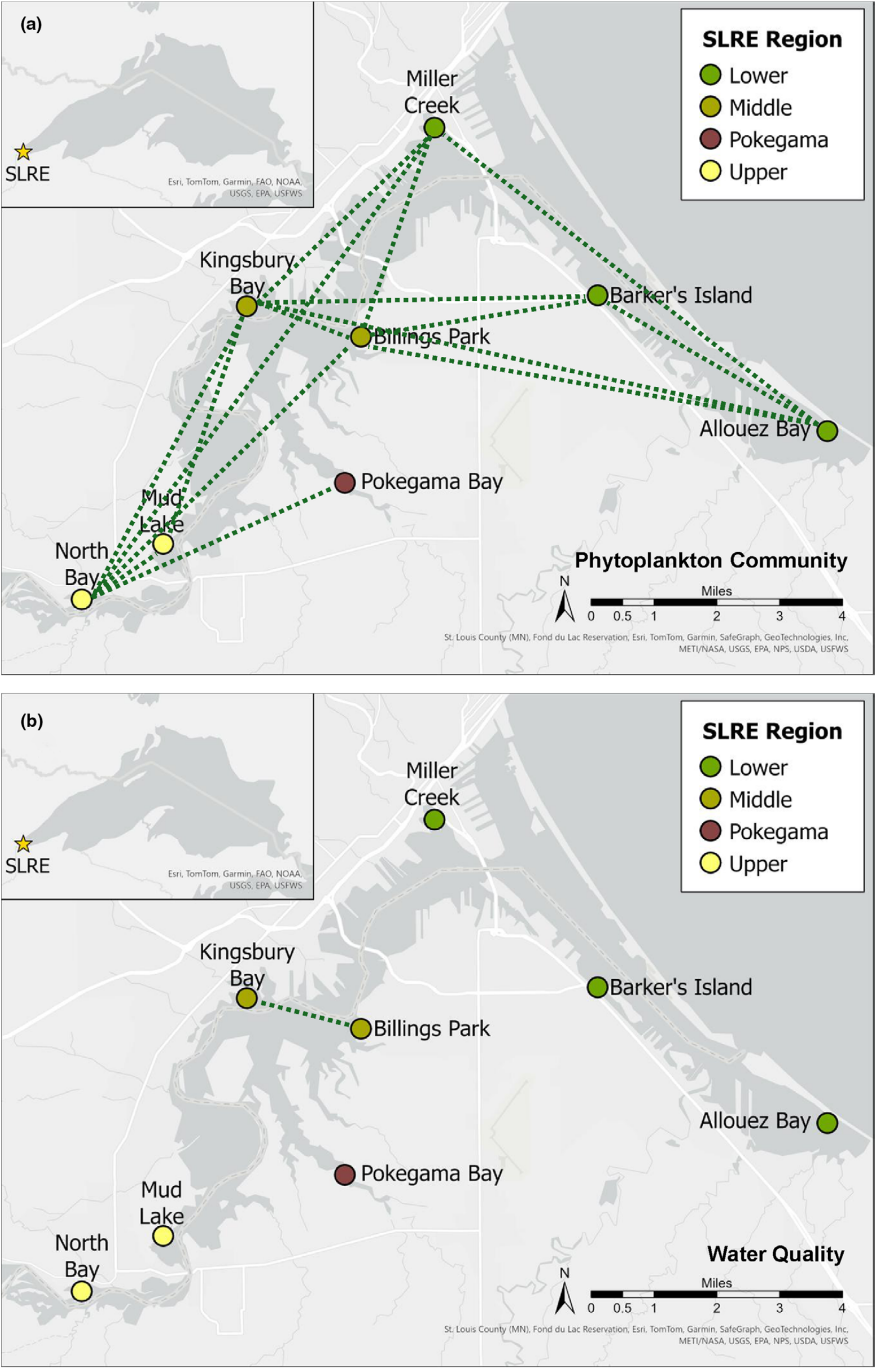
Similarities among sample sites based on a PERMANOVA of 1) phytoplankton communities and 2) water quality in July–October samples. Connections by dashed lines indicate no significant difference (*F*‐test, *p* > 0.05) in pairwise site comparisons of NMDS sample scores.

A one‐way PERMANOVA on NMDS sample scores showed significant spatial heterogeneity in water quality (*nperm* = 9999, *df* = 7, *F* = 11.2, *p* < 0.0001) and in phytoplankton communities (*nperm* = 9999, *df* = 7, *F* = 2.7, *p* < 0.0001). Pairwise PERMANOVA using the full dataset illustrated that phytoplankton communities were much more similar across sites than across environmental conditions (Figure 9), with many more connections between non‐distinct site pairs for the former. Only one of 28 pairwise site comparisons did not reveal distinct water quality, and 15 did not have distinct phytoplankton communities. Several pairs of sites had distinct water quality and phytoplankton communities from one another, but no single site had both distinct water quality and distinct phytoplankton communities from all other sites in the whole dataset.

## DISCUSSION

### Ecological investigation

A detailed investigation for the St. Louis River Estuary aided in describing the complex and climate‐influenced interplay between temperature and nutrients that has facilitated recent cHABs within the system. Hydrologic conditions (indicated by TOC/DOC), nitrogen (approximated by TN and its ratio with phosphorus), and temperature were key predictors of cyanobacterial biovolume. Each variable reflects broader ecological conditions and strong seasonal gradients. Given the known reduction of anthropogenic nutrient inputs to the SLRE (Bellinger et al., 2016), it is likely that future bloom dynamics will be shaped by climate‐driven shifts in temperature and hydrology that ultimately drive concurrent stoichiometry.

#### Cyanobacterial biovolume predictors

Results suggest that drought‐driven stoichiometric N‐limitation occurring in concert with anomalously high late‐summer water temperatures facilitated large, *Aphanizomenon*‐dominated blooms in 2023. As a post hoc classification framework for discussion regarding TN:TP state, we use “N‐limitation” in the stoichiometric context (i.e., TN:TP < 20; Guildford & Hecky, 2000), whereas we use “N‐depletion” to refer to instances in which the sum of measured DIN forms is at or near non‐detectable levels. Summer baseflow conditions were generally N limited to balanced (20 < TN:TP < 50; Guildford & Hecky, 2000), and sustained NO_2_ + NO_3_ and/or NH_4_ depletion was only observed during the drought of summer 2023, primarily in the upper SLRE. This observation explains the high predictive value of these RFA variables in the upper middle SLRE model and the ephemeral dominance of diazotrophs (e.g., *A. flos‐aquae, Dolichospermum crassum*) in 2023. In the SLRE, seasonal baseflow typically occurs annually in the late summer through early fall; during this period, upriver discharge entering the SLRE is relatively low and is particularly low when prolonged drought coincides with annual baseflow, such as in 2023. This phenomenon promotes high denitrification rates in the lower SLRE (Loken et al., 2016), but continual delivery of NO_2_ + NO_3_ from adjacent Lake Superior probably replaces associated gaseous losses and may explain the general lack of NO_2_ + NO_3_ depletion at lower SLRE sites.

The most intense blooms of the study period were dominated by *Aphanizomenon flos‐aquae* and occurred in the lower SLRE at Barker's Island in late summer 2023 when conditions were N‐limited but not N‐depleted. This result aligns with past research indicating that diazotrophic cyanobacterial abundance more strongly correlates with nitrate than non‐diazotrophic species and non‐cyanobacteria phytoplankton (Donald et al., 2011). This also aligns with established ecological theory that suggests diazotrophic taxa gain a competitive advantage and establish dominance under N‐limited (but not necessarily N‐depleted) conditions (Schindler, 1977; Smith, 1983). Donald et al. (2011) also found a stronger link between diatoms and nitrate than NH_4_ and a stronger link between non‐diazotrophic cyanobacteria and NH_4_ than nitrate; water quality dynamics that align with our observations of higher diatom abundances in the lower SLRE throughout 2023 and higher abundances of *Microcystis aeruginosa* in 2024. In laboratory studies, isolated strains of both *M. aeruginosa* (Finlay et al., 2010) and *Dolichospermum* spp. (Kramer et al., 2022) were found to increase growth with NH_4_ enrichment, consistent with the observations here. The benthic taxon *Oscillatoria* sp., identified in the SLRE in this study, is used in wastewater treatment for its high ammonium affinity (Hashimoto & Furukawa, 1989), potentially helping to explain its most intense occurrences at Miller Creek near municipal treated effluent discharge. Although phosphorus concentration has been demonstrated as a driver of cHABs and cultural eutrophication (Correll, 1998; Schindler, 1974), TP and SRP had very low predictive power in our study. This likely reflects the consistently high (non‐limiting) TP and SRP concentrations (typically >30 μg · L^−1^) in the SLRE that support year‐round algal growth and metabolism. Instead, several nitrogen forms strongly predicted cyanobacterial biovolume, all with negative marginal effects. This relationship is likely driven by the seasonal peak of cyanobacteria with N‐limited baseflow conditions in the late summer, most intensely and prolongedly following 2023 drought conditions.

The highly correlated variables TOC and DOC were important predictors of cyanobacteria and phytoplankton overall. Although carbon can have direct impacts on cyanobacteria physiology (Klemer et al., 1996), the 2023 drought resulted in low TOC and DOC levels, which spatiotemporally coincided with peak cyanobacterial abundance. The strong covariability of phytoplankton communities with carbon likely reflects base flow periods, water residence times, and Lake Superior water intrusions into the lower estuary, all of which affect multiple environmental variables. Baseflow conditions are typical from late July through September but decrease even more in summers with droughts. These conditions also generally coincide with peak seasonal warming, which promotes high productivity and nitrogen (especially NH_4_) drawdown. This convergence of conditions likely explains the strength of the relationship between carbon and phytoplankton.

Acid‐neutralizing capacity, like DOC and TOC, was influenced by hydrologic conditions. The increasing ANC under summer baseflow conditions may be connected to increases in dissolved inorganic carbon (DIC) as the proportion of groundwater rich in HCO_3_‐ and other ions increases in the system (Mosley, 2015; Sprague, 2005; Zieliński et al., 2009) and CO_2_ accumulates via atmospheric dissolution (Liu et al., 2010) with increased water residence time. The proportion of Lake Superior water with low alkalinity relative to SLRE water (Kittaka, 2023; Minor & Brinkley, 2022) likely also increases within the lower SLRE at baseflow as upstream river discharge decreases and seiche‐driven periodic flow reversal influx from the adjacent downstream lake continues. The decline in DOC observed during the 2023 summer drought indicated a decline in concentrations of organic acids, which reduce buffering capacity (Munson & Gherini, 1993), and may further facilitate elevated ANC. In eutrophic systems, photosynthesis can lead to complete drawdown of CO_2_ and shift systems to seasonal carbonate limitation (Butler, 2019), a scenario which may favor cyanobacteria species possessing unique carbon‐concentrating mechanisms (Morales‐Williams et al., 2017; Price et al., 2008). Under such conditions, phytoplankton will use more energetically expensive carbon sources such as HCO_3_^−^ (Talling, 1976), but in the mesotrophic SLRE, CO_2_ was above atmospheric concentrations in almost all samples, suggesting that phytoplankton likely did not play a strong role in ANC by depleting non‐CO_2_ forms of carbon. Future research is needed to understand the seasonal and interannual variability of inorganic carbon flux in the SLRE, particularly if blooms eventually deplete CO_2_.

The importance of temperature for predicting cyanobacterial biovolume and the positive temperature associations of most bloom‐forming cyanobacteria was demonstrated by blooms occurring during and in the weeks immediately following the annual thermal maximum in the SLRE. All recent years in which blooms have occurred in the SLRE (2018, 2021, 2023, 2024) were identified to have anomalously warm late summer water temperatures at Barker's Island, where the blooms were spatially focused (Figure S8; Nicklay, Filstrup, et al., 2025). A direct positive relationship between temperature and cyanobacteria has been well established in natural systems (Kosten et al., 2012; Nalley et al., 2018; Paerl & Huisman, 2008; Rigosi et al., 2014). Top‐down and indirect links have also been identified; zooplankton grazing can be strongly influenced by temperature and productivity (Lewandowska et al., 2014; Mallin & Paerl, 1994) and may exert indirect bottom‐up (i.e., altered aquatic nutrient conditions) or direct top‐down (i.e., selective grazing) control on phytoplankton community structure. Additionally, higher temperatures can increase decomposition rates in estuaries (Bianchi, 2007), which may alter nutrient availability and stoichiometry. *Microcystis aeruginosa* displayed the strongest positive relationship with temperature of any cyanobacteria, a result consistent with the established high thermal requirements of the species (Imamura, 1981; Krüger & Eloff, 1978; Nicklisch & Kohl, 1983; Robarts & Zohary, 1987) and the higher prevalence of the species in 2024, when late summer monthly average water temperatures were higher than in 2023 (Figures S2 and S8). The associations between temperate and other bloom‐forming cyanobacteria including *Aphanizomenon flos‐aquae* were likely primarily due to their peak abundances during the annual thermal maximum. *Aphanizomenon flos‐aquae* is capable of growth and N_2_‐fixation at relatively low temperatures compared to other cyanobacteria (Svedén et al., 2015) and was present in samples months before dominating late‐summer blooms in 2023. Thus, we predict this species may be favored during cooler summers. Although some known cool‐adapted cyanobacterial taxa did display negative relationships with temperature (e.g., *Planktothrix* sp. cf. *agardhii/rubescens*; Ahlgren, 1978; Post et al., 1985), and nearby Lake Superior *Dolichospermum* strains are known to be cold‐tolerant bloom formers (Reinl et al., 2023; Sterner et al., 2020), species with negative temperature associations were a minor component of the total cyanobacterial biovolume in our study and never dominated. Thus, we predict that future years with warmer‐than‐average summer months will carry higher risk of SLRE blooms, and relative thermal optima among species may play a role in determining which species will dominate.

Variables associated with light availability (PAR_kd_, Secchi, turbidity, TSS, VSS) had moderate to low importance across most RFA models, despite the role of light as a driver of phytoplankton dynamics (Filstrup et al., 2016). Relationships with PAR_kd_ in the upper‐middle SLRE may relate to the persistently DOC‐stained nature of the region limiting light availability; cyanobacterial biovolume peaked when DOC was lowest and PAR_kd_ was highest in late summer 2023, supporting this interpretation. The low importance of light‐associated variables in the lower SLRE may pertain to persistent resuspension of phytoplankton in this shallow polymictic system that minimizes light limitation for plankton except at times of exceptionally high turbidity, such as spring discharge (Schallenberg & Burns, 2004). While total suspended solids influence light availability, this parameter is also partly comprised of phytoplankton biomass, a redundancy that may explain the high predictive importance of TSS relative to other light‐associated variables. Volatile suspended solids were consistently low or non‐detectable, and thus susceptible to error. The negative association of PAR_kd_ with predicted cyanobacteria (RFA) and the positive association of Secchi with bloom‐forming cyanobacterial species (CCA) indicated peak cyanobacteria at times of highest water clarity, but overall, a moderate to weak relationship with light‐related variables indicates nutrients and temperature likely play more critical roles in bloom dynamics. However, the potential secondary or tertiary role of light in shaping phytoplankton communities and the known ability of certain bloom‐forming cyanobacteria to gain a light availability advantage through intracellular buoyancy regulation (Brookes & Ganf, 2001; Reynolds et al., 1987) are consistent with the direction of the relationships we observed.

The high importance of DO and pH as predictors of cyanobacteria in the lower SLRE likely relates to photosynthetic algal production of DO and depletion of CO_2_ (Smith & Piedrahita, 1988; Talling, 1976). Strong seasonal covariability of DO and pH across sites likely also influenced statistical relationships, evidenced by the alignment of winter taxa with maximum DO and late‐summer bloom‐forming taxa with maximum pH. Although winter hypoxia did occur ephemerally in conjunction with slightly acidic pH at the turbid Allouez Bay and Pokegama Bay sites, it was rare, indicating that winter internal loading associated with hypoxia‐induced redox reductions (Nürnberg, 1984) is likely a minimal or temporally inconsistent factor in SLRE bloom dynamics. Furthermore, with a short water residence time (Bellinger et al., 2016), any nutrients loaded internally in the winter may be exported from the system months before bloom onset. However, winter diatom blooms of *Aulacoseira ambigua* and *Au. granulata* with abundant reproductive auxospores indicate ongoing proliferation in Allouez Bay, suggesting short‐term dynamics may connect hypoxia to winter phytoplankton.

#### Context and comparisons

Our findings were consistent with results from paleolimnological data on fossil pigments, inferred nutrients at North Bay, Pokegama Bay, Billings Park, and Allouez Bay, and cyanobacterial pigments at North Bay and Billings Park (Alexson et al., 2018). Combined, these data suggest that recent high abundances of cyanobacteria are a newly emerging problem since the 1980s, particularly in nearshore (non‐thalweg) areas. Several of the high‐nutrient diatom indicators previously identified as increasing at SLRE locations (Alexson et al., 2018) were also documented as abundant in the SLRE in our study. For example, paleolimnological and water quality observations (Alexson et al., 2018; Bellinger et al., 2016) clearly depict the SLRE's lower food web as a changing, phosphorus‐driven system throughout most of the 20th century. Current water quality and phytoplankton dynamics are the product of a nutrient‐enriched and urbanized system, and our new observations indicate that the structure and dynamics of the SLRE's lower food web are now determined to some degree by N‐limitation. Seasonal community progression observed here in the SLRE was notably distinct from classic lacustrine models (Sommer et al., 1986, 2012) and from locations around the Great Lakes (Reavie, Barbiero, et al., 2014). Commonly, there is a clear temporal separation in dominant algal phyla throughout a given year, but unlike typical lake systems, in the SLRE abundant diatoms and other siliceous algae coincided with cHABs.

#### Lag analysis

Previous studies (e.g., Liu et al., 2022) suggested that cHABs may respond to environmental triggers with a time delay. For example, a storm‐driven nutrient pulse may lead to a bloom several days or weeks later, as was suggested for southern Lake Superior (Sterner et al., 2020). As such, the lag analysis explored whether certain conditions captured through routine monitoring were correlated with high cyanobacterial biovolume observed later. The increased correlation between cyanobacteria and temperature at a 30–40 day lag, and NH_4_, TN:TP, and PAR_kd_ at an ~4–10 day lag, indicates that the weeks or months preceding blooms in the SLRE may contain ecologically relevant, predictive information. The time delay for temperature, along with its importance as a same‐day predictor in RFA, is consistent with prior research (e.g., Robarts & Zohary, 1987; Wagner & Adrian, 2009). Despite the plentiful direct links established between temperature and cyanobacteria growth (Huisman et al., 2018), the indirect effects of temperature which may contribute to the time delay effect (e.g., stratification duration, Schmidt stability) may play stronger roles in facilitating cyanobacteria dominance within community structures regardless of absolute cyanobacterial concentrations. The absence of stratification at our shallow, well‐mixed sites indicates that some other factor than these specific mechanisms may indirectly link early summer temperatures with late‐summer cyanobacteria. An expanded long‐term phytoplankton community dataset could facilitate a detailed investigation of links between seasonal temperature patterns and phytoplankton community structure progression in the SLRE to develop mechanistic hypotheses. Although our approach does not yet offer actionable early‐warning thresholds, these findings highlight the potential for using early summer observations, particularly temperature and signs of N‐limitation, to anticipate bloom risk later in the season.

### What environmental variables should be monitored?

#### Environmental variable selection

The above ecological interpretation of our results, along with consideration of the cost and effort associated with environmental measurement, informs our recommendations for variables to include in future monitoring (Table 6; described in greater detail in an ancillary technical report: Nicklay, Filstrup, et al., 2025).

**TABLE 6:** Considerations for incorporating study variables into a future monitoring strategy.

Environmental variable	Is it needed? Why or why not?
Temperature	Yes. Important; low‐cost; continuous in situ preferred.
Dissolved oxygen	Yes. Low‐cost; continuous in situ preferred; bloom proxy.
pH	Yes. Low‐cost; continuous in situ preferred; bloom proxy.
Specific conductivity	Yes. Weak predictor of blooms, but useful proxy of drought conditions and pollution.
Salinity	No. Not critical, can be calculated from spcond.
Secchi depth	Yes. An important low‐cost surrogate for turbidity.
Chlorophyll *a*	Yes. Common measure of productivity; comparison to other systems; in situ chl *a* fluorescence preferred for real‐time alerts; discrete chl *a* preferred for continued long‐term tracking.
Total phosphorus	Yes. Not a strong predictor of cyanobacteria, but useful for TN:TP calculation, long‐term change tracking, and comparisons to other systems.
Soluble reactive phosphorus	No. Not a strong predictor of cyanobacteria; consistently available; relatively high‐cost variable.
Total nitrogen	Yes. Impacted by drought; spatiotemporally variable; important for predicting cyanobacteria; implications for dominant bloom species.
Ammonium	Yes. Impacted by drought; high spatial heterogeneity; important for predicting cyanobacteria; implications for dominant bloom species.
Nitrates + nitrites	Yes. An important predictor of cyanobacteria especially in the middle and upper estuary; implications for phytoplankton communities.
TN:TP ratio	Yes. Calculated ratio of two already‐included variables; influenced by drought; moderate predictive importance for cyanobacterial biovolume; implications for dominant bloom species.
Acid‐neutralizing capacity/alkalinity	No. Redundant with other variables; requires near‐immediate laboratory analysis.
Dissolved organic carbon	Yes. High spatial, seasonal, and interannual variability; useful drought condition proxy; implications for water clarity.
Total organic carbon	No. TOC is highly correlated with DOC.
Carbon dioxide	No. Not depleted during most intense past blooms; long sensor equilibration time; iteratively reconsider inclusion.
Turbidity	Maybe. Secchi depth is an excellent lower cost surrogate.
Total suspended solids	Yes; but only at SWMP sites to continue tracking of loads and long‐term trends.
Photosynthetically active radiation	No. Related to turbidity and Secchi depth, at higher cost and required time to measure.

Given the high importance of nitrogen forms among nutrients and the observed links between drought, nitrogen depletion, and cHABs, we recommend continued inclusion of all total and dissolved nitrogen forms that we measured in future monitoring and research in the SLRE. Although we did not measure dissolved organic nitrogen, it is influenced by rainfall and drought and has been shown to play a role in phytoplankton community composition (Seitzinger & Sanders, 1999), and future study or monitoring could examine its ecological role in the SLRE. Despite the limited statistical importance of phosphorus for predicting cyanobacteria due to its persistent availability, we recommend continued monitoring of TP, a ubiquitous monitoring focus, for continued long‐term change tracking and comparisons to other systems; however, to minimize unnecessary costs, we recommend omission of SRP going forward in the short term unless future results point toward an increasing role of SRP in contributing to cHAB dynamics. Although we did not measure silica, it can influence phytoplankton community structure (Martin‐Jézéquel et al., 2000) and is vital for the growth of diatoms, the dominant group in the SLRE. However, previous research indicated that silica remains elevated year round in the SLRE and does not experience drawdown during spring diatom growth (Johnson & Eisenreich, 1979), suggesting that it is unlikely to limit phytoplankton growth in the system.

Given warming trends, the timing of past blooms in late summer when water temperatures were near annual maxima, and the occurrence of all past cHABs in anomalously warm late summer months in the SLRE, year‐round temperature monitoring is critical. Continued warming may facilitate further taxonomic shifts and exacerbate blooms, and understanding these dynamics as they unfold in real time will be of paramount importance for studying future cHABs and protecting the public. Climate‐change‐associated changes in temperature and productivity may be altering DO dynamics in the SLRE, as they have in lakes throughout the temperate region globally (Jane et al., 2021). Given the utility of DO and pH as real‐time indicators of blooms, their importance in understanding winter dynamics, their susceptibility to long‐term change, and their low monitoring cost, we recommend inclusion of both variables in all future monitoring. The general lack of information on winter nutrient cycling and primary producer dynamics warrants further study. Discrete winter sampling events did not capture DO variability or short‐term hypoxia events as effectively as in situ loggers (Nicklay, Filstrup, et al., 2025). Although discrete winter sampling of DO relies on safe ice conditions and requires significant effort for each measurement to be obtained, moored in situ loggers collect readings at a much higher frequency, reduce field effort with only an initial deployment and subsequent pickup several months later, and have low ongoing maintenance costs (i.e., biannual sensor cap replacements). Hence, we recommend continued deployment of this technology combined with targeted analysis of winter water quality and phytoplankton communities.

Despite the potentially low direct effects of carbon on cHABs and other phytoplankton dynamics (such as the occurrence of mixotrophic taxa such as cryptophytes and dinoflagellates; Jansson et al., 1996, Hansen & Calado, 1999), the management utility of the variable as a drought proxy and as a metric of long‐term change warrants its inclusion in future monitoring. We recommend that DOC is needed going forward, but not TOC, given the high redundancy of the two variables, the cost associated with measurement, and the likelihood that dissolved forms of carbon are likely to have more direct effects on phytoplankton. For now, considering the long CO_2_‐sensor equilibration time, the short time window required for ANC sample processing after collection, and the relatively low amount of cyanobacterial variance explained by organic and inorganic carbon and ANC, we do not recommend ANC and CO_2_ for inclusion in future routine sampling. A strong relationship between ANC and spcond (Figure S1) suggests the potential to model ANC from the lower cost proxy in future monitoring. We recommend inclusion of a low‐cost, light‐associated variable such as Secchi in future monitoring. Secchi is a common proxy for light availability, and inclusion of Secchi and omission of most other light associated variables in future SLRE monitoring would facilitate future tracking of changes in light and its role in bloom dynamics and provide a standardized mode of comparison to other systems (e.g., Secchi‐derived trophic state index; Carlson, 1977).

Going forward we recommend continued full taxonomic enumeration at 400× magnification, if logistically and financially feasible, to continue to capture the taxonomic composition of phytoplankton communities. Enumeration at 400× magnification is sufficient for identification of the most common year‐round and bloom community members (e.g., *Aphanizomenon flos‐aquae, Aulacoseira ambigua, Rhodomonas minuta*) comprising the majority of sample biovolume, but a future study could incorporate higher magnification microscopy to identify picoplankton taxa and useful diatom indicators with small cell sizes. Changing environmental conditions may facilitate future dominance of sparse or ephemeral species, a phenomenon that has been observed with nuisance algae such as *Didymosphenia* (Taylor & Bothwell, 2014). More generally, knowing the taxa and their ecologies provides important information that is missing in bulk measurements of phytoplankton abundance, such as Chl *a*. If funding availability is limited, Chl *a* does provide a useful proxy for analyses of bloom predictors, given its significant relationship with cyanobacterial biovolume in the SLRE. However, at lower SLRE sites, the observation that our simple regression model tended to underpredict cyanobacterial biovolume at the highest ranges of Chl *a* (Figure S3; upper right) serves as further evidence of the importance of taxonomic enumeration for tracking cyanobacterial abundance and trends. In addition to phytoplankton, future study of SLRE zooplankton communities could provide valuable insights regarding top‐down controls on phytoplankton communities. Molecular approaches to phytoplankton quantification may be explored in the future to gain taxonomic insight and study the roles of functional genes in mechanisms associated with bloom onset. We strongly advocate for the inclusion of toxins in future monitoring, particularly for bloom response samples, to better understand the drivers of toxin production, parse out toxicity‐associated relationships for species, and alert the public of health risks. This is particularly important at high‐use locations with past cHABs, such as the public beach adjacent to our Barker's Island site. In combination with our periodic toxicity testing, the efficacy of low‐cost antigen‐based rapid cyanotoxin test strips was evaluated in the SLRE in 2024 (Widiker & Reinl, 2025), and it was determined that the technology may hold promise for integration in future management in conjunction with standard laboratory‐based toxin quantification methods.

### What temporal resolution should be used?

Temporal redundancy analyses did not identify a single, optimal sampling frequency for future monitoring because environmental variables had wide temporal variability across sites and lag effects were also variable or non‐existent (Table 7). Thus, a more site‐tailored, judgment‐based approach is recommended.

**TABLE 7:** A proposed, three‐tiered approach to sampling frequency at SLRE locations identified in Table 8.

Sampling frequency tier	Description
**Tier 1**. Year‐round sampling	Monthly sampling at all stations, including recommended water quality (Table 6) and preserved phytoplankton collections (can be archived or analyzed immediately if funds allow).
**Tier 2**. Higher resolution sampling during ice‐free periods	Sample as for Tier 1, but increase sampling frequency to biweekly at a subset of locations that represent SLRE conditions (Miller Creek, Barker's Island, Allouez Bay, Pokegama Bay). Continue monthly sampling at remaining locations.
**Tier 3**. cHABs‐focused and bloom‐response sampling	Starting in late July every year, increase intensity of sampling to weekly at bloom‐susceptible locations through October; particularly Barker's Island. If blooms are spotted, immediate response sampling should include surface scum and toxin analysis, using rapid‐assessment procedures; samples with detectable toxins should be analyzed quantitatively by laboratory methods.

#### Temporal redundancy

Variables such as temperature showed continuous improvement in data quality with more frequent sampling, whereas others, such as phytoplankton biovolume, had such high temporal variability that an ideal, system‐wide sampling frequency could not be clearly identified. We attribute this to high spatiotemporal, hydrologic complexity in the SLRE and advise that similar investigations in more stable, lacustrine systems might provide different results. In general, higher sampling frequencies were non‐asymptotic relative to variability of environmental parameters. Some locations consistently showed higher biologically driven variability across all time intervals. For example, Allouez Bay, Barker's Island, and Miller Creek had higher overall Chl *a* concentrations and exhibited greater differences between sampling events, regardless of whether those events were separated by days or weeks. This suggests that sites with higher baseline concentrations also tend to have greater short‐term fluctuations. Hence, sites with high phytoplankton biomass, such as those in the lower harbor, showed greater variability in Chl *a* and cyanobacterial biovolume, indicating that higher frequency sampling is warranted to capture productivity dynamics. Although our statistical approach has not been widely applied in estuarine cHAB research, our findings are consistent with results from researchers who applied similar methods in coastal South Australia, where daily sampling was determined to be preferable to weekly sampling for capturing Chl *a* variability in monitoring data (Elsdon & Connell, 2009). Timing is parameter specific; for example, if monitoring efforts are focused on regulatory environmental variables like phosphorus, more frequent sampling may be more valuable at sites such as Pokegama Bay where TP variability was more pronounced.

#### Barker's Island case study

The sequentially decreasing RFA model performance in the temporal redundancy analysis of the Barker's Island dataset (weekly, biweekly, monthly) was consistent with other studies which had similar results for high‐resolution sensor data (Derot et al., 2020). The shuffling of predictor importance rankings associated with environmental variables across temporal subset models (Table 3) is likely caused by the loss of resolution around acute bloom periods (cyanobacteria peaks), with lower temporal resolution favoring RFA selection of variables with longer term, seasonal cycles, such as DO. However, marginal effects of environmental predictors on cyanobacteria remained consistent across subset models, indicating that some qualitative relationships can still be studied at a lower sampling frequency. Nonetheless, for the best understanding of the ecological dynamics driving cHABs, higher sampling frequency in the late summer probably yields the most useful insights.

#### Routine sampling

Given funding limitations, a recommendation to sample “as frequently as possible” is not helpful, so we recommend a tiered approach going forward using existing protocols (Table 7). Monthly samples to characterize SLRE water quality condition would likely suffice at well‐mixed, thalweg locations to address long‐term trends, with more frequent sampling (e.g., weekly) at sites with greater cHABs concerns, particularly in the warm late summer months. If sampling effort is limited, this sampling approach should be prioritized at areas of high public use and with histories of cHABs. Visible surface accumulations (scums; aggregates of planktonic and/or benthic cyanobacteria) at any locations should be visually assessed. This practice increases the likelihood of the sampling design incidentally capturing cHABs if they occur and can also provide an early warning of bloom development and what pre‐bloom taxa are present. Similar temporal frameworks could be applied to establish foundational cHAB monitoring programs in other estuarine, aquatic, and marine systems, and the temporal structures of those programs could be similarly optimized based upon analyses of initial monitoring data.

#### Bloom response sampling

Above all else, we recommend that bloom‐response samples be prioritized above routine samples in the interest of public health, tracking cHABs over time, and refinement of ecological understanding by modeling key environmental relationships. Bloom response sampling requires perpetual readiness of field and lab equipment, a boat, and staff for “grab and go” situations. Real‐time bloom indicators such as elevated pH, fluorometric Chl *a*, and daytime DO saturation can trigger sending a crew to visually survey for blooms in sheltered bays and slips with easy public access, and observation of macroscopic colonies (e.g., *Aphanizomenon flos‐aquae*) or scums should trigger immediate collection of a sample suite, a rapid toxin assessment, and a wider visual survey.

Our study identified early morning to be the most susceptible to scum formation coincident with calm winds that generally increase as a day progresses. When winds increase, scums disperse into the water column; however, this likely does not reflect any reduction in cyanobacterial biovolumes or toxin concentrations, particularly in the shallow SLRE. This should be considered in the context of public cyanotoxin exposure risk at shallow swimming areas in all cHAB‐prone systems.

#### Storm sampling

Given the association between storms and cyanobacteria blooms in southern Lake Superior (Sterner et al., 2020), a planned strategy was to conduct targeted storm response sampling in this region. This was not necessary during our study because routine sampling aligned closely with rain events. We did not identify any direct responses of phytoplankton to storm‐induced shifts in conditions in the days or weeks immediately following storms, but as discussed above, storms and drought likely mediated relevant nutrient conditions—for example, the SLRE‐wide increases in TN and TN:TP following precipitation that terminated a prolonged drought in late September 2023. Sampling at times of peak discharge, including spring melt and immediately following storms, is useful for tracking nutrient loads and long‐term changes at main‐thalweg locations (Nicklay, Filstrup, et al., 2025), but coincident phytoplankton samples are often turbid, diluted, and laborious to enumerate, making them of limited value. These challenges and the lack of predictable responses of phytoplankton to storms justify omission of storm response sampling from future SLRE monitoring design, but the impacts of storm and drought regimes should be strongly considered as context around summer nutrient conditions related to phytoplankton communities both here and in similar systems.

### What locations should be monitored?

We aimed to identify a subset of sampling locations that will comprehensively provide a set of monitoring data that spatially describe SLRE conditions (Table 8) and serve as a model for designing future monitoring efforts. This selection involved eliminating redundancy among sites, considering stations with existing long‐term data, and addressing spatial concerns related to cHABs. Our statistical approach may be useful in other monitoring programs seeking to optimize spatial effort, though our interpretation focuses primarily on cHAB monitoring in the spatial context of the SLRE.

**TABLE 8:** Considerations for incorporating study sites into a future monitoring strategy, including preliminary prioritization based on ecological value, bloom history, logistical feasibility, and spatial coverage.

Where	Needed? Prioritized? Why/why not?
North Bay	Yes. Low Priority. Although the upper estuary in general has low bloom‐susceptibility, winter hypoxia, and climate warming trends warrant tracking a sheltered bay in the upper estuary long‐term.
Mud Lake	No. Mud Lake was generally redundant with North Bay and similar to the SWMP Oliver Bridge station. It is not particularly susceptible to blooms. While winter hypoxia did occur it was more severe in North Bay.
Pokegama Bay	Yes. High Priority. As a SWMP station with persistent summer and winter hypoxia, it is a high priority to maintain monitoring at this site. Although current conditions do not foster blooms, nutrients are high and could contribute to blooms downstream.
Kingsbury Bay	Maybe. Low Priority. In general, the Kingsbury Bay site was redundant with Billings Park and did not appear to be of concern for algal blooms. Based on future priorities and any expanded recreational access, Kingsbury Bay or Billings Park could be selected.
Billings Park	Maybe. Low Priority. In general the Billings Park site was redundant with Kingsbury Bay and did not appear to be of concern for algal blooms. Based on future priorities and any expanded recreational access, Kingsbury Bay or Billings Park could be selected.
Miller Creek	Yes. High Priority. This site is prone to blooms and has particularly high ammonium concentrations, giving it a unique characteristic for the SLRE. While SWMP's Blatnik Bridge station is close by, the unique water quality of the Bay where Miller Creek enters the estuary is distinct.
Barker's Island	Yes. High Priority. It is a very bloom prone site, has an existing SWMP station, and is proximal to a public swimming beach, therefore is a high priority to maintain. While SWMP collects monthly nutrients samples, we recommend high frequency routine sampling in summer and fall to act a bloom sentinel for the lower estuary.
Allouez Bay	Yes. High Priority. The bay has distinct water quality, winter hypoxia, and unique winter phytoplankton communities. While no blooms extended into Allouez Bay during the study period, the site had relatively high phytoplankton and cyanobacterial biovolume.

#### Cyanobacteria bloom locations

The occurrence of summer cyanobacteria blooms in the two study years substantially improved our ability to characterize likely locations of future cHABs, and bloom response sampling at additional locations enabled characterization of greater spatial variability. Blooms are occurring in SLRE locales beyond those we sampled, but the consistency in timing for phytoplankton community dynamics among sites (especially in the lower estuary) suggests our recommended sites provide good sentries for tracking and prediction of blooms. Barker's Island appears to be the most critical site for bloom monitoring, as it experienced the highest frequency and intensity of blooms during our project, was the only site noted to have experienced prior blooms (Lafrancois & Reinl, 2025), and had a 13‐year record of water quality and nutrient data. When blooms were observed anywhere in the SLRE, they almost always occurred simultaneously at Barker's Island. Iterative reevaluation of monitoring should consider the potential inclusion of new locations, such as the mouth of Pokegama Bay where only one minor bloom (with unique community structure) occurred, assuming blooms may become more commonplace at non‐monitored locations.

#### Spatial heterogeneity

Optimal spatial coverage for monitoring varies depending upon whether the goal is to capture uniqueness and minimize redundancy in water quality, phytoplankton communities, or both. Because phytoplankton communities were more temporally stable than water quality, sampling frequency optimization was focused on capturing water quality variability. Such a monitoring program will inherently capture phytoplankton community variability. However, phytoplankton enumeration is a time‐intensive process and can only be performed by trained taxonomists. We recommend collecting phytoplankton samples with all sample suites regardless of the short‐term ability to perform enumeration, as phytoplankton sample collection is inexpensive and preserved samples can be archived prior to enumeration.

The difference in spatial redundancy between water quality and phytoplankton likely reflects the rapid and variable nature of water quality conditions, which can fluctuate on short timescales, whereas phytoplankton communities tend to respond more slowly, with many taxa having broad environmental tolerances (Reavie, Heathcote, & Shaw Chraïbi, 2014). As an example, water quality at Pokegama Bay is distinct from surrounding areas, but phytoplankton communities are similar, with the presence of cosmopolitan taxa such as the common *Rhodomonas minuta* (Lund, 1962), which can grow in a broad range of conditions. Higher spatial differentiation of water quality and phytoplankton communities in the summer (vs. year round) indicates that a greater number of locations is required to capture spatial heterogeneity at the time of year coincident with blooms. At other times of year, less frequent monitoring may suffice to capture spatial variability. The greater differentiation in the summer may be connected to slowing hydrology, with sites becoming less hydrologically connected to one another, setting up localized nutrient limitation, and creating more niches for phytoplankton community development. Based on the spatial redundancy analysis of water quality data, we expect that additional unique, localized coastal sites (bays, tributaries) could be identified within the SLRE, and we cannot rule out revision of the recommended sampling network based on future observations. Future research could expand on our work by evaluating spatial redundancy in cyanobacteria–environment relationships (e.g., Zhang et al., 2021), which would be especially useful if future blooms temporally coincide in different regions of the SLRE but have distinct water quality conditions and dominant cyanobacteria.

## CONCLUSIONS

A thorough, 2‐year assessment of water quality and phytoplankton communities was carried out in the SLRE. The findings suggest that if external, anthropogenic nutrient inputs remain unchanged, then future bloom dynamics in the SLRE will likely be shaped by climate driven shifts in temperature and hydrology. Denitrification‐induced N‐limitation and NH_4_ depletion, in conjunction with readily available phosphorus, appear to be shifting conditions to a more favorable state for diazotrophic cyanobacteria, especially in the lower SLRE. Additionally, this investigation identified predictors and likely drivers of phytoplankton dynamics and provides a recommended structure for a future monitoring program, with variations depending on funding availability and priorities associated with cHABs and water quality. There is a need to continue monitoring for increased algal abundance and shifting nutrient dynamics within the system, as the effects of global climate trends have likely played a strong role to date, and may continue to do so, in altering the frequency, severity, taxonomy, and toxicity of cHABs in the SLRE. Our findings on stoichiometric drivers of cHABs, and our methodological approach to the development of a monitoring program, could be applied elsewhere (e.g., impacted Great Lakes bays and estuaries, coastal marine systems, lakes) to identify drivers of unexplained ecological shifts and support effective monitoring programs.

## AUTHOR CONTRIBUTIONS

**Peter A. Birschbach:** Data curation (equal); formal analysis (lead); investigation (equal); methodology (supporting); visualization (lead); writing – original draft (lead). **Euan D. Reavie:** Conceptualization (equal); funding acquisition (equal); methodology (equal); project administration (lead); supervision (lead); writing – review and editing (equal). **Christopher T. Filstrup:** Conceptualization (equal); funding acquisition (equal); investigation (equal); methodology (equal); writing – review and editing (equal). **Hannah Nicklay:** Conceptualization (equal); data curation (equal); funding acquisition (equal); investigation (equal); methodology (equal); project administration (lead); resources (lead); supervision (lead); writing – review and editing (equal).

## Supporting information


**Figure S1.** A Kendall rank correlation matrix using observations from the eight sampling sites. Scatterplots are in the lower triangle and annotated correlation coefficients with *p*‐values are in the upper triangle. Statistically significant (*p* < 0.05) positive correlations are dark red and negative correlations are navy blue. Non‐significant correlations are black. Variable names corresponding to the abbreviations in the matrix can be found in Table 2.

**Figure S2.** Time‐series plots of water quality and nutrient variables collected from the eight project sampling locations, 2023–2024. Plots include points representing individual observations and LOESS curves (*span* = 0.1) fit to individual sites and the whole SLRE; due to intermittent CO_2_ observations, a scatterplot was used for CO_2_. Site names corresponding to codes are in Table 1, and variable names corresponding to the abbreviated names on plots can be found in Table 2.

**Figure S3.** Scatterplot of log_10_(chl *a*) versus log_10_(cyanobacterial biovolume) with a superimposed line of fit and shaded error range from simple linear regression (*m* = 0.76, *b* = 3.90, *F*(1, 270) = 65.11, *R* ^2^ = 0.19, *p* = 2.34e‐14). Point color corresponds to site, while point size progressively increases for each month of the year. Site names corresponding to codes are in Table 1. While significant, this regression illustrates the relatively weak relationship between morphological and pigment measurements as surrogates for phytoplankton abundance.

**Figure S4.** Cyanobacteria community composition for each bloom response sample. Pie charts display each cyanobacteria species biovolume as a proportion of total cyanobacterial biovolume (μm^3^ · mL^−1^). Note that species with <1% in any given chart are not visible but are listed in the legend. They are organized from top to bottom by date with total cyanobacterial biovolume for each sample displayed in the center of each pie.

**Figure S5.** CCA species scores, as in Figure 5 (right), plotted as species codes in lieu of points. Species code label centroids are located at point coordinate locations. Taxa associated with each code are listed in Table S3.

**Figure S6.** Lag analysis of the relationships between selected environmental variables and phytoplankton variables, or proxies, including combined phytoplankton, cyanobacteria, or diatom biovolume and Chl *a*. Lags in environmental data measurements from 0 to 50 days prior to phytoplankton measurements were explored using the absolute value of the correlation coefficient between the variable and biovolume.

**Figure S7.** Visualizations of axis 3 for NMDS analysis that was used to assess spatial redundancy in water quality and phytoplankton communities (Figure 8).

**Figure S8.** Monthly water temperature (°C) anomalies, calculated as each month's deviation from historic monthly mean (2013–2024). Data from LSNERR SWMP continuous monitoring station at Barker's Island (Office of Coastal Management, 2025). http://www.nerrsdata.org; doi:10.25921/vw8a‐8031

**Table S1.** Summary water quality data for the project sample locations, for the sample period (October 2022 to November 2024), not including winter collections. Empty cells indicate no data collected for that parameter. Water quality variable names corresponding with abbreviations are included in Table 2. Although summary data exclude winter collections, the total number of routine and bloom response sample sets collected at each site throughout the entire sample period are indicated in the first two rows.
**Table S2.** Cyanotoxin testing results from limited non‐routine sampling efforts in 2023 and 2024. Bloom response samples were tested using Wisconsin DNR‐provided kits and analyzed by the Wisconsin State Lab of Hygiene for total microcystins and saxitoxin. Guidance Thresholds: Drinking Water (EPA, 2019): Microcystin 0.3 μg · L^−1^ (children), 1.6 μg · L^−1^ (adults), no formal threshold for Saxitoxin; Recreational Water (World Health Organization, 2003): Microcystin—8.0 μg · L^−1^; Saxitoxin 10–30 μg · L^−1^. ND = non‐detection.
**Table S3.** All (107) distinct phytoplankton taxa included in the final CCA (*R* ^2^_adj_ = 0.06; chi‐square = 0.36; *df* = 8; *F* = 3.14; *p* = 0.001) with ordination scores for the first three CCA axes and statistics explaining fit of species data to environmental data. For each taxon, *R* ^2^ indicates the fit of the respective taxon to the environmental gradients, and *p* indicates the significance of the fit as determined by permutation testing (999 permutations). Of 107 taxa, 77 responded significantly (*p* < 0.05) along environmental gradients.
