# Supplementary material for: Optimizing a long‐term monitoring strategy to track emerging cyanobacterial blooms in a Great Lakes estuary
Source: J Phycol. 2026 Aug 7;62(4):1264–96. doi: 10.1111/jpy.70215 (PMC13485230; doi:10.1111/jpy.70215)
Supplement: Supplementary file 1 [file JPY-62-1264-s003.pdf]

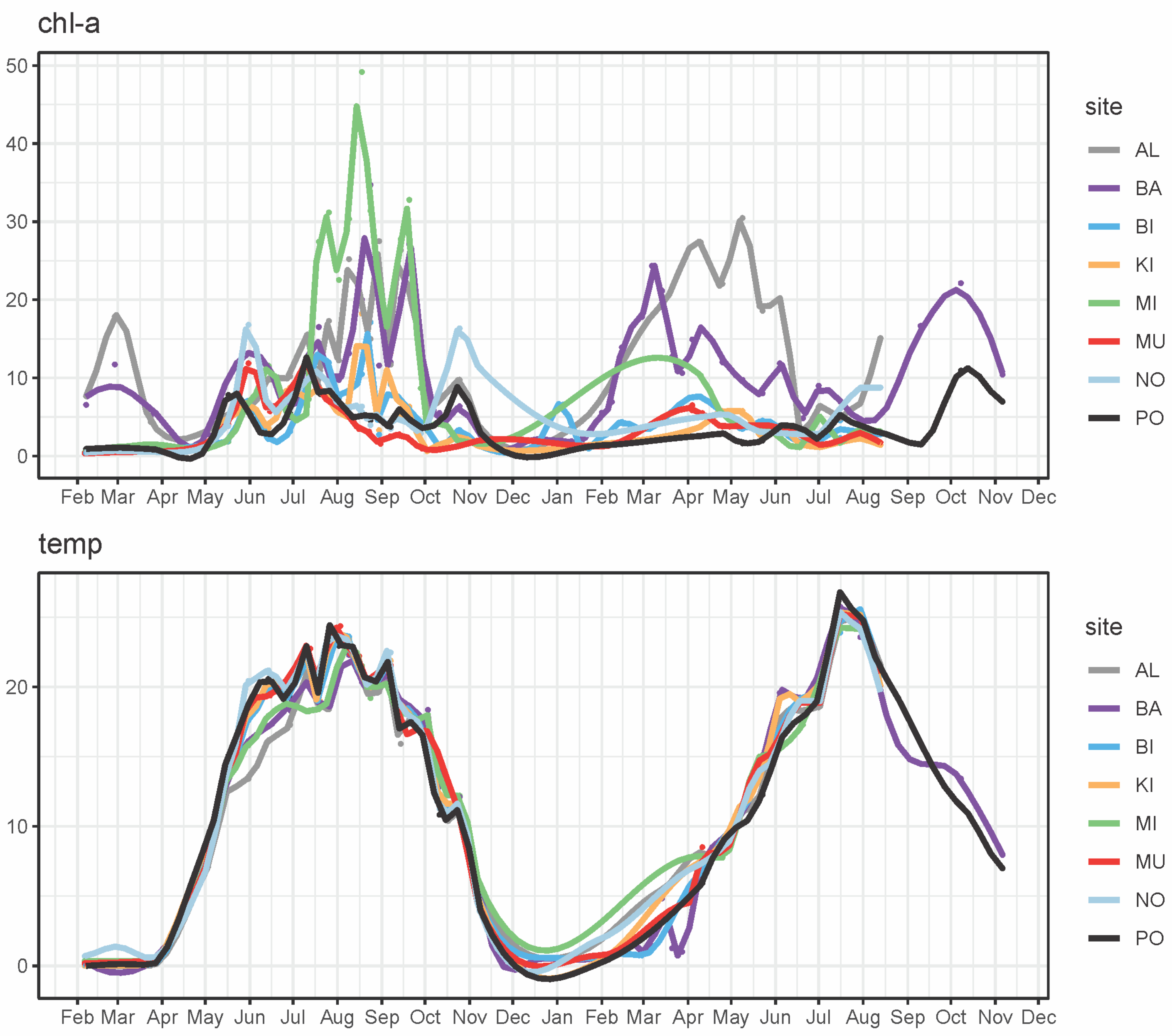
chl-a
50
40
30
20
10
0
Feb Mar Apr May Jun Jul Aug Sep Oct Nov Dec Jan Feb Mar Apr May Jun Jul Aug Sep Oct Nov Dec
site
AL
BA
BI
KI
MI
MU
NO
PO
temp
20
10
0
Feb Mar Apr May Jun Jul Aug Sep Oct Nov Dec Jan Feb Mar Apr May Jun Jul Aug Sep Oct Nov Dec
site
AL
BA
BI
KI
MI
MU
NO
PO

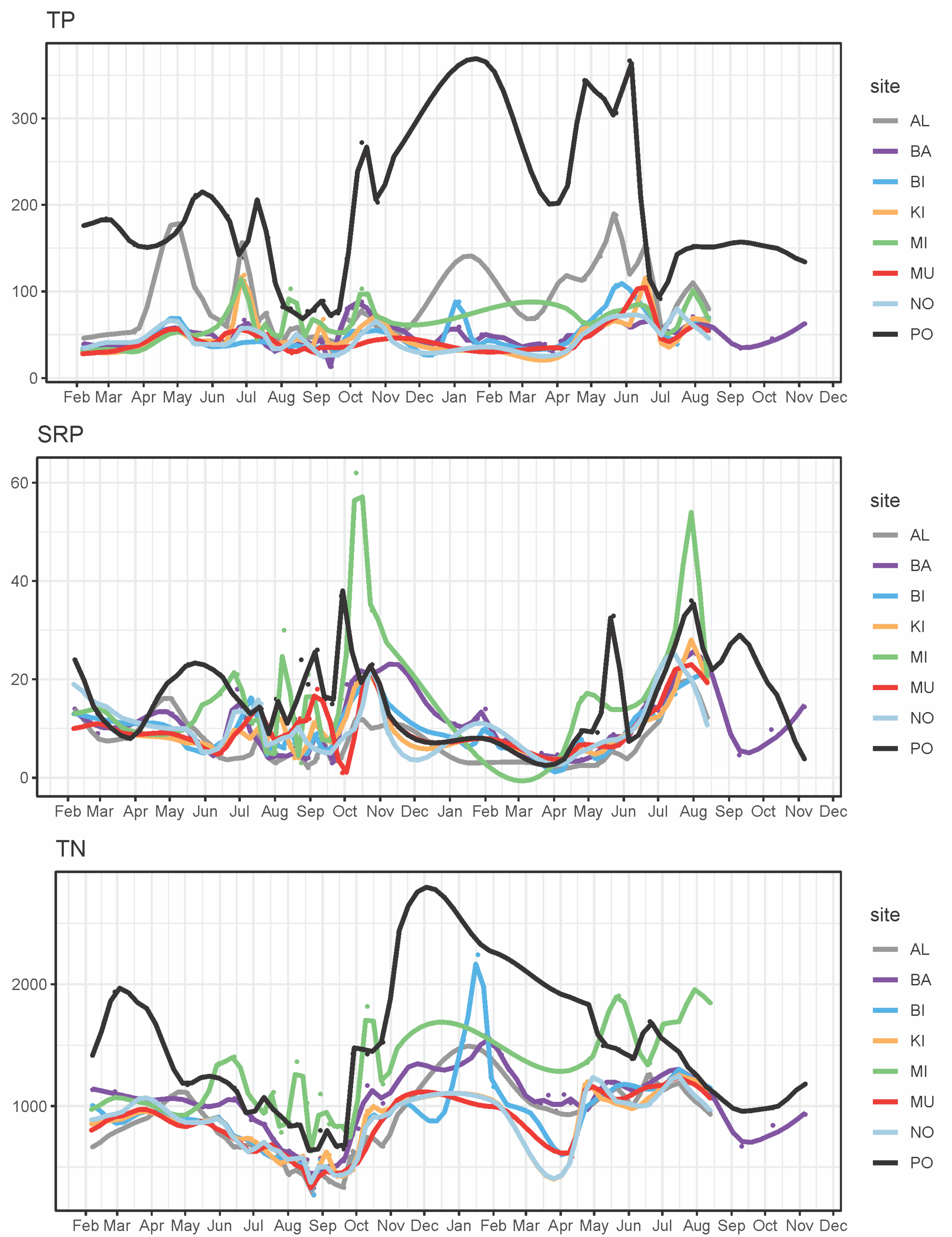
TP
300
200
100
0
Feb Mar Apr May Jun Jul Aug Sep Oct Nov Dec Jan Feb Mar Apr May Jun Jul Aug Sep Oct Nov Dec
site
AL
BA
BI
KI
MI
MU
NO
PO
SRP
60
40
20
0
Feb Mar Apr May Jun Jul Aug Sep Oct Nov Dec Jan Feb Mar Apr May Jun Jul Aug Sep Oct Nov Dec
site
AL
BA
BI
KI
MI
MU
NO
PO
TN
2000
1000
Feb Mar Apr May Jun Jul Aug Sep Oct Nov Dec Jan Feb Mar Apr May Jun Jul Aug Sep Oct Nov Dec
site
AL
BA
BI
KI
MI
MU
NO
PO

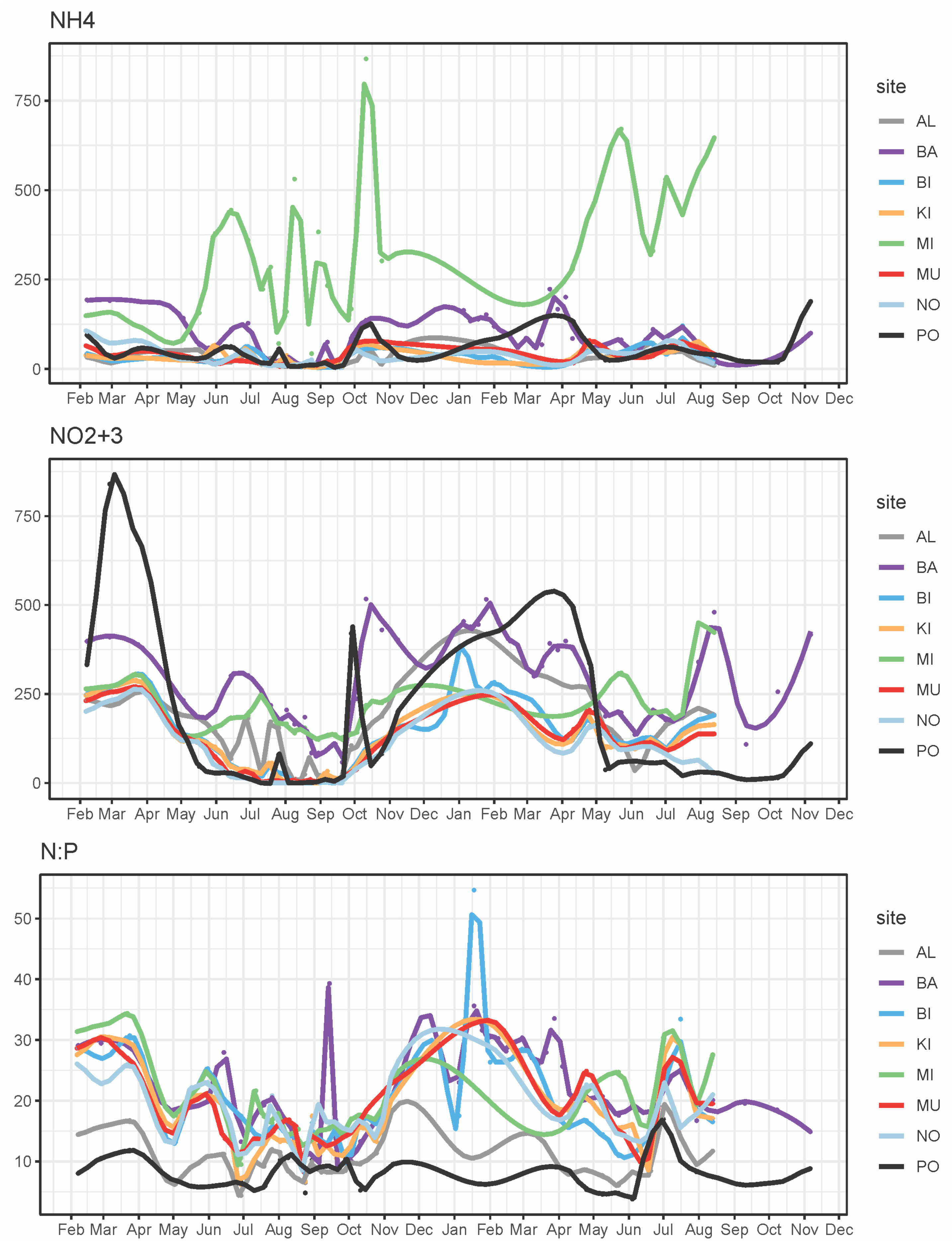
NH4
750
500
250
0
Feb Mar Apr May Jun Jul Aug Sep Oct Nov Dec Jan Feb Mar Apr May Jun Jul Aug Sep Oct Nov Dec
site
AL
BA
BI
KI
MI
MU
NO
PO
NO2+3
750
500
250
0
Feb Mar Apr May Jun Jul Aug Sep Oct Nov Dec Jan Feb Mar Apr May Jun Jul Aug Sep Oct Nov Dec
site
AL
BA
BI
KI
MI
MU
NO
PO
N:P
50
40
30
20
10
Feb Mar Apr May Jun Jul Aug Sep Oct Nov Dec Jan Feb Mar Apr May Jun Jul Aug Sep Oct Nov Dec
site
AL
BA
BI
KI
MI
MU
NO
PO

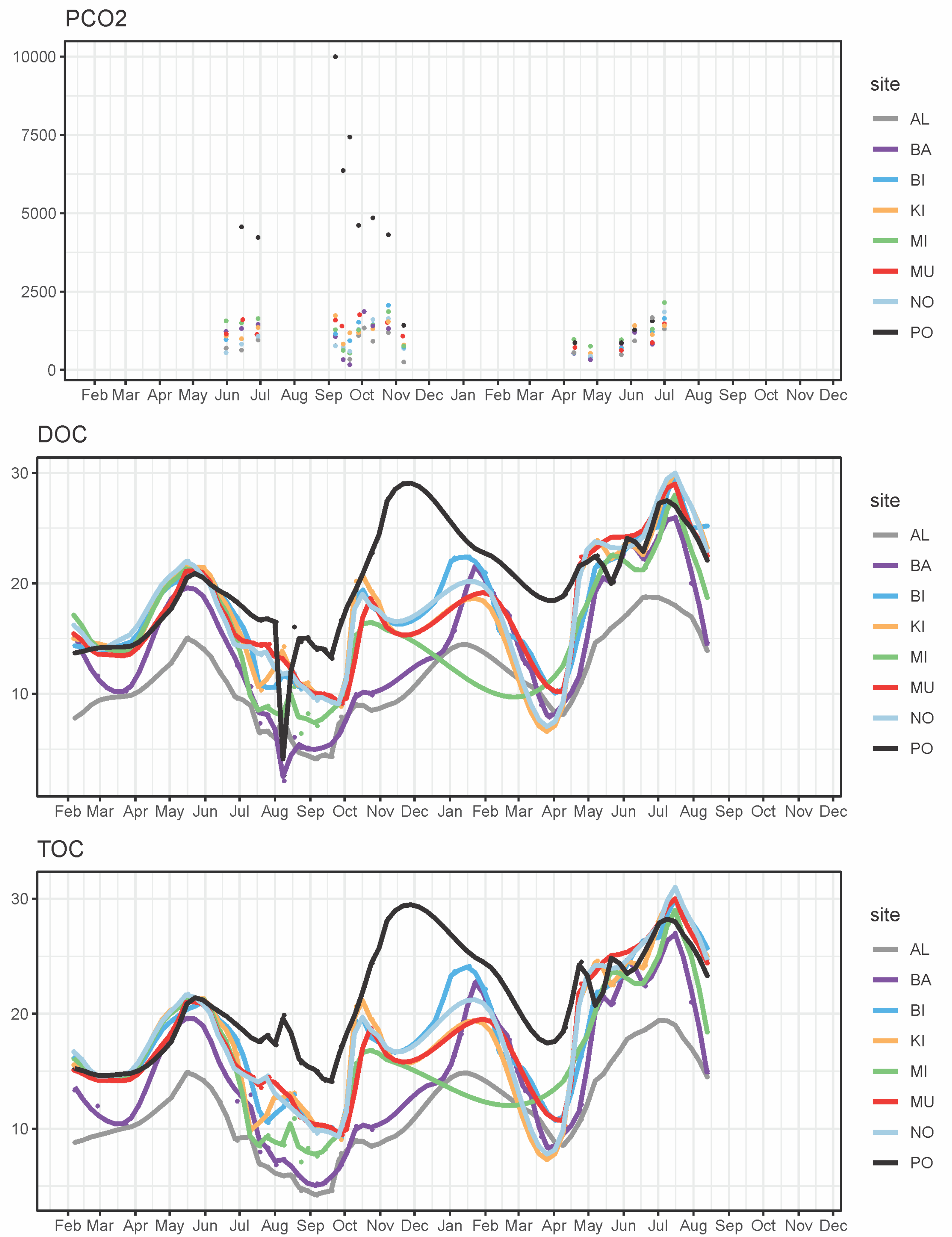
PCO2
10000
7500
5000
2500
0
Feb Mar Apr May Jun Jul Aug Sep Oct Nov Dec Jan Feb Mar Apr May Jun Jul Aug Sep Oct Nov Dec
site
AL
BA
BI
KI
MI
MU
NO
PO
DOC
30
20
10
Feb Mar Apr May Jun Jul Aug Sep Oct Nov Dec Jan Feb Mar Apr May Jun Jul Aug Sep Oct Nov Dec
site
AL
BA
BI
KI
MI
MU
NO
PO
TOC
30
20
10
Feb Mar Apr May Jun Jul Aug Sep Oct Nov Dec Jan Feb Mar Apr May Jun Jul Aug Sep Oct Nov Dec
site
AL
BA
BI
KI
MI
MU
NO
PO

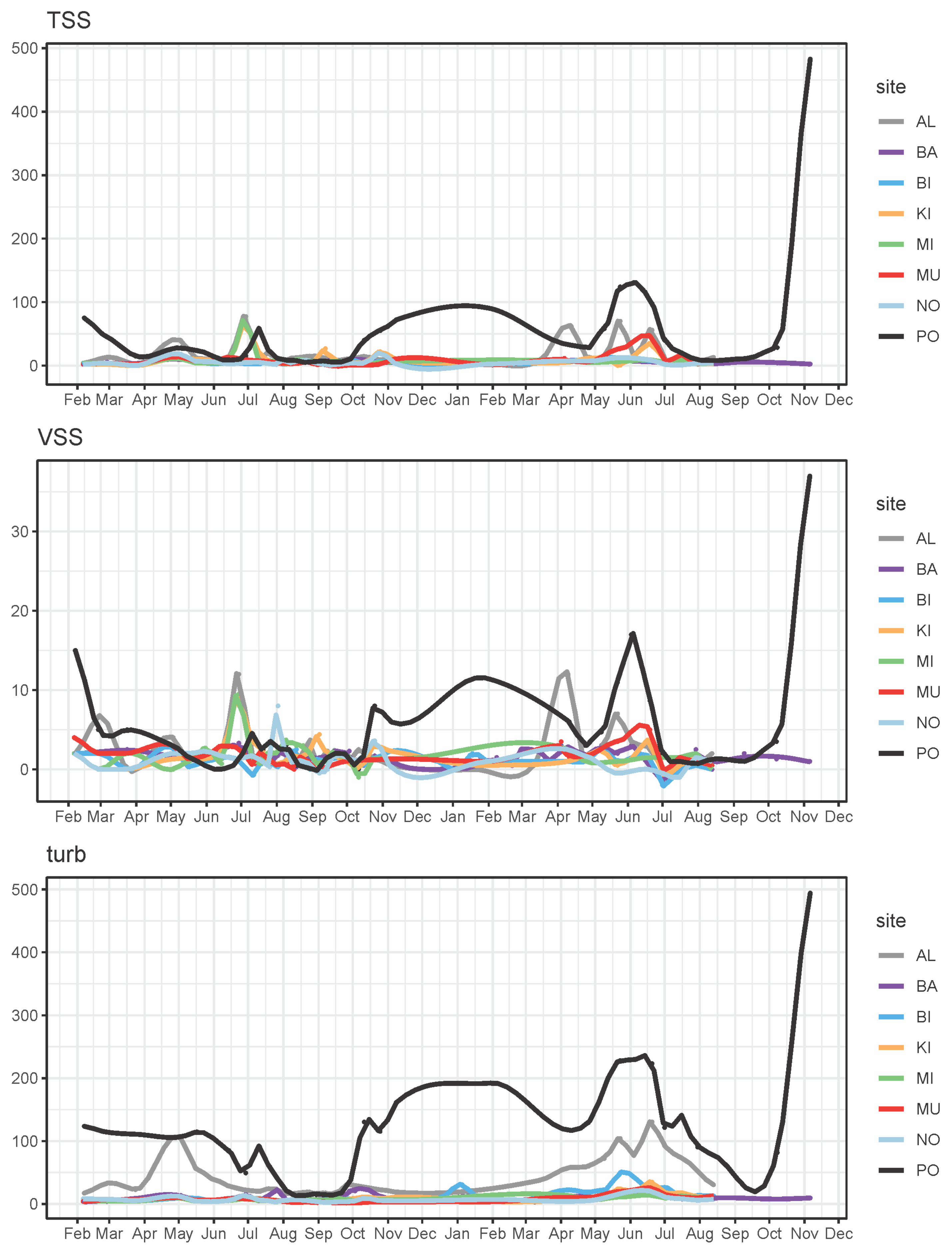

TSS
500
400
300
200
100
0
Feb Mar Apr May Jun Jul Aug Sep Oct Nov Dec Jan Feb Mar Apr May Jun Jul Aug Sep Oct Nov Dec
site
AL
BA
BI
KI
MI
MU
NO
PO
VSS
30
20
10
0
turb

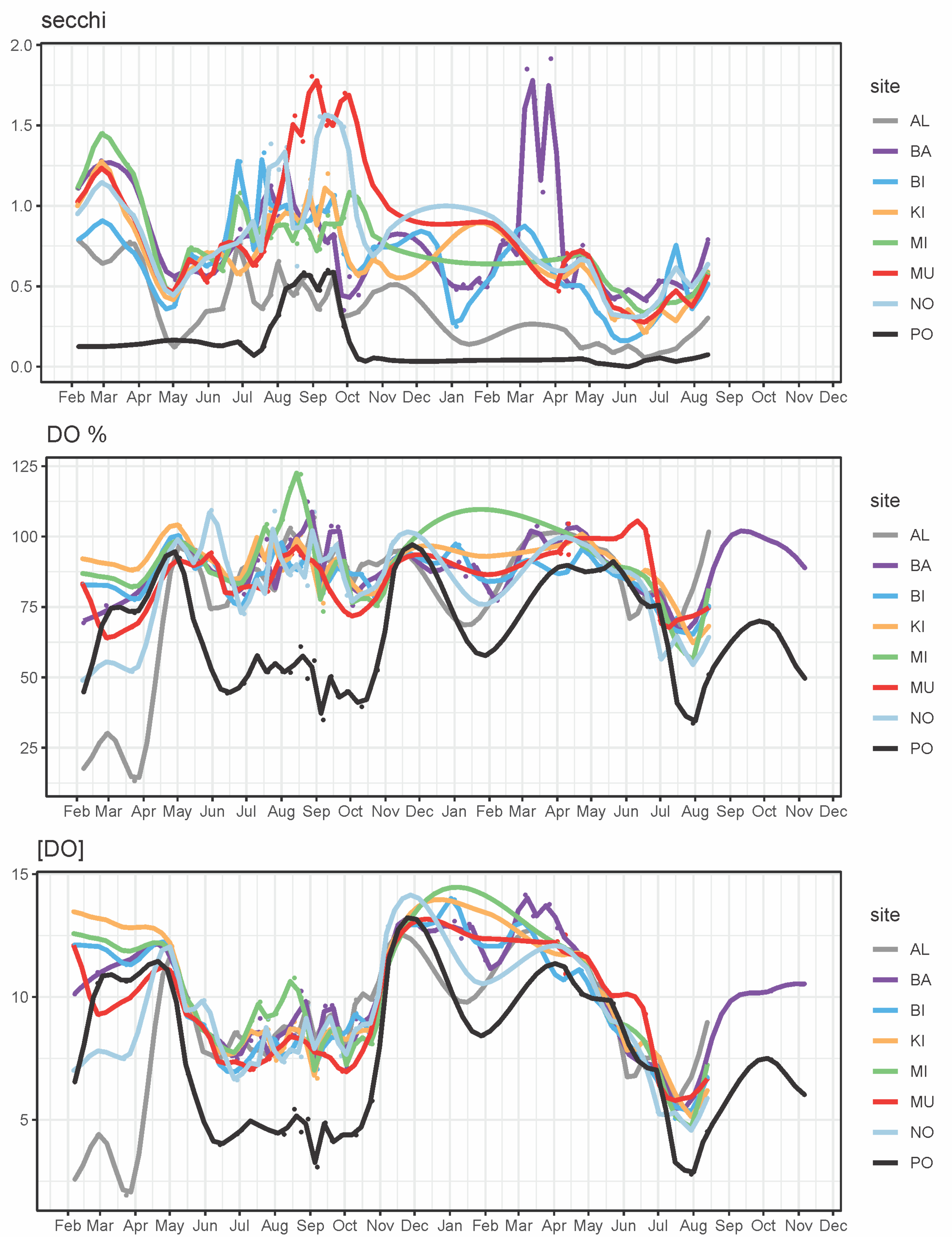
secchi
2.0
1.5
1.0
0.5
0.0
Feb Mar Apr May Jun Jul Aug Sep Oct Nov Dec Jan Feb Mar Apr May Jun Jul Aug Sep Oct Nov Dec
site
AL
BA
BI
KI
MI
MU
NO
PO
DO %
125
100
75
50
25
Feb Mar Apr May Jun Jul Aug Sep Oct Nov Dec Jan Feb Mar Apr May Jun Jul Aug Sep Oct Nov Dec
site
AL
BA
BI
KI
MI
MU
NO
PO
[DO]
15
10
5
Feb Mar Apr May Jun Jul Aug Sep Oct Nov Dec Jan Feb Mar Apr May Jun Jul Aug Sep Oct Nov Dec
site
AL
BA
BI
KI
MI
MU
NO
PO

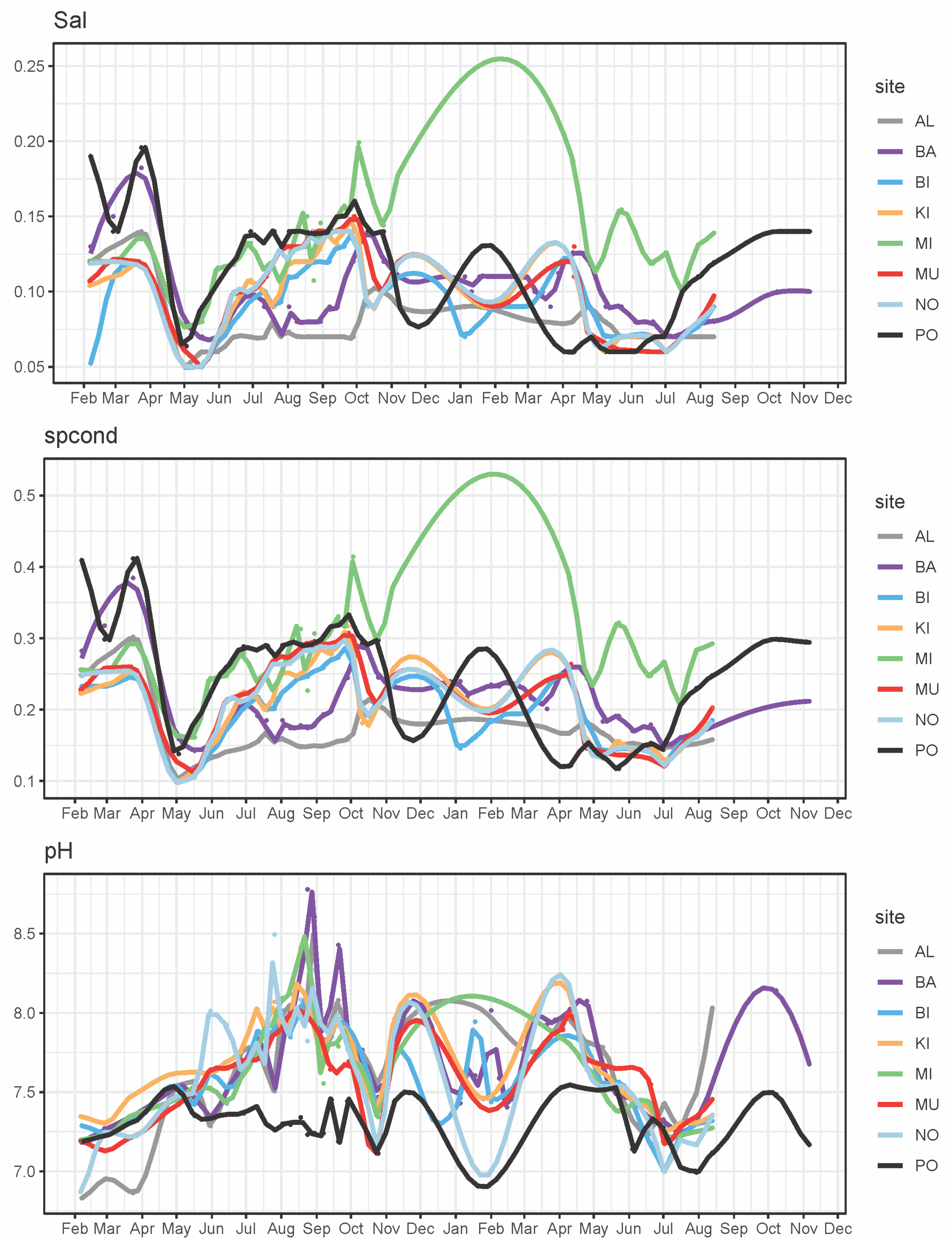
Sal
0.25
0.20
0.15
0.10
0.05
Feb Mar Apr May Jun Jul Aug Sep Oct Nov Dec Jan Feb Mar Apr May Jun Jul Aug Sep Oct Nov Dec
site
AL
BA
BI
KI
MI
MU
NO
PO
spcond
0.5
0.4
0.3
0.2
0.1
Feb Mar Apr May Jun Jul Aug Sep Oct Nov Dec Jan Feb Mar Apr May Jun Jul Aug Sep Oct Nov Dec
site
AL
BA
BI
KI
MI
MU
NO
PO
pH
8.5
8.0
7.5
7.0
Feb Mar Apr May Jun Jul Aug Sep Oct Nov Dec Jan Feb Mar Apr May Jun Jul Aug Sep Oct Nov Dec
site
AL
BA
BI
KI
MI
MU
NO
PO

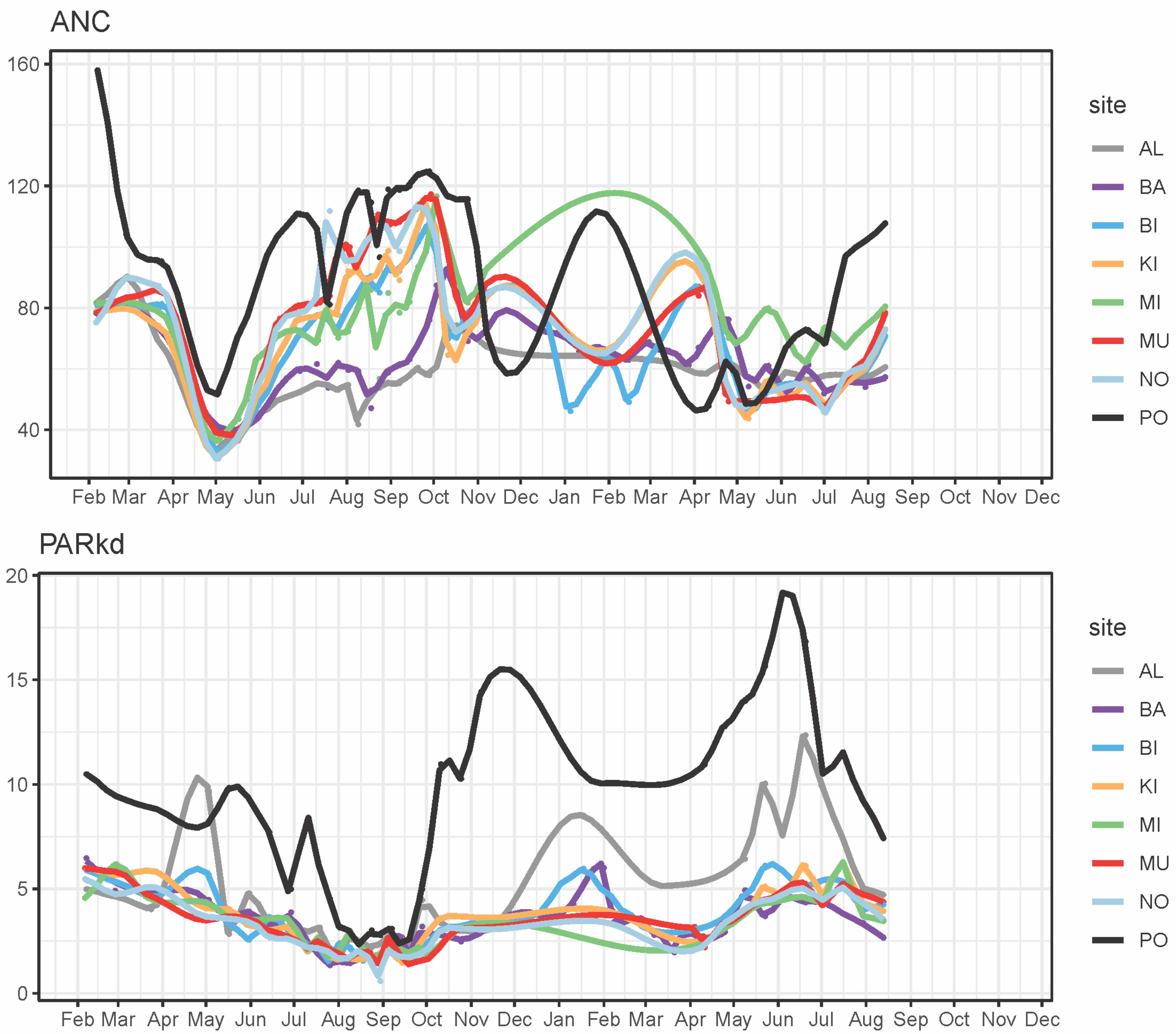
ANC
160
120
80
40
Feb Mar Apr May Jun Jul Aug Sep Oct Nov Dec Jan Feb Mar Apr May Jun Jul Aug Sep Oct Nov Dec
site
AL
BA
BI
KI
MI
MU
NO
PO
PARkd
20
15
10
5
0
Feb Mar Apr May Jun Jul Aug Sep Oct Nov Dec Jan Feb Mar Apr May Jun Jul Aug Sep Oct Nov Dec
site
AL
BA
BI
KI
MI
MU
NO
PO
