# Supplementary material for: Optimizing a long‐term monitoring strategy to track emerging cyanobacterial blooms in a Great Lakes estuary
Source: J Phycol. 2026 Aug 7;62(4):1264–96. doi: 10.1111/jpy.70215 (PMC13485230; doi:10.1111/jpy.70215)
Supplement: Supplementary file 2 [file JPY-62-1264-s005.pdf]

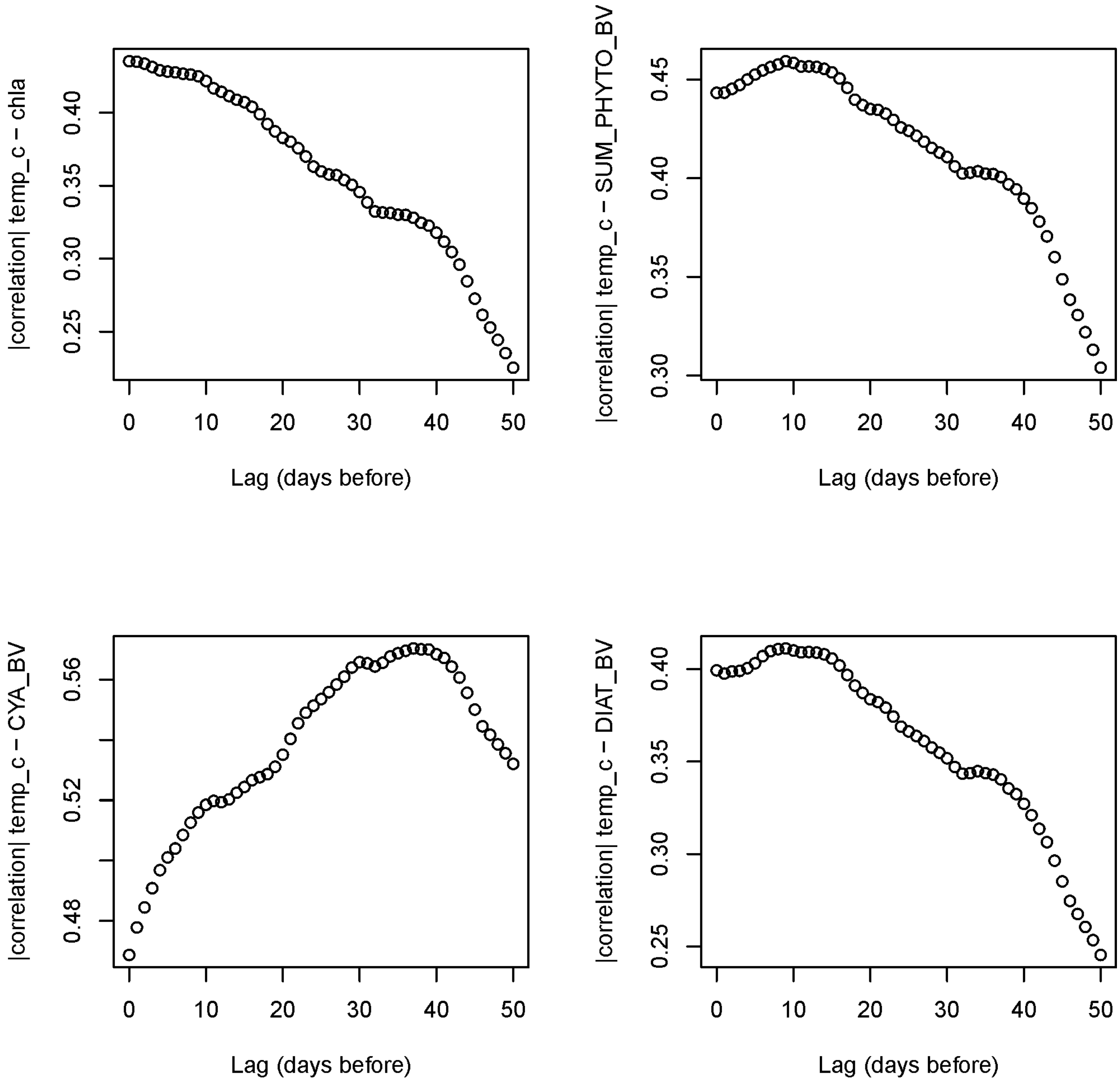
|correlation| temp_c − chla
0.25
0.30
0.35
0.40
0
10
20
30
40
50
Lag (days before)
|correlation| temp_c − SUM_PHYTO_BV
0.30
0.35
0.40
0.45
0
10
20
30
40
50
Lag (days before)
|correlation| temp_c − CYA_BV
0.48
0.52
0.56
0
10
20
30
40
50
Lag (days before)
|correlation| temp_c − DIAT_BV
0.25
0.30
0.35
0.40
0
10
20
30
40
50
Lag (days before)

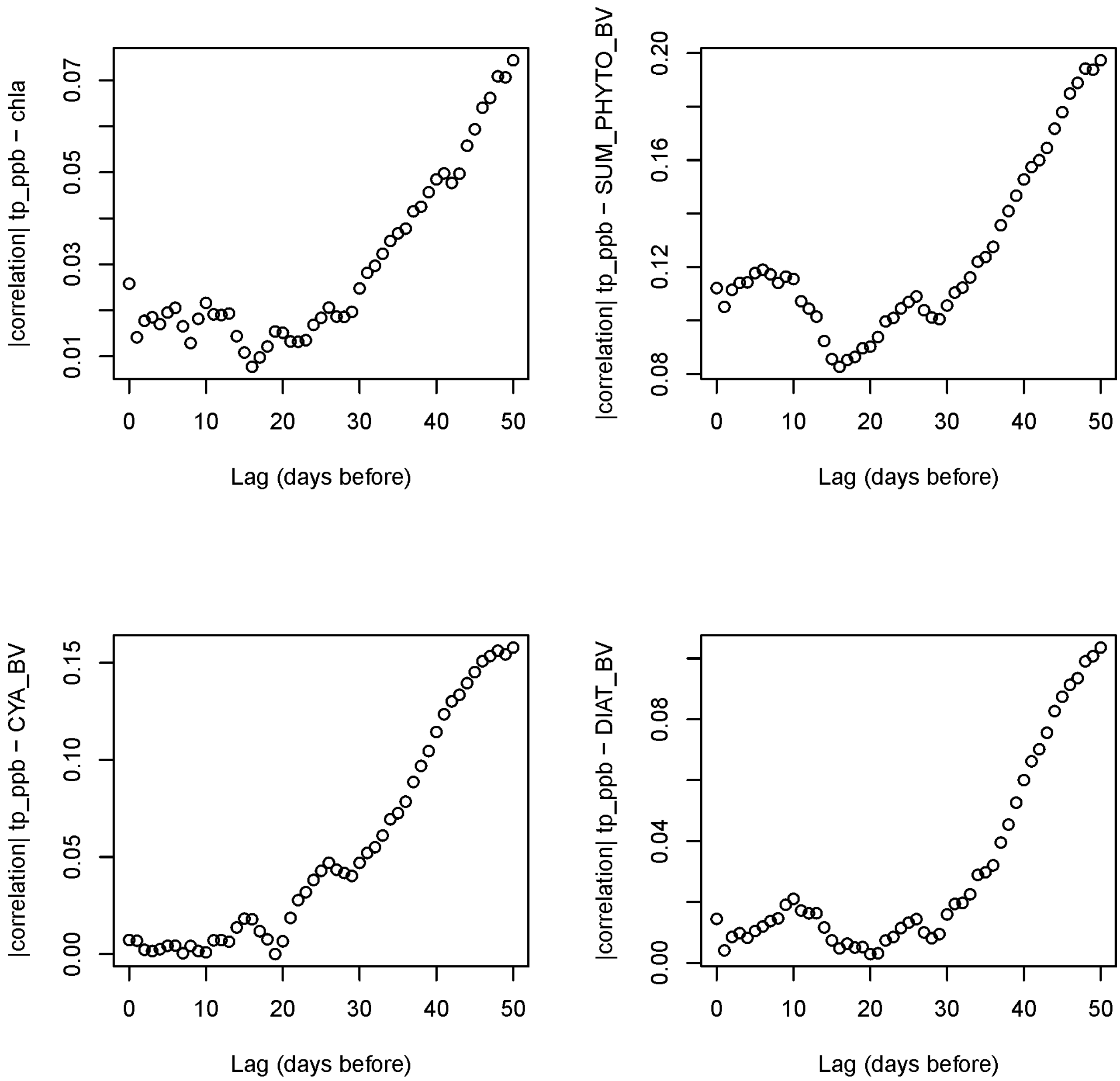
|correlation| tp_ppb − chla
|correlation| tp_ppb − SUM_PHYTO_BV
|correlation| tp_ppb − CYA_BV
|correlation| tp_ppb − DIAT_BV
Lag (days before)
0.01
0.03
0.05
0.07
0.08
0.12
0.16
0.20
0.00
0.05
0.10
0.15
0.04
0.08
0
10
20
30
40
50

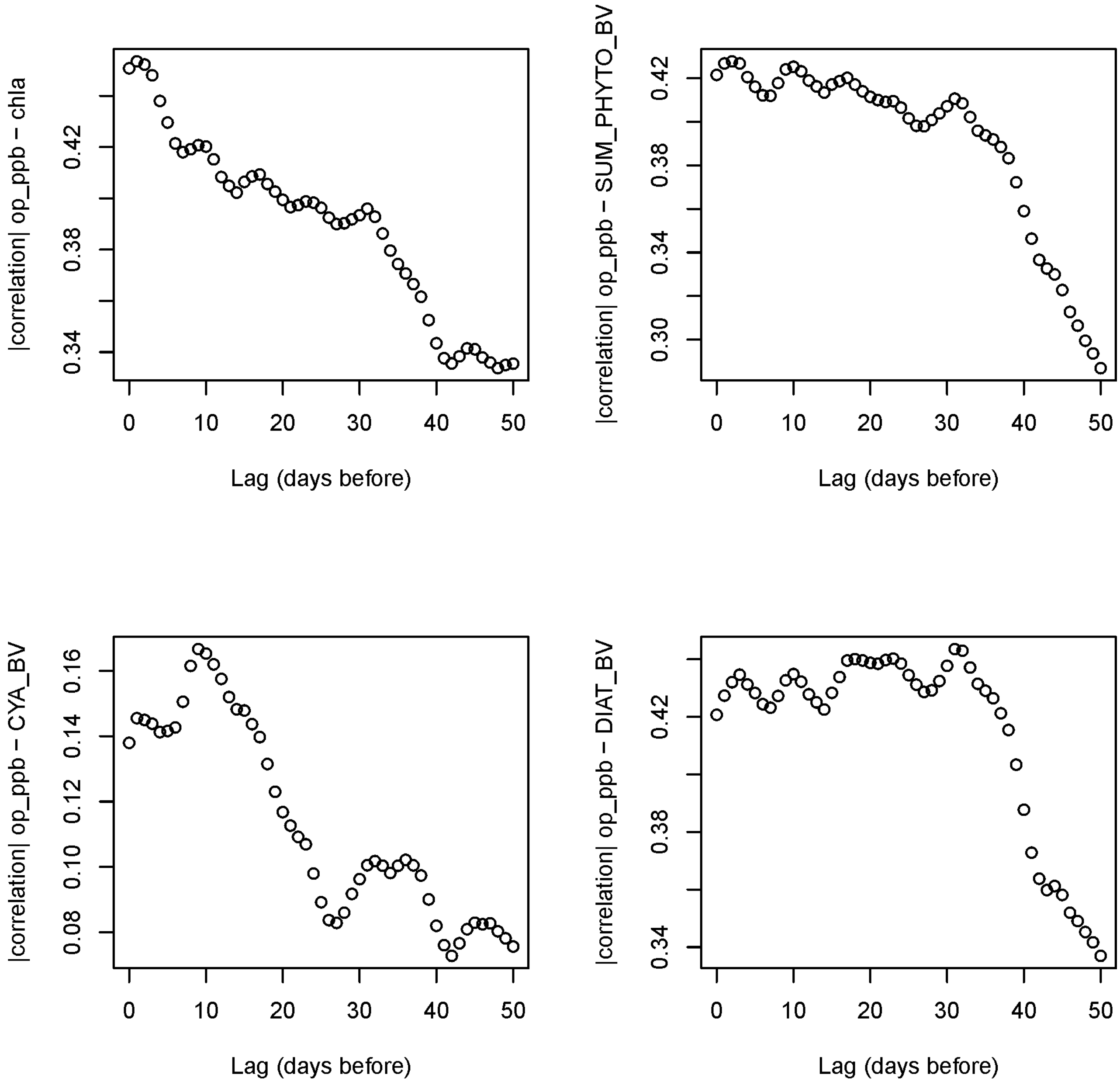

|correlation| op_ppb − chla
0.34
0.38
0.42
Lag (days before)
|correlation| op_ppb − SUM_PHYTO_BV
0.30
0.34
0.38
0.42
Lag (days before)
|correlation| op_ppb − CYA_BV
0.08
0.10
0.12
0.14
0.16
Lag (days before)
|correlation| op_ppb − DIAT_BV
0.34
0.38
0.42
Lag (days before)
0
10
20
30
40
50

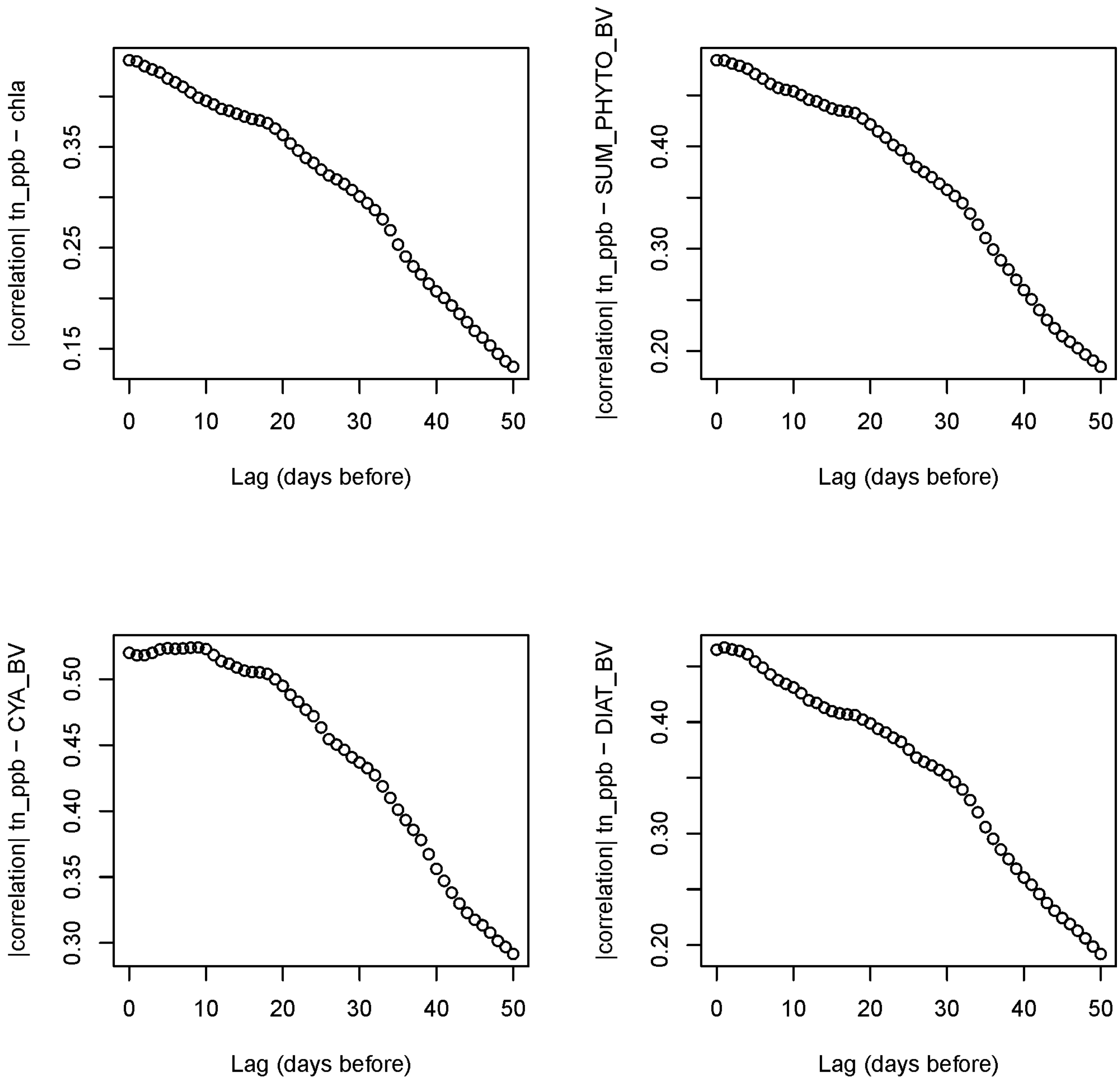

|correlation| tn_ppb − chla
|correlation| tn_ppb − SUM_PHYTO_BV
|correlation| tn_ppb − CYA_BV
|correlation| tn_ppb − DIAT_BV
0.15
0.25
0.35
0.20
0.30
0.40
0.30
0.35
0.40
0.45
0.50
0
10
20
30
40
50
Lag (days before)

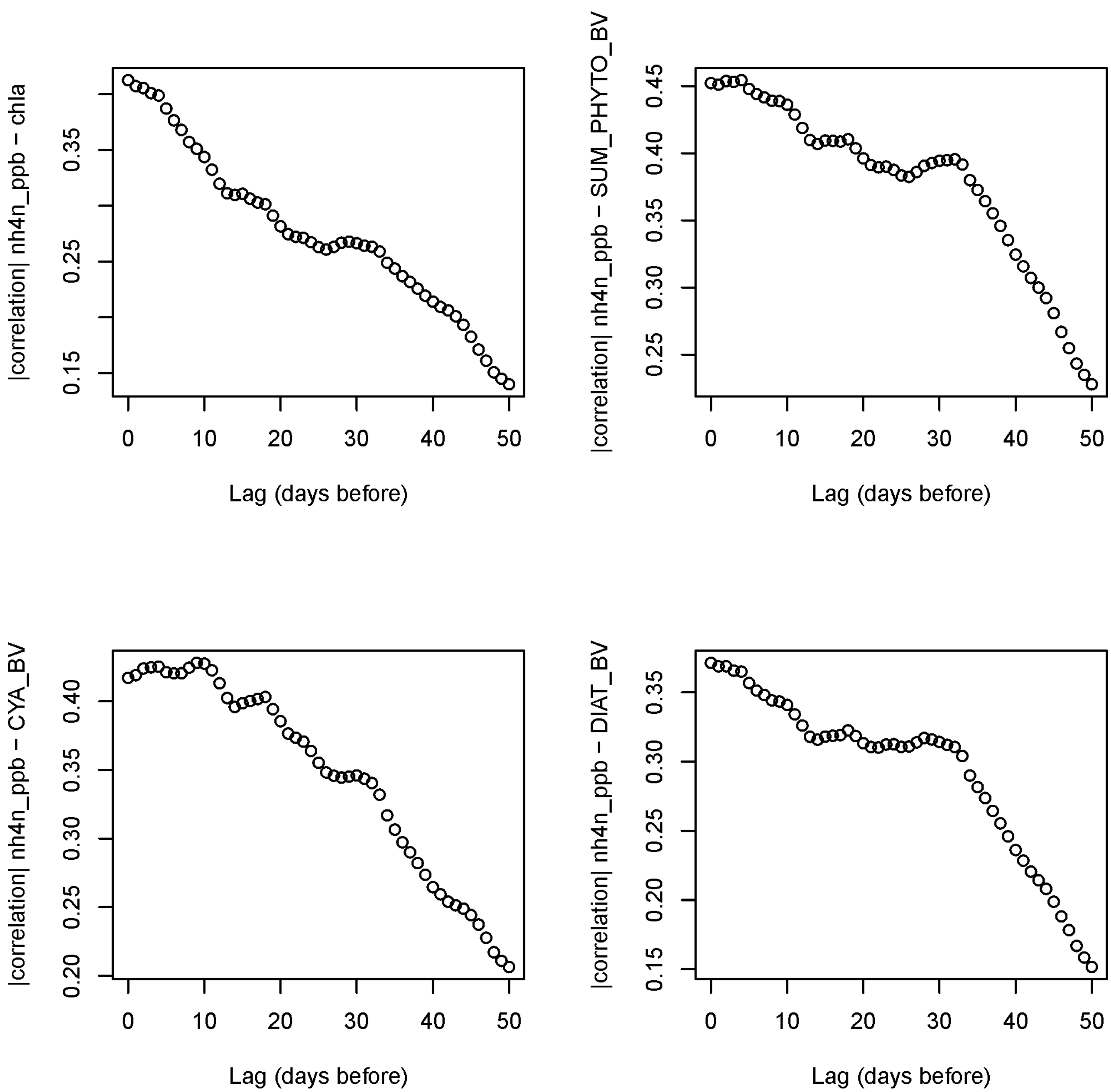
|correlation| nh4n_ppb – chla
|correlation| nh4n_ppb – SUM_PHYTO_BV
|correlation| nh4n_ppb – CYA_BV
|correlation| nh4n_ppb – DIAT_BV
Lag (days before)
Lag (days before)
Lag (days before)
Lag (days before)

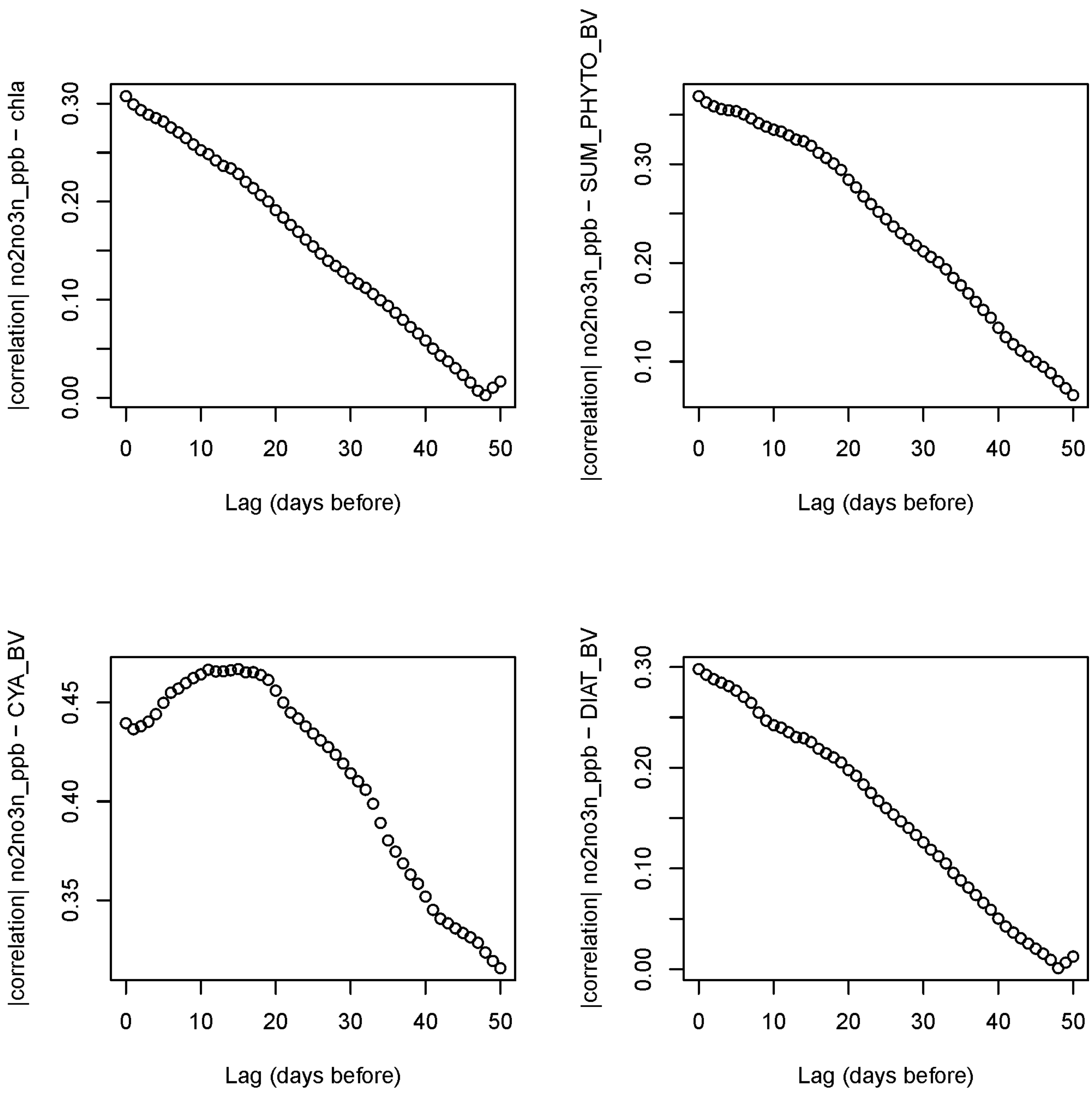
|correlation| no2no3n_ppb − chla
|correlation| no2no3n_ppb − SUM_PHYTO_BV
|correlation| no2no3n_ppb − CYA_BV
|correlation| no2no3n_ppb − DIAT_BV
Lag (days before)
0.00
0.10
0.20
0.30
0.35
0.40
0.45
0
10
20
30
40
50

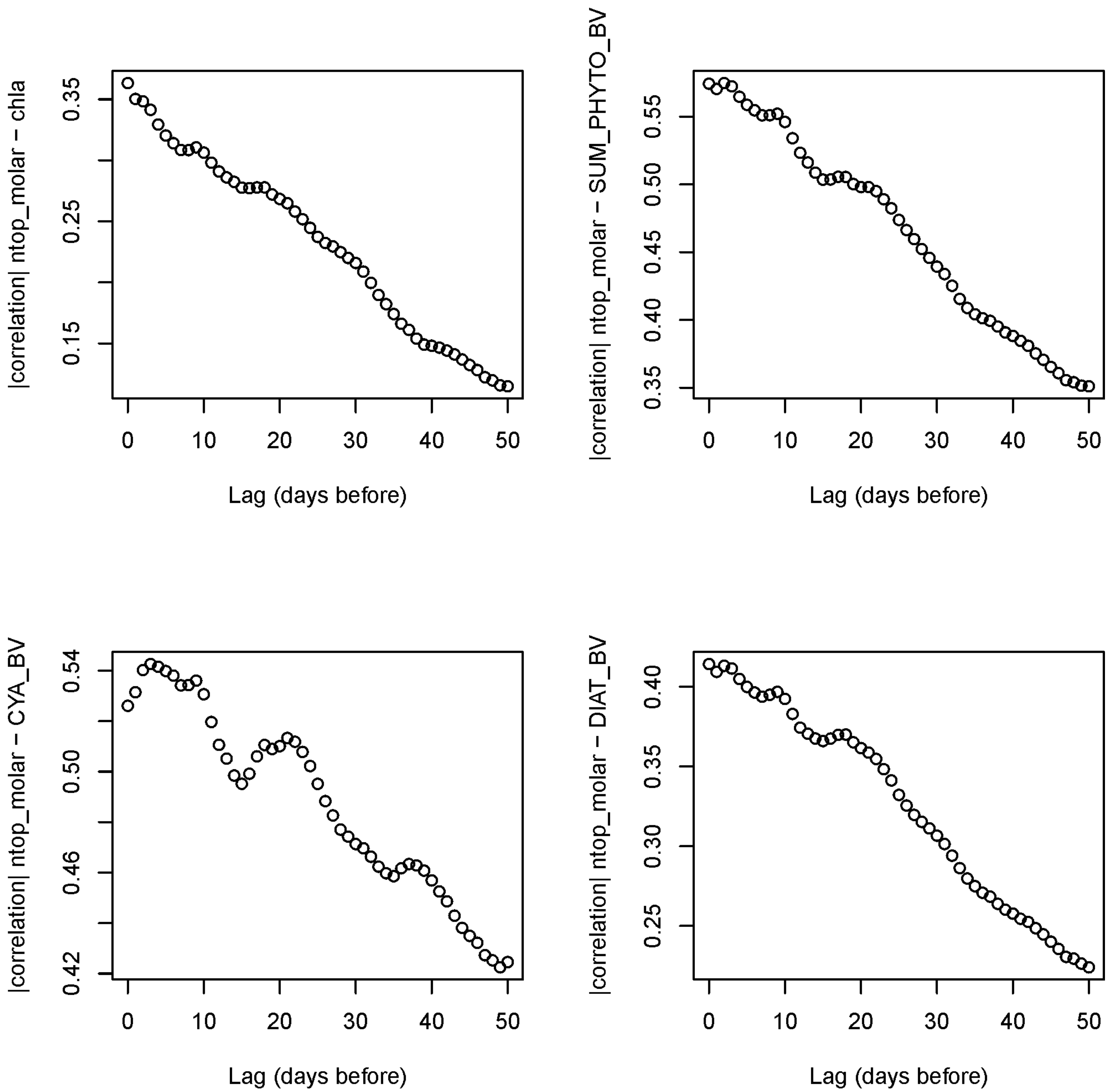

|correlation| ntop_molar – chla
0.15
0.25
0.35
0
10
20
30
40
50
Lag (days before)
|correlation| ntop_molar – SUM_PHYTO_BV
0.35
0.40
0.45
0.50
0.55
0
10
20
30
40
50
Lag (days before)
|correlation| ntop_molar – CYA_BV
0.42
0.46
0.50
0.54
0
10
20
30
40
50
Lag (days before)
|correlation| ntop_molar – DIAT_BV
0.25
0.30
0.35
0.40
0
10
20
30
40
50
Lag (days before)

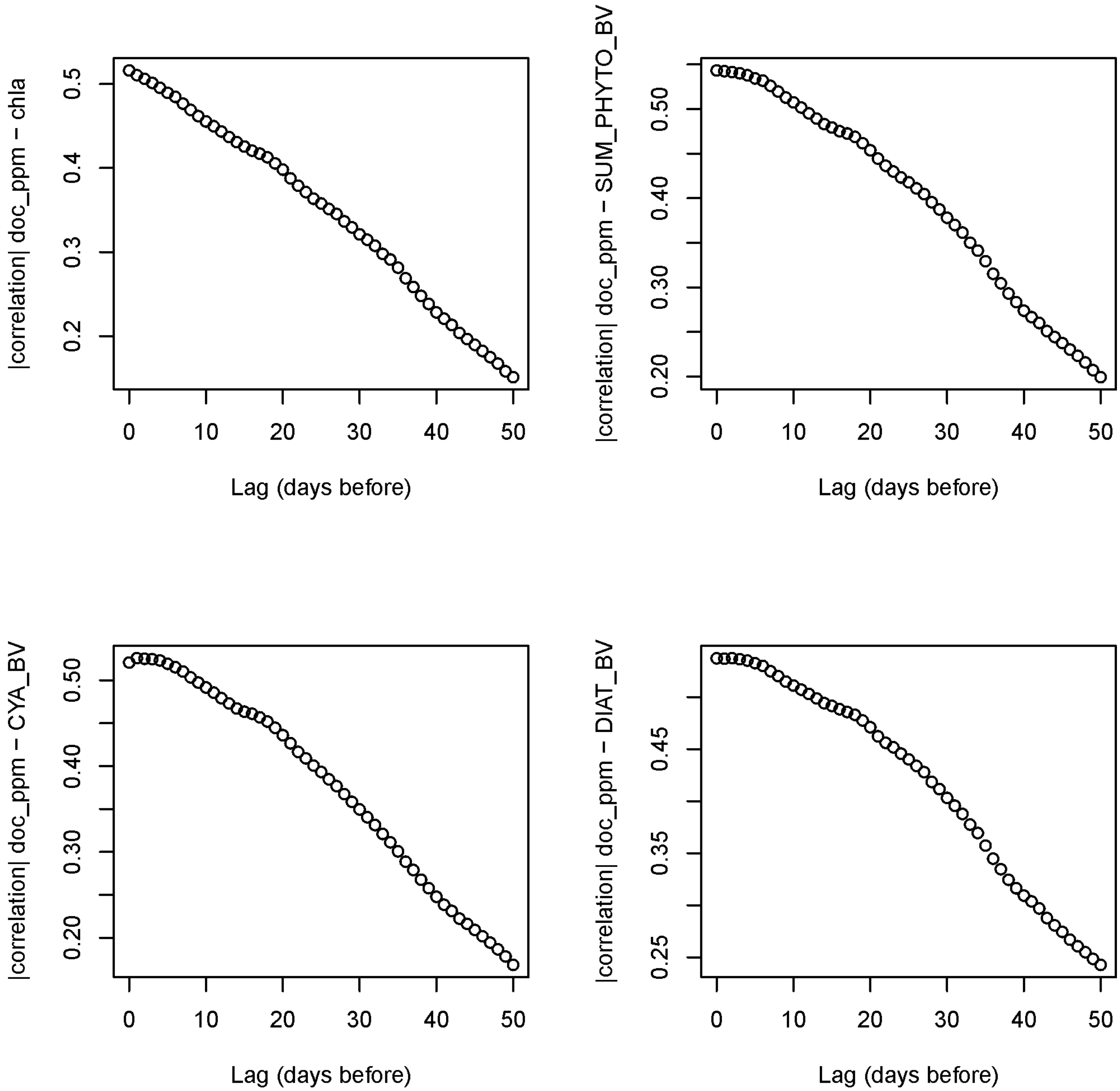
|correlation| doc_ppm − chla
0.5
0.4
0.3
0.2
0
10
20
30
40
50
Lag (days before)
|correlation| doc_ppm − SUM_PHYTO_BV
0.50
0.40
0.30
0.20
0
10
20
30
40
50
Lag (days before)
|correlation| doc_ppm − CYA_BV
0.50
0.40
0.30
0.20
0
10
20
30
40
50
Lag (days before)
|correlation| doc_ppm − DIAT_BV
0.45
0.35
0.25
0
10
20
30
40
50
Lag (days before)

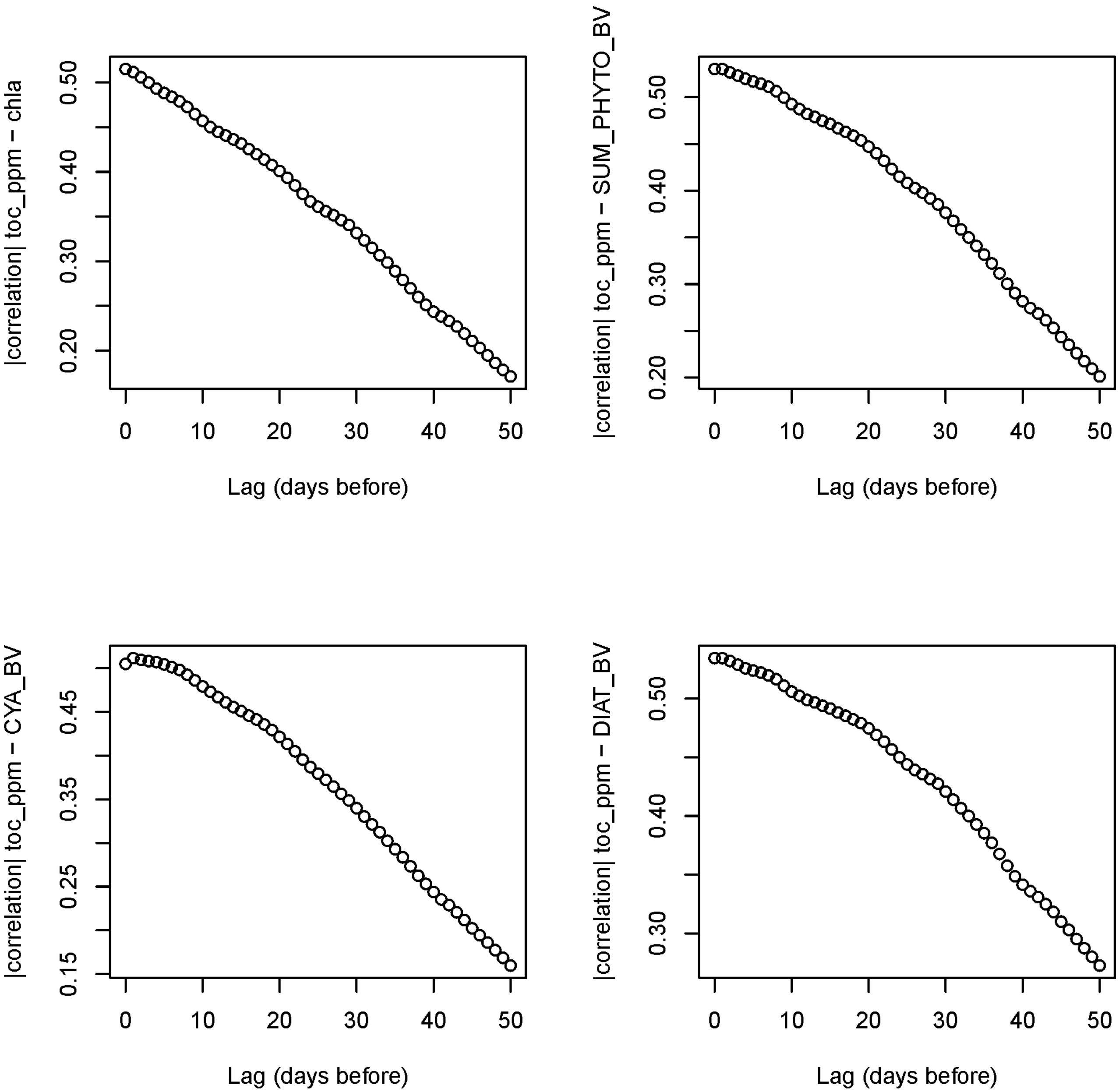
|correlation| toc_ppm − chla
0.20
0.30
0.40
0.50
0
10
20
30
40
50
Lag (days before)
|correlation| toc_ppm − SUM_PHYTO_BV
0.20
0.30
0.40
0.50
0
10
20
30
40
50
Lag (days before)
|correlation| toc_ppm − CYA_BV
0.15
0.25
0.35
0.45
0
10
20
30
40
50
Lag (days before)
|correlation| toc_ppm − DIAT_BV
0.30
0.40
0.50
0
10
20
30
40
50
Lag (days before)

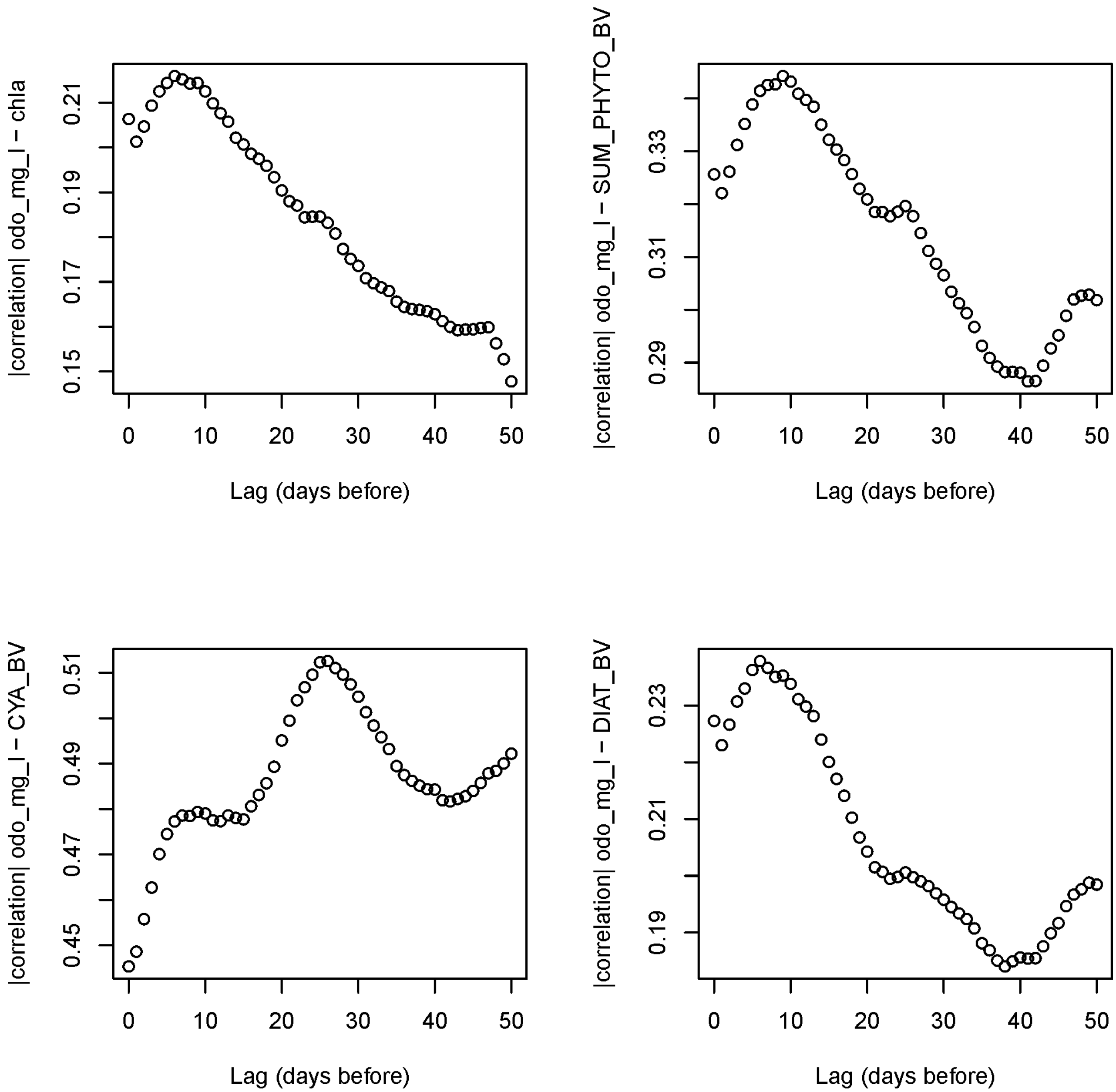
|correlation| odo_mg_l – chla
0.15
0.17
0.19
0.21
0
10
20
30
40
50
Lag (days before)
|correlation| odo_mg_l – SUM_PHYTO_BV
0.29
0.31
0.33
0
10
20
30
40
50
Lag (days before)
|correlation| odo_mg_l – CYA_BV
0.45
0.47
0.49
0.51
0
10
20
30
40
50
Lag (days before)
|correlation| odo_mg_l – DIAT_BV
0.19
0.21
0.23
0
10
20
30
40
50
Lag (days before)

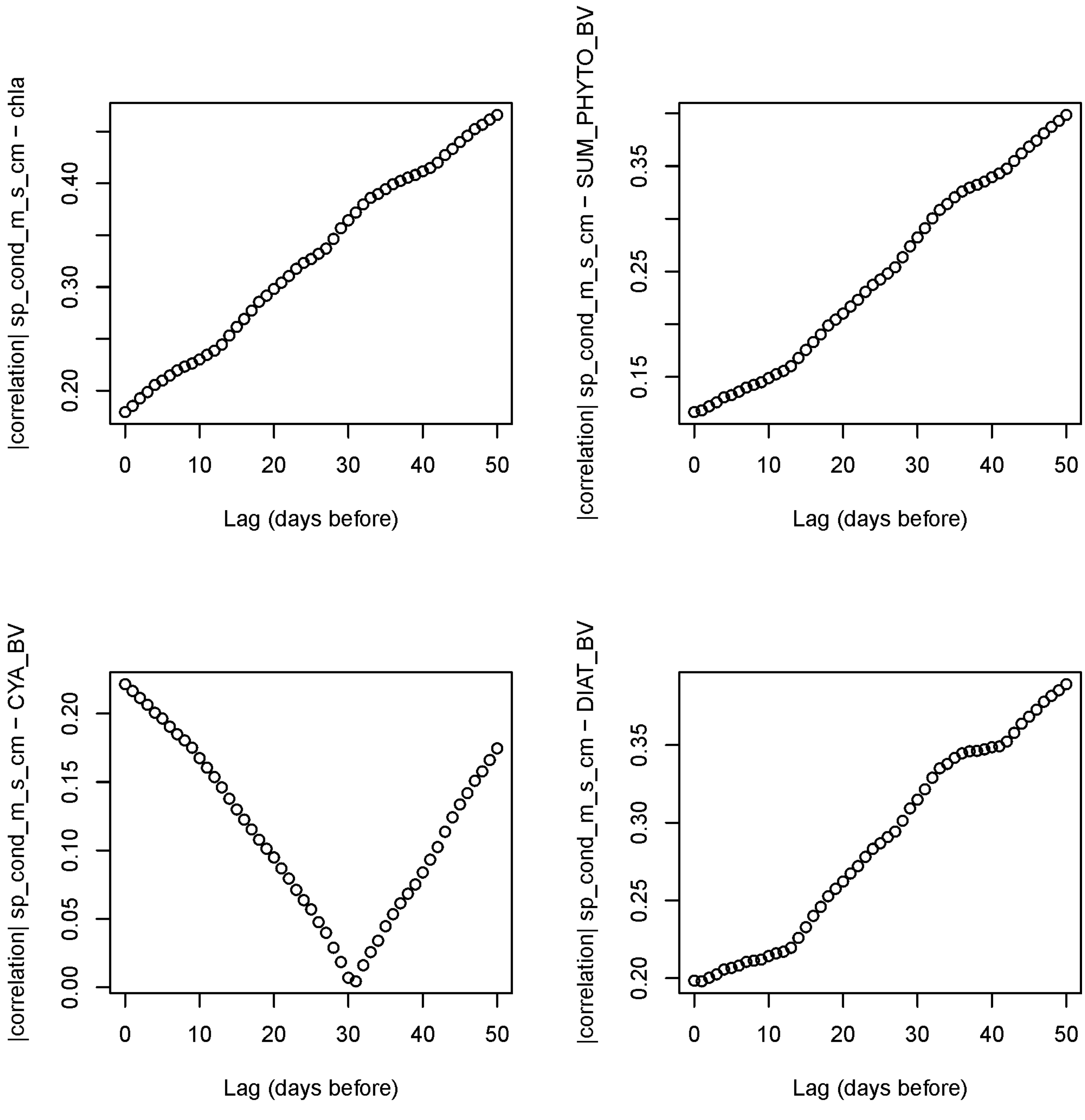
|correlation| sp_cond_m_s_cm − chla
0.20
0.30
0.40
0
10
20
30
40
50
Lag (days before)
|correlation| sp_cond_m_s_cm − SUM_PHYTO_BV
0.15
0.25
0.35
0
10
20
30
40
50
Lag (days before)
|correlation| sp_cond_m_s_cm − CYA_BV
0.00
0.05
0.10
0.15
0.20
0
10
20
30
40
50
Lag (days before)
|correlation| sp_cond_m_s_cm − DIAT_BV
0.20
0.25
0.30
0.35
0
10
20
30
40
50
Lag (days before)

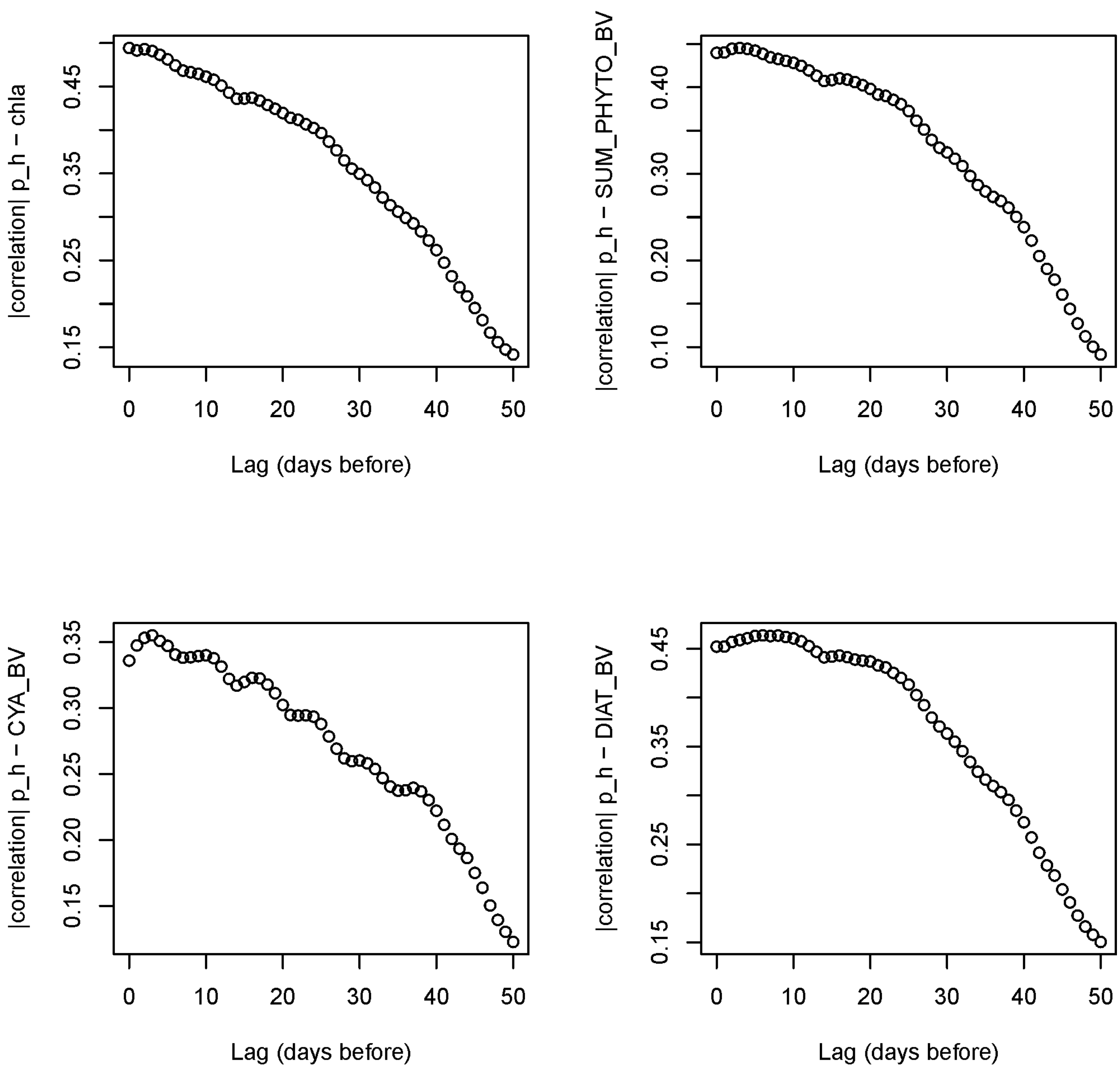

|correlation| p_h − chla
Lag (days before)
|correlation| p_h − SUM_PHYTO_BV
Lag (days before)
|correlation| p_h − CYA_BV
Lag (days before)
|correlation| p_h − DIAT_BV
Lag (days before)

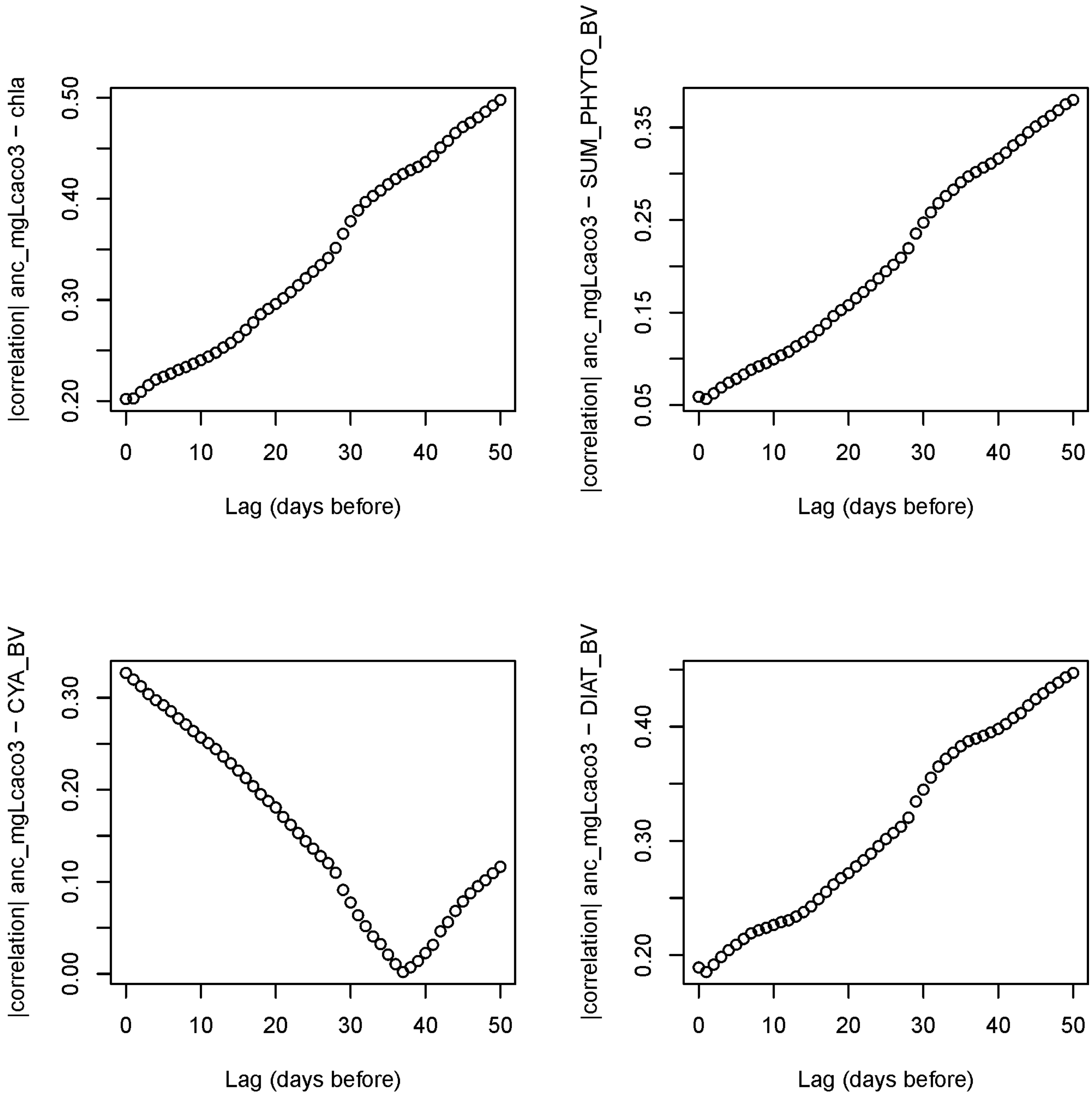
|correlation| anc_mgLcaco3 − chla
0.20
0.30
0.40
0.50
0
10
20
30
40
50
Lag (days before)
|correlation| anc_mgLcaco3 − SUM_PHYTO_BV
0.05
0.15
0.25
0.35
0
10
20
30
40
50
Lag (days before)
|correlation| anc_mgLcaco3 − CYA_BV
0.00
0.10
0.20
0.30
0
10
20
30
40
50
Lag (days before)
|correlation| anc_mgLcaco3 − DIAT_BV
0.20
0.30
0.40
0
10
20
30
40
50
Lag (days before)

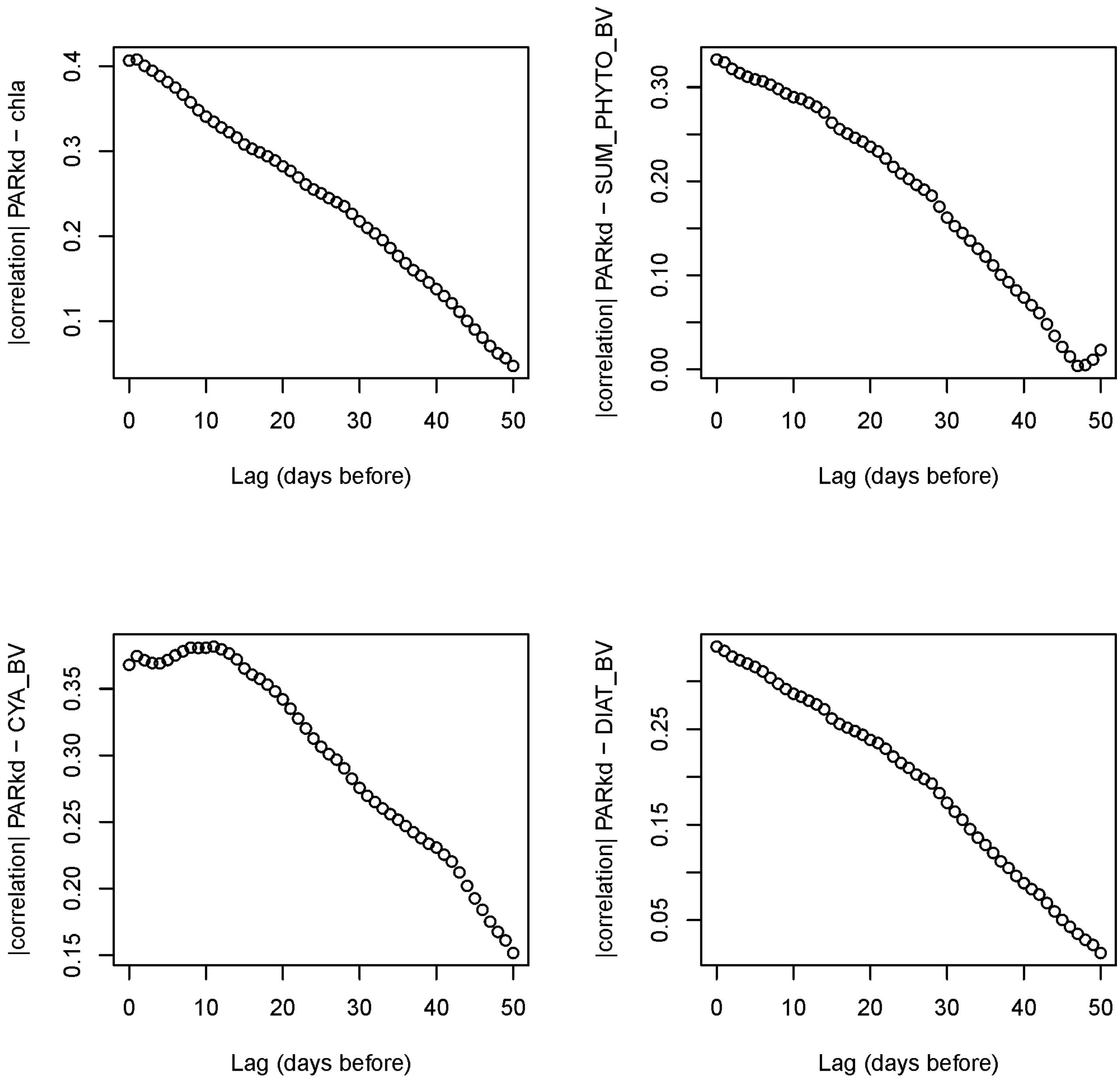
|correlation| PARkd − chla
0.1
0.2
0.3
0.4
0
10
20
30
40
50
Lag (days before)
|correlation| PARkd − SUM_PHYTO_BV
0.00
0.10
0.20
0.30
0
10
20
30
40
50
Lag (days before)
|correlation| PARkd − CYA_BV
0.15
0.20
0.25
0.30
0.35
0
10
20
30
40
50
Lag (days before)
|correlation| PARkd − DIAT_BV
0.05
0.15
0.25
0
10
20
30
40
50
Lag (days before)
