# Supplementary material for: Optimizing a long‐term monitoring strategy to track emerging cyanobacterial blooms in a Great Lakes estuary
Source: J Phycol. 2026 Aug 7;62(4):1264–96. doi: 10.1111/jpy.70215 (PMC13485230; doi:10.1111/jpy.70215)
Supplement: Supplementary file 3 [file JPY-62-1264-s008.pdf]

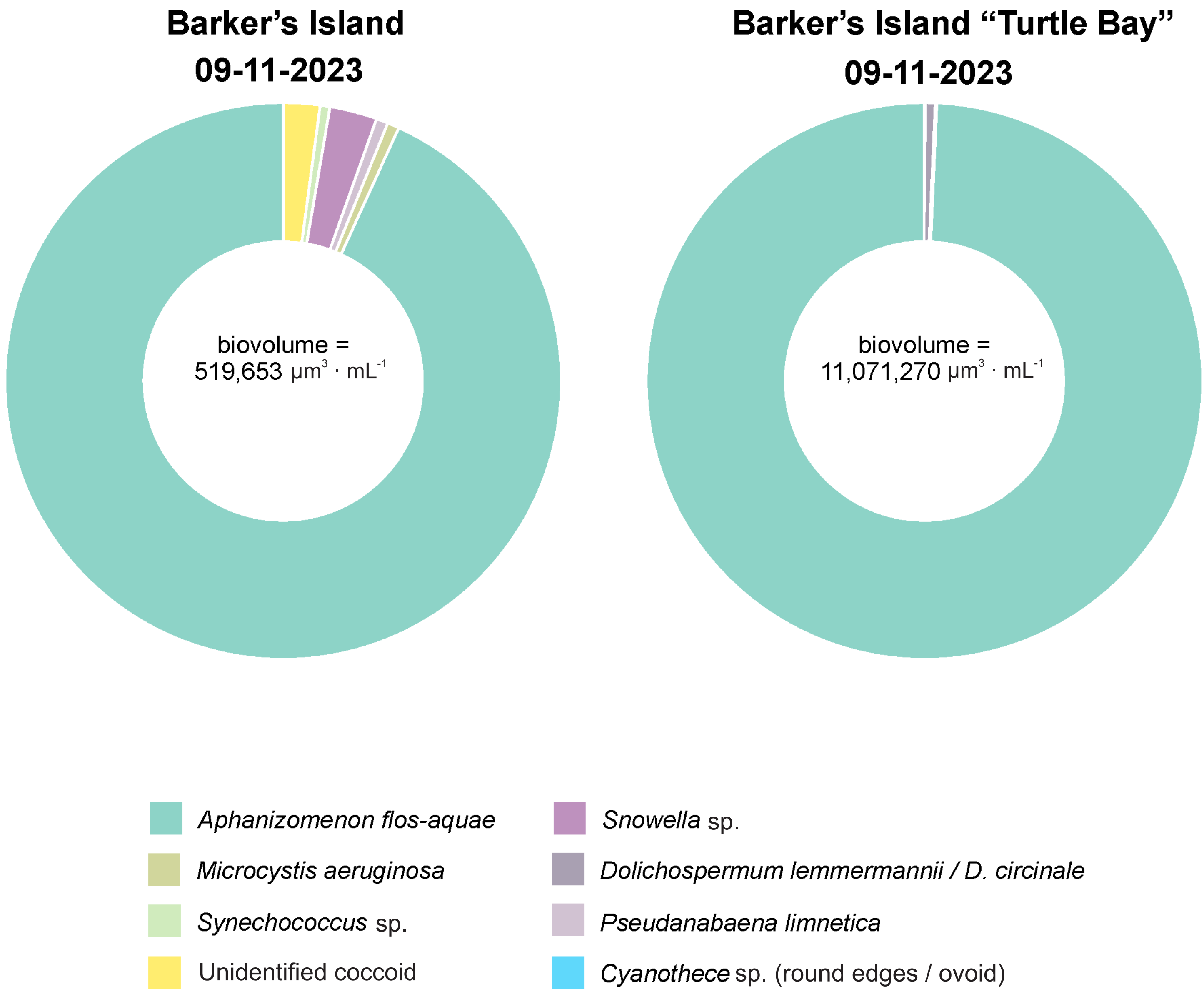

Barker's Island
09-11-2023
biovolume =
519,653 μm³ · mL⁻¹
Barker's Island "Turtle Bay"
09-11-2023
biovolume =
11,071,270 μm³ · mL⁻¹
Aphanizomenon flos-aquae
Microcystis aeruginosa
Synechococcus sp.
Unidentified coccoid
Snowella sp.
Dolichospermum lemmermannii / D. circinale
Pseudanabaena limnetica
Cyanothece sp. (round edges / ovoid)

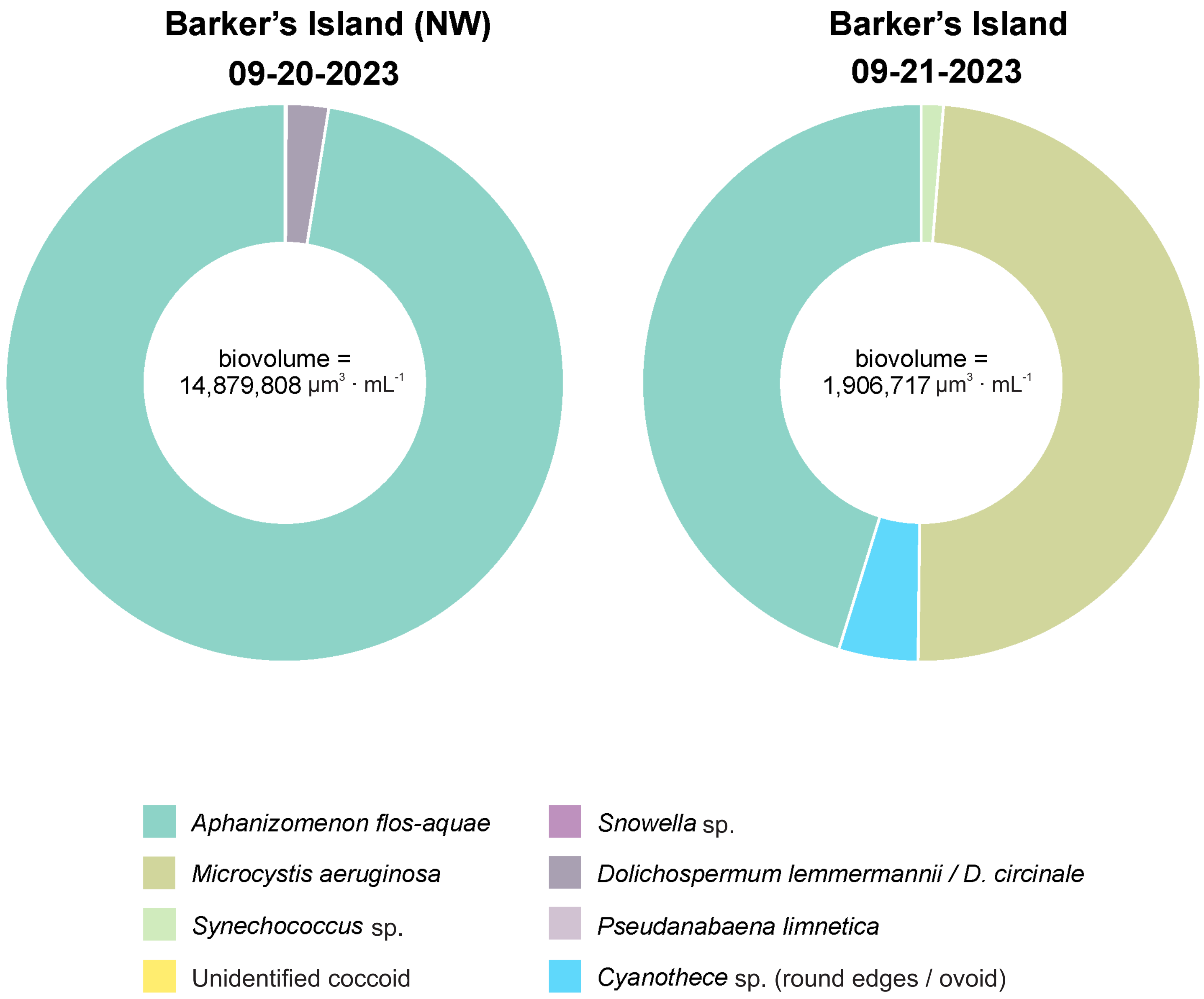

Barker's Island (NW)
09-20-2023
biovolume =
14,879,808 µm^3 · mL^{-1}
Barker's Island
09-21-2023
biovolume =
1,906,717 µm^3 · mL^{-1}
Aphanizomenon flos-aquae
Microcystis aeruginosa
Synechococcus sp.
Unidentified coccoid
Snowella sp.
Dolichospermum lemmermannii / D. circinale
Pseudanabaena limnetica
Cyanothece sp. (round edges / ovoid)

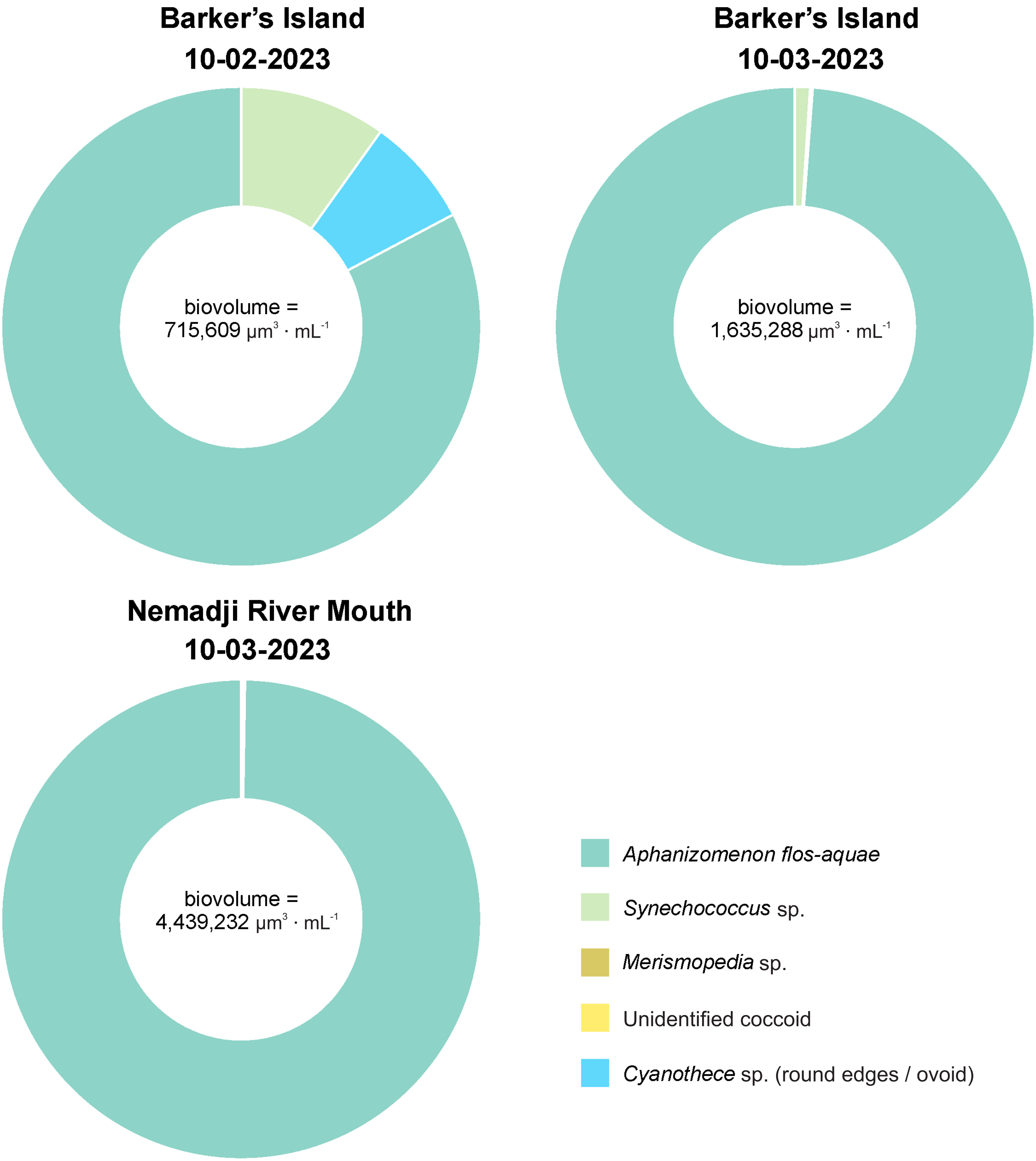
Barker's Island
10-02-2023
biovolume =
715,609 µm³ · mL⁻¹
Barker's Island
10-03-2023
biovolume =
1,635,288 µm³ · mL⁻¹
Nemadji River Mouth
10-03-2023
biovolume =
4,439,232 µm³ · mL⁻¹
Aphanizomenon flos-aquae
Synechococcus sp.
Merismopedia sp.
Unidentified coccoid
Cyanothece sp. (round edges / ovoid)

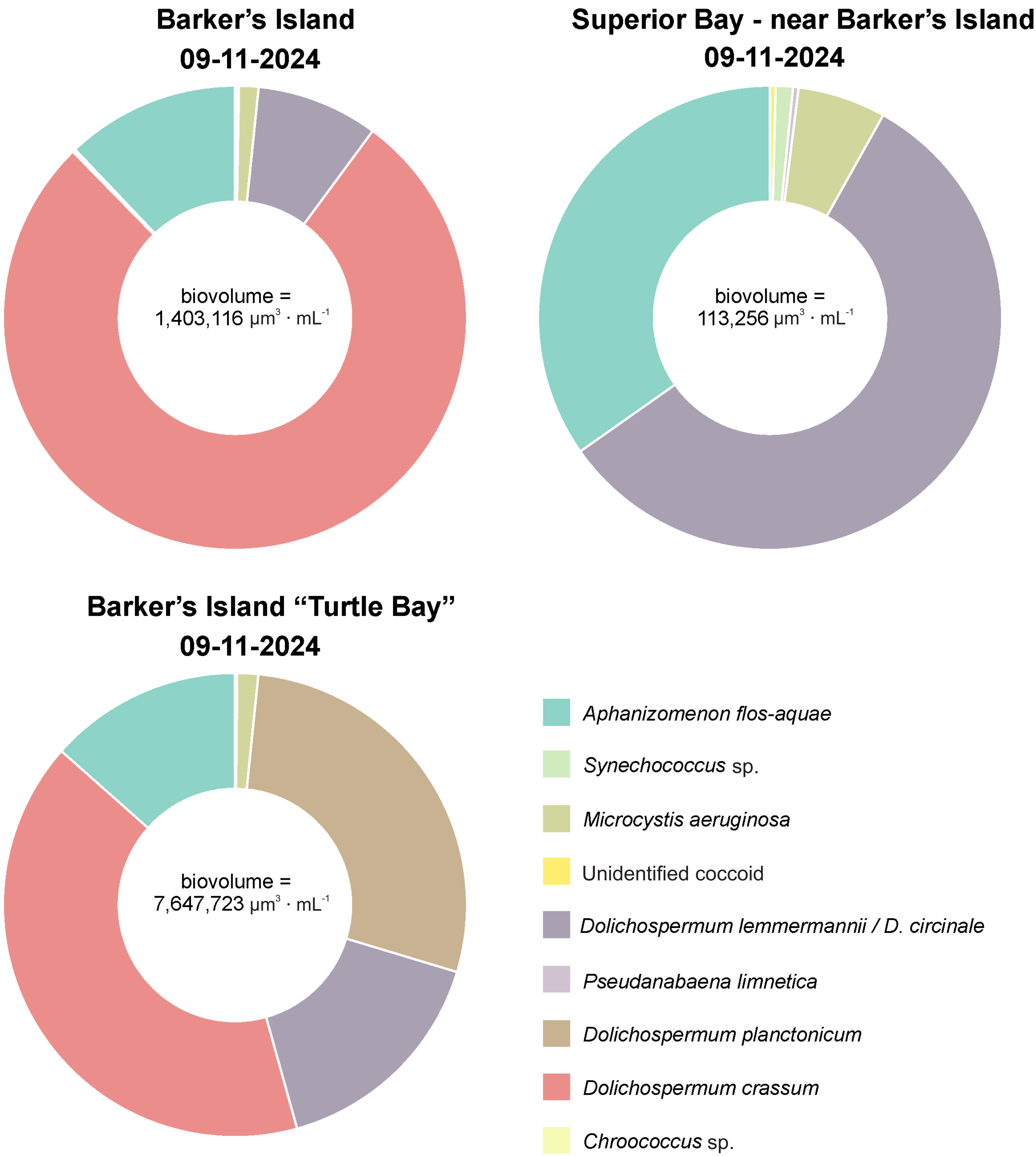
Barker's Island
09-11-2024
biovolume =
1,403,116 µm³ · mL⁻¹
Superior Bay - near Barker's Island
09-11-2024
biovolume =
113,256 µm³ · mL⁻¹
Barker's Island "Turtle Bay"
09-11-2024
biovolume =
7,647,723 µm³ · mL⁻¹
Aphanizomenon flos-aquae
Synechococcus sp.
Microcystis aeruginosa
Unidentified coccoid
Dolichospermum lemmermannii / D. circinale
Pseudanabaena limnetica
Dolichospermum planctonicum
Dolichospermum crassum
Chroococcus sp.

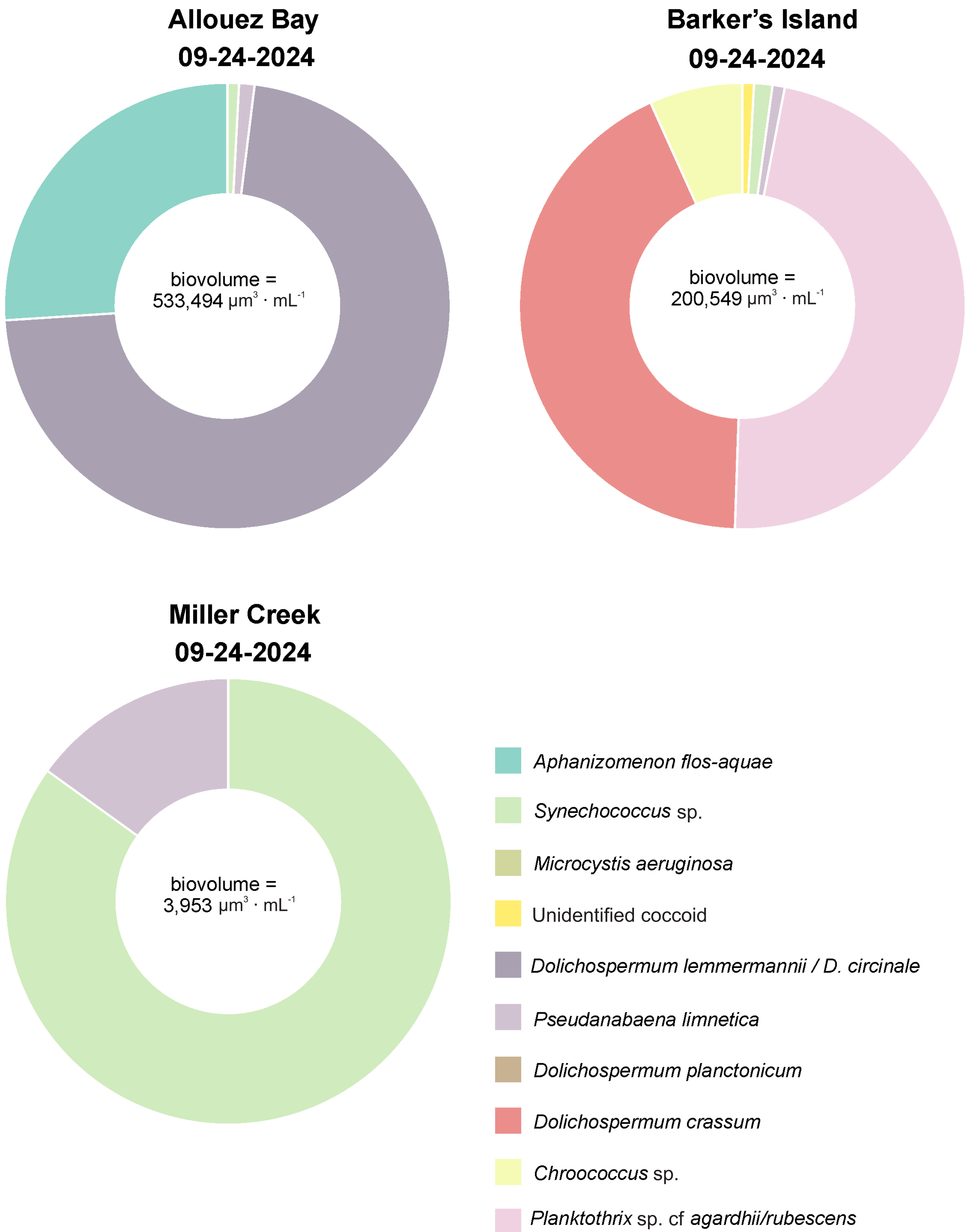

Allouez Bay
09-24-2024
biovolume =
533,494 µm³ · mL⁻¹
Barker's Island
09-24-2024
biovolume =
200,549 µm³ · mL⁻¹
Miller Creek
09-24-2024
biovolume =
3,953 µm³ · mL⁻¹
Aphanizomenon flos-aquae
Synechococcus sp.
Microcystis aeruginosa
Unidentified coccoid
Dolichospermum lemmermannii / D. circinale
Pseudanabaena limnetica
Dolichospermum planctonicum
Dolichospermum crassum
Chroococcus sp.
Planktothrix sp. cf agardhii/rubescens

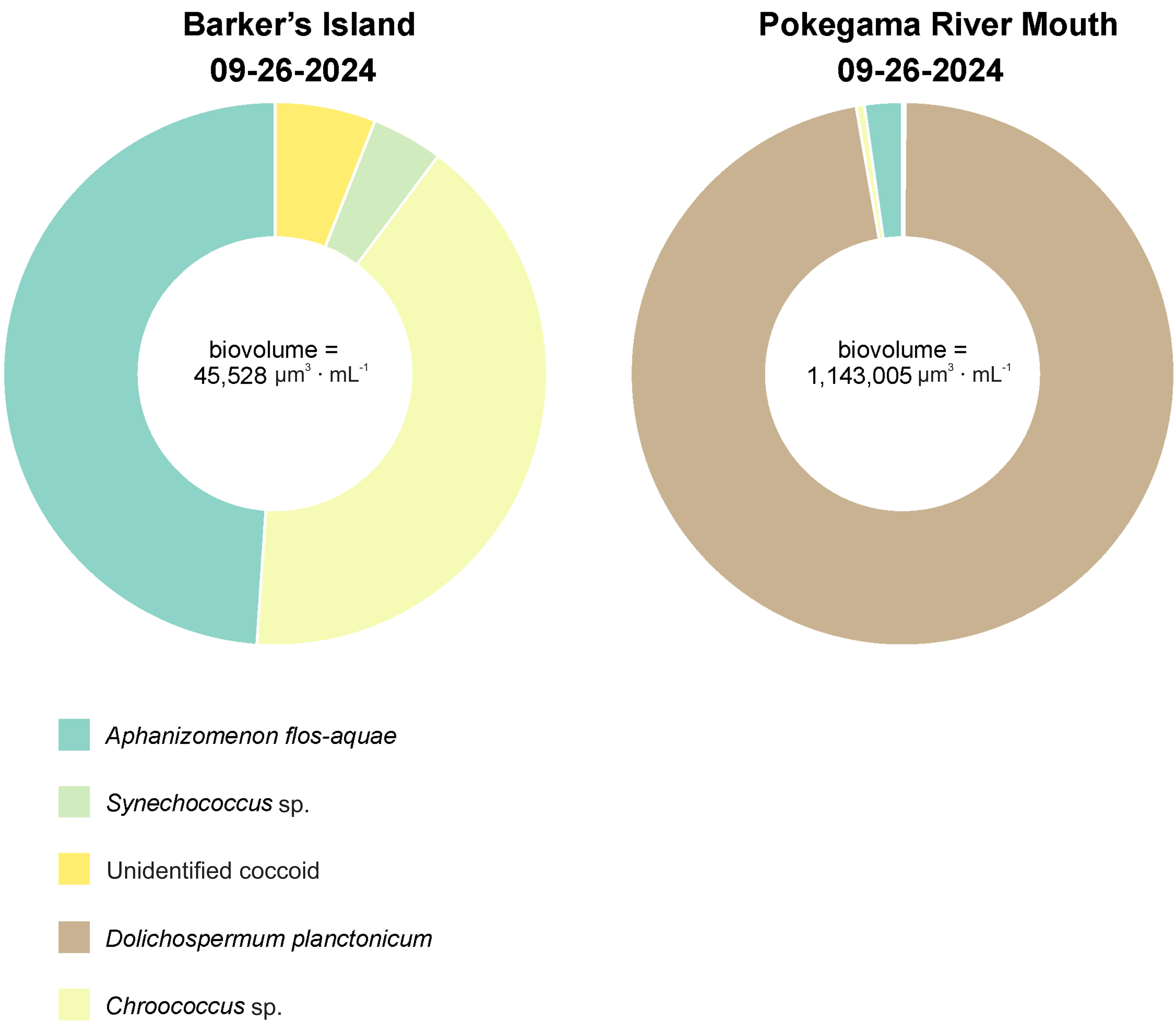

Barker's Island
09-26-2024
biovolume =
45,528 µm3 · mL-1
Pokegama River Mouth
09-26-2024
biovolume =
1,143,005 µm3 · mL-1
Aphanizomenon flos-aquae
Synechococcus sp.
Unidentified coccoid
Dolichospermum planctonicum
Chroococcus sp.
