# Supplementary material for: Optimizing a long‐term monitoring strategy to track emerging cyanobacterial blooms in a Great Lakes estuary
Source: J Phycol. 2026 Aug 7;62(4):1264–96. doi: 10.1111/jpy.70215 (PMC13485230; doi:10.1111/jpy.70215)
Supplement: Supplementary file 4 [file JPY-62-1264-s001.docx]

**Table S1:** Summary water quality data for the project sample locations, for the sample period (October 2022 - November 2024), not including winter collections. Empty cells indicate no data collected for that parameter. Water quality variable names corresponding with abbreviations are included in Table 2. Although summary data exclude winter collections, the total number of routine and bloom response sample sets collected at each site throughout the entire sample period are indicated in the first two rows.

|  |  | **North Bay (NO)** | **Mud Lake (MU)** | **Poke-**  **gama Bay (PO)** | **Kings-**  **bury Bay (KI)** | **Billings Park (BI)** | **Miller Creek (MI)** | **Barker's Island (BA)** | **Allouez Bay (AL)** |
| --- | --- | --- | --- | --- | --- | --- | --- | --- | --- |
| ***n* routine** |  | **30** | **29** | **29** | **30** | **35** | **29** | **50** | **32** |
| ***n* response** |  | **0** | **0** | **0** | **0** | **0** | **2** | **6** | **1** |
| **temp (°C)** | Min | 3.921 | 4.375 | 4.119 | 4.448 | 4.583 | 5.791 | 3.970 | 5.026 |
|  | Ave | 17.451 | 16.752 | 16.574 | 17.357 | 17.129 | 17.076 | 16.226 | 16.235 |
|  | Max | 25.356 | 25.231 | 26.784 | 25.338 | 25.569 | 24.248 | 25.742 | 24.981 |
| **DO**  **(mg • L^-1^)** | Min | 4.570 | 5.785 | 2.782 | 5.130 | 5.056 | 4.700 | 5.440 | 5.502 |
|  | Ave | 8.439 | 8.715 | 6.142 | 8.650 | 8.407 | 8.793 | 9.055 | 8.921 |
|  | Max | 12.884 | 12.528 | 11.436 | 12.058 | 11.852 | 12.025 | 12.806 | 12.087 |
| **pH** | Min | 7.005 | 7.124 | 6.992 | 7.252 | 7.004 | 7.225 | 7.234 | 7.245 |
|  | Ave | 7.715 | 7.695 | 7.320 | 7.724 | 7.678 | 7.693 | 7.779 | 7.736 |
|  | Max | 8.494 | 8.084 | 7.545 | 8.297 | 8.101 | 8.522 | 8.780 | 8.579 |
| **spcond**  **(mS • cm^-1^)** | Min | 0.097 | 0.112 | 0.117 | 0.099 | 0.100 | 0.161 | 0.142 | 0.103 |
|  | Ave | 0.216 | 0.221 | 0.241 | 0.206 | 0.200 | 0.281 | 0.198 | 0.157 |
|  | Max | 0.301 | 0.307 | 0.333 | 0.317 | 0.290 | 0.414 | 0.291 | 0.210 |
| **sal**  **(PSU)** | Min | 0.050 | 0.050 | 0.060 | 0.050 | 0.049 | 0.076 | 0.070 | 0.050 |
|  | Ave | 0.103 | 0.105 | 0.115 | 0.098 | 0.095 | 0.134 | 0.094 | 0.074 |
|  | Max | 0.140 | 0.150 | 0.160 | 0.150 | 0.140 | 0.199 | 0.140 | 0.100 |
| **Secchi (m)** | Min | 0.320 | 0.280 | 0.000 | 0.215 | 0.180 | 0.335 | 0.350 | 0.055 |
|  | Ave | 0.865 | 0.868 | 0.202 | 0.662 | 0.718 | 0.741 | 0.718 | 0.336 |
|  | Max | 1.565 | 1.805 | 0.600 | 1.200 | 1.330 | 1.085 | 1.295 | 0.765 |
| **chl *a***  **(µg • L^-1^)** | Min | 1.3 | 0.9 | 1.1 | 1.1 | 1.2 | 1.0 | 2.8 | 2.5 |
|  | Ave | 6.7 | 4.4 | 5.7 | 5.1 | 5.6 | 13.0 | 12.0 | 14.2 |
|  | Max | 16.8 | 11.9 | 20.5 | 18.2 | 17.1 | 49.2 | 34.7 | 30.5 |
| **TP (ppb)** | Min | 24.0 | 29.0 | 65.0 | 29.7 | 28.0 | 37.0 | 13.0 | 38.0 |
|  | Ave | 46.3 | 44.9 | 168.1 | 54.3 | 49.5 | 66.5 | 50.6 | 83.8 |
|  | Max | 80.0 | 100.7 | 366.6 | 119.0 | 107.2 | 112.0 | 88.0 | 188.1 |
| **SRP (ppb)** | Min | 2.9 | 1.0 | 3.8 | 4.0 | 1.8 | 3.0 | 2.9 | 1.8 |
|  | Ave | 10.5 | 10.7 | 18.0 | 10.6 | 10.3 | 18.4 | 10.4 | 7.2 |
|  | Max | 25.0 | 23.0 | 37.0 | 28.0 | 22.0 | 62.0 | 28.0 | 20.0 |
| **TN (ppb)** | Min | 284.0 | 286.0 | 371.0 | 268.0 | 270.0 | 411.0 | 328.0 | 279.0 |
|  | Ave | 762.1 | 758.1 | 1190.4 | 797.9 | 793.4 | 1217.9 | 896.3 | 755.6 |
|  | Max | 1246.0 | 1238.0 | 2424.0 | 1254.0 | 1302.0 | 1956.0 | 1304.0 | 1259.2 |
| **NH_4_ (ppb)** | Min | 3.0 | 7.0 | 4.0 | 4.0 | 3.0 | 43.0 | 9.0 | 5.0 |
|  | Ave | 27.9 | 32.6 | 42.5 | 36.1 | 31.0 | 341.4 | 68.6 | 27.5 |
|  | Max | 77.9 | 79.7 | 189.0 | 77.0 | 71.0 | 867.0 | 200.9 | 71.6 |
| **NO_2_+NO_3_ (ppb)** | Min | 0.0 | 0.0 | 0.0 | 0.0 | 0.0 | 122.0 | 58.0 | 3.0 |
|  | Ave | 46.7 | 73.8 | 71.8 | 76.6 | 79.0 | 195.9 | 243.7 | 123.8 |
|  | Max | 159.8 | 204.8 | 492.1 | 193.8 | 190.6 | 450.0 | 517.0 | 276.0 |
| **TN:TP (molar)** | Min | 7.7 | 8.2 | 3.8 | 6.2 | 7.9 | 9.5 | 8.2 | 4.4 |
|  | Ave | 16.8 | 17.1 | 7.9 | 15.5 | 16.2 | 18.7 | 19.0 | 9.7 |
|  | Max | 24.7 | 26.3 | 17.0 | 29.2 | 26.0 | 30.4 | 39.3 | 19.0 |
| **ANC (mg • L CaCO_3_ ^-1^)** | Min | 30.6 | 38.1 | 47.0 | 31.1 | 32.8 | 36.5 | 29.7 | 28.1 |
|  | Ave | 80.1 | 79.7 | 91.7 | 71.3 | 70.5 | 77.1 | 60.1 | 54.8 |
|  | Max | 114.1 | 117.3 | 124.8 | 116.8 | 108.1 | 116.6 | 92.6 | 73.5 |
| **DOC (ppm)** | Min | 9.1 | 9.1 | 2.6 | 8.9 | 8.9 | 6.4 | 2.1 | 4.1 |
|  | Ave | 16.8 | 16.4 | 19.0 | 17.2 | 16.6 | 14.6 | 12.1 | 10.3 |
|  | Max | 30.0 | 29.0 | 27.7 | 30.0 | 30.0 | 28.0 | 26.0 | 18.8 |
| **TOC (ppm)** | Min | 9.2 | 9.5 | 14.1 | 9.1 | 9.2 | 7.1 | 5.2 | 4.2 |
|  | Ave | 17.3 | 16.8 | 20.3 | 17.5 | 17.1 | 15.0 | 12.7 | 10.4 |
|  | Max | 31.0 | 30.0 | 28.3 | 30.0 | 30.0 | 29.0 | 27.0 | 19.4 |
| **CO_2_ (ppm)** | Min | 440.3 | 406.4 | 868.3 | 528.7 | 392.2 | 536.8 | 164.4 | 249.1 |
|  | Ave | 938.2 | 1178.2 | 4025.3 | 1110.2 | 1067.8 | 1279.2 | 966.9 | 845.3 |
|  | Max | 1851.6 | 1762.3 | 9998.5 | 1733.9 | 2061.6 | 2147.8 | 1863.6 | 1669.3 |
| **turbidity (FNU)** | Min | 2.6 | 1.4 | 11.5 | 4.0 | 4.1 | 4.1 | 4.1 | 15.2 |
|  | Ave | 6.5 | 7.9 | 100.4 | 10.5 | 12.2 | 7.5 | 10.7 | 43.0 |
|  | Max | 19.9 | 26.0 | 494.5 | 34.8 | 49.8 | 13.6 | 26.4 | 130.5 |
| **TSS**  **(mg • L^-1^)** | Min | 0.7 | -0.7 | 5.0 | 0.5 | 2.0 | 2.0 | 2.5 | 6.0 |
|  | Ave | 5.8 | 8.4 | 45.7 | 11.5 | 5.9 | 8.3 | 6.5 | 19.1 |
|  | Max | 20.6 | 44.5 | 483.0 | 66.0 | 16.7 | 72.5 | 11.7 | 77.3 |
| **PAR_kd_** | Min | 0.6 | 1.4 | 2.2 | 1.5 | 1.5 | 1.6 | 1.3 | 1.6 |
|  | Ave | 2.9 | 3.0 | 8.4 | 3.2 | 3.2 | 3.0 | 2.9 | 4.6 |
|  | Max | 5.1 | 5.2 | 19.2 | 6.3 | 6.1 | 6.3 | 4.9 | 12.4 |

**Table S2:** Cyanotoxin testing results from limited non-routine sampling efforts in 2023 and 2024. Bloom response samples were tested using Wisconsin DNR-provided kits and analyzed by the Wisconsin State Lab of Hygiene for total microcystins and saxitoxin. Guidance Thresholds: Drinking Water (EPA 2018): Microcystin 0.3 µg • L^-1^ (children), 1.6 µg • L^-1^ (adults), no formal threshold for Saxitoxin; Recreational Water (WHO 2003): Microcystin – 8.0 µg • L^-1^; Saxitoxin 10 - 30 µg • L^-1^. ND = non-detection.

World Health Organization. (2003). *Guidelines for safe recreational water environments: Coastal and fresh waters* (Vol. 1). World Health Organization.

| **Date** | **Location** | **Microcystin (µg • L^-1^)** | **Saxitoxin (µg • L^-1^)** | **Drinking Water Exceedance** | **Recreation Exceedance** |
| --- | --- | --- | --- | --- | --- |
| Oct 3, 2023 | Barker’s Island | 0.21 | ND | Yes – children | No |
| Oct 3, 2023 | Lake Superior / Nemadji River | ND | ND | No | No |
| Sept 21, 2023 | Barker’s Island | 1.9 | 0.026 | Yes – adults & children | No |
| Aug 21, 2024 | Barker’s Island | 1.1 | ND | Yes - adults & children | No |

**Table S3:** All (107) distinct phytoplankton taxa included in the final CCA (*R*^2^_adj_ = 0.06; *ChiSquare* = 0.36; *df* = 8; *F* = 3.14; *p* = 0.001) with ordination scores for the first 3 CCA axes and statistics explaining fit of species data to environmental data. For each taxon, *R*^2^ indicates the fit of the respective taxon to the environmental gradients, and *p* indicates the significance of the fit as determined by permutation testing (999 permutations). Of 107 taxa, 77 responded significantly (*p* < 0.05) along environmental gradients.

| **Species** | **Species Code** | **DIV** | **CCA1** | **CCA2** | **CCA3** | ***R*^2^** | ***p*** |
| --- | --- | --- | --- | --- | --- | --- | --- |
| *Synura* sp. cf *petersenii*/*uvella* | SYRUVELL | CHR | -0.982 | 0.191 | 0.071 | 0.59 | 0.001 |
| *Aphanizomenon flos-aquae* | APHFLOS | CYA | 0.816 | 0.578 | -0.241 | 0.45 | 0.001 |
| *Fragilaria crotonensis* | FRACROT | BAP | 0.779 | 0.627 | 0.543 | 0.3 | 0.001 |
| *Tetraedron* spp. | TETSP | CHL | 0.991 | 0.136 | -0.314 | 0.3 | 0.001 |
| *Chrysosphaerella longispina* | CRLLONGI | CHR | -0.883 | 0.47 | -0.092 | 0.22 | 0.001 |
| colonial Chrysophyta cf *Uroglena/Uroglenopsis* spp. | CHRURSP | CHR | -0.921 | 0.389 | -0.263 | 0.21 | 0.001 |
| *Synechococcus* sp. | SYESP | CYA | 0.977 | 0.211 | -0.097 | 0.2 | 0.001 |
| *Eudorina* sp. | EUDSP | CHL | 0.938 | 0.346 | 0.217 | 0.19 | 0.001 |
| *Diatoma vulgaris* | DIAVULG | BAP | -0.974 | 0.227 | -0.353 | 0.19 | 0.001 |
| *Merismopedia* sp. | MERSP | CYA | 0.925 | 0.379 | 0.043 | 0.19 | 0.001 |
| *Surirella* sp. | SURSP | BAP | -0.106 | -0.994 | 0.778 | 0.18 | 0.001 |
| *Pseudanabaena limnetica* | PANLIMNE | CYA | -0.997 | 0.083 | -0.071 | 0.18 | 0.001 |
| *Dinobryon cylindricum* | DINCYLIN | CHR | -0.791 | 0.612 | 0.059 | 0.17 | 0.001 |
| *Pediastrum duplex* | PEDDUPL | CHL | 0.829 | 0.559 | 0.258 | 0.16 | 0.001 |
| *Aulacoseira granulata* | AULGRAN | BAC | 0.83 | -0.557 | -0.376 | 0.15 | 0.001 |
| *Aulacoseira* sp. | AULSP | BAC | 0.784 | 0.621 | 0.111 | 0.15 | 0.001 |
| *Dolichospermum* sp. cf *circinale*/*lemmermannii* | DOLCCIRL | CYA | 0.951 | 0.308 | -0.289 | 0.14 | 0.001 |
| *Navicula* sp. | NAVSP | BAP | -0.96 | -0.279 | 0.564 | 0.13 | 0.001 |
| large centric cf *Stephanodiscus*/*Cyclotella* sp. | STPCYCSP | BAC | 0.715 | 0.699 | 0.562 | 0.13 | 0.001 |
| *Monoraphidium* sp. | MONSP | CHL | 0.97 | -0.241 | -0.078 | 0.12 | 0.001 |
| *Asterionella formosa* | ASTFORM | BAP | -0.061 | 0.998 | 0.204 | 0.12 | 0.001 |
| unidentified coccoid chrysophyte unicellular | UNICOCCH | CHR | 0.866 | 0.5 | -0.088 | 0.11 | 0.001 |
| *Rhodomonas lens* | RHOLENS | CRY | 0.831 | 0.556 | -0.348 | 0.11 | 0.001 |
| cf *Nitzchia* sp. | NITSP | BAP | -0.776 | -0.631 | 0.62 | 0.11 | 0.001 |
| *Diatoma tenuis* | DIATENU | BAP | -0.974 | 0.225 | 0.101 | 0.11 | 0.001 |
| unidentified live centric diatom (d ≤ 5) | CENTSMA | BAC | 0.354 | 0.935 | -0.033 | 0.11 | 0.001 |
| *Sphaerocystis* sp. | SPCSP | CHL | 0.925 | 0.381 | 0.073 | 0.1 | 0.001 |
| *Aulacoseira* sp. (helical/spiral) | AULSPHEL | BAC | 0.747 | 0.665 | 0.743 | 0.1 | 0.001 |
| Small chained centric | CENTSMCH | BAC | 1 | -0.026 | 0.202 | 0.1 | 0.001 |
| *Ceratium hirundinella* | CERHIRUN | PYR | 0.867 | 0.499 | 0.343 | 0.1 | 0.001 |
| *Desmodesmus* sp. | DESSP | CHL | 0.996 | 0.086 | 0.17 | 0.09 | 0.001 |
| *Chroococcus* sp. | CCCSP | CYA | 0.409 | -0.912 | 0.482 | 0.09 | 0.001 |
| *Euglena* sp. | EUGSP | EUG | 0.782 | -0.623 | -0.249 | 0.09 | 0.001 |
| *Microcystis aeruginosa* | MICAERUG | CYA | 0.722 | 0.692 | 0.445 | 0.09 | 0.001 |
| *Rhodomonas minuta* | RHOMINU | CRY | 0.971 | -0.239 | -0.045 | 0.08 | 0.001 |
| *Haptophyta* | HAPSP | CHR | 0.898 | 0.44 | -0.088 | 0.08 | 0.001 |
| *Aulacoseira ambigua* | AULAMBI | BAC | 0.066 | 0.998 | -0.094 | 0.08 | 0.001 |
| *Pandorina* sp. | PANSP | CHL | 0.761 | -0.648 | 0.028 | 0.08 | 0.001 |
| *Golenkinia radiata* | GOLRADI | CHL | 0.912 | -0.409 | 0.154 | 0.08 | 0.001 |
| *Crucigenia* sp. | CRUSP | CHL | 0.59 | -0.807 | 0.431 | 0.08 | 0.001 |
| Cyanothece sp. (round edges/ovoid) | CTHSPRO | CYA | 0.983 | 0.186 | -0.245 | 0.08 | 0.001 |
| *Pseudanabaena mucicola* | PANMUCIC | CYA | 0.999 | -0.04 | -0.574 | 0.08 | 0.001 |
| unidentified live centric diatom (d > 5) | CENTLAR | BAC | 0.951 | 0.309 | 0.027 | 0.07 | 0.001 |
| *Acanthoceras* sp. | ACANTHSP | BAC | 0.935 | 0.354 | 0.554 | 0.07 | 0.001 |
| *Dolichospermum crassum* | DOLCRASS | CYA | 0.981 | 0.196 | -0.215 | 0.07 | 0.002 |
| *Skeletonema* sp. | SKESP | BAC | 0.885 | -0.465 | 0.026 | 0.07 | 0.001 |
| *Melosira varians* | MELVARI | BAC | -0.535 | -0.845 | 0.608 | 0.07 | 0.002 |
| *Phacus* sp. | PHASP | EUG | 0.38 | -0.925 | -0.498 | 0.07 | 0.001 |
| *Oocystis parva* | OOCPARV | CHL | 0.907 | 0.421 | 0.323 | 0.07 | 0.001 |
| *Chlamydomonas* sp. | CHLSP | CHL | 0.977 | -0.214 | 0 | 0.07 | 0.001 |
| *Cosmarium* sp. | COSSP | CHL | 0.3 | -0.954 | -0.205 | 0.06 | 0.001 |
| *Raphidiopsis* sp. cf *raciborskii* | RAPCRAC | CYA | -0.981 | 0.193 | 0.17 | 0.06 | 0.001 |
| *Cryptomonas erosa* | CRYEROS | CRY | 0.836 | 0.548 | -0.011 | 0.06 | 0.001 |
| *Dolichospermum planctonicum* | DOLPLANC | CYA | 0.638 | -0.77 | -1.439 | 0.05 | 0.001 |
| *Rhizosolenia* sp. | RHISP | BAC | 0.562 | -0.827 | 0.439 | 0.05 | 0.001 |
| *Dinobryon* sp. cf. *sertularia* | DINCSERT | CHR | -0.974 | 0.225 | 0.539 | 0.05 | 0.002 |
| *Eunotia* sp. | EUNSP | BAP | -0.962 | 0.272 | -0.192 | 0.05 | 0.004 |
| cf *Nitzchia*/*Fragilaria* sp. | CNITFRA | BAP | -0.539 | -0.842 | 0.103 | 0.05 | 0.002 |
| *Dinobryon divergens* | DINDIVER | CHR | 0.893 | -0.45 | -0.201 | 0.04 | 0.004 |
| Unidentified cf Euglenophyta/Cryptophyta w/"tuft" | UNITUFT | CRY | 0.637 | -0.771 | -0.253 | 0.04 | 0.006 |
| *Characium* sp. | CHASP | CHL | -0.191 | -0.982 | 0.328 | 0.04 | 0.007 |
| *Coelastrum* sp. | COESP | CHL | 0.999 | -0.054 | -0.001 | 0.04 | 0.003 |
| *Epipyxis* sp. | EPXSP | CHR | 0.065 | 0.998 | 0.002 | 0.04 | 0.005 |
| *Cryptomonas reflexa* | CRYREFLEX | CRY | 0.235 | -0.972 | 0.065 | 0.04 | 0.005 |
| *Peridinium* sp. | PERSP | PYR | 0.881 | -0.472 | 0.132 | 0.04 | 0.008 |
| *Anabaena* sp. | ANBSP | CYA | 0.5 | -0.866 | -0.849 | 0.04 | 0.011 |
| *Aulacoseira* sp. auxospore | AULSPAUX | BAC | -0.683 | 0.731 | -0.375 | 0.03 | 0.019 |
| *Gymnodinium* sp. | GYMSP | PYR | 0.934 | 0.357 | -0.135 | 0.03 | 0.016 |
| *Scenedesmus* sp. | SCESP | CHL | 0.356 | -0.934 | -0.054 | 0.03 | 0.032 |
| *Gomphonema* sp. | GOMSP | BAP | -0.872 | -0.49 | 0.514 | 0.03 | 0.023 |
| *Carteria* sp. | CARSP | CHL | 0.981 | -0.192 | 0.082 | 0.03 | 0.032 |
| Unidentified live pennate diatom | UNIBAP | BAP | 0.689 | 0.725 | 0.07 | 0.03 | 0.038 |
| *Schroederia* sp. | SCHRSP | CHL | 0.999 | -0.036 | 0.288 | 0.03 | 0.032 |
| cf *Dictyosphaerium* sp. | UNISTALK | CHL | 0.997 | -0.078 | 0.003 | 0.03 | 0.031 |
| unidentified coccoid Chlorophyta | UNICOCCL | CHL | -0.458 | -0.889 | 0.026 | 0.03 | 0.041 |
| *Kirchneriella* sp. | KIRSP | CHL | 0.827 | -0.563 | 0.194 | 0.03 | 0.032 |
| *Ankistrodesmus* sp. | ANKSP | CHL | 0.974 | -0.225 | 0.3 | 0.02 | 0.045 |
| *Dinobryon bavaricum* | DINBAVAR | CHR | 0.766 | -0.643 | -0.186 | 0.02 | 0.064 |
| *Pseudanabaena minima* | PANMINIM | CYA | -0.983 | -0.182 | -0.75 | 0.02 | 0.076 |
| *Kephyrion* sp. | KEPSP | CHR | -0.271 | 0.963 | -0.733 | 0.02 | 0.067 |
| *Chlorogonium* sp. cf *elongatum* | CHGCELON | CHL | 0.987 | 0.16 | -0.394 | 0.02 | 0.062 |
| *Chrysosphaera* sp. | CRHSP | CHR | 0.865 | 0.502 | 0.055 | 0.02 | 0.091 |
| *Treubaria* sp. | TRESP | CHL | 0.802 | -0.597 | -0.284 | 0.02 | 0.112 |
| *Fragilaria capucina* | FRACAPU | BAP | -0.721 | 0.693 | 0.469 | 0.02 | 0.107 |
| *Gloeocystis* sp. | GLOSP | CYA | 0.993 | -0.12 | -0.248 | 0.02 | 0.124 |
| *Ctenophora pulchella* | CTEPULC | BAP | -0.682 | -0.731 | -0.585 | 0.02 | 0.13 |
| *Micractinium pusillum* | MCTPUSI | CHL | 0.816 | 0.578 | 0.549 | 0.02 | 0.148 |
| *Planktothrix* sp. cf *agardhii*/*rubescens* | PLXSP | CYA | -0.699 | -0.715 | 0.104 | 0.02 | 0.135 |
| *Cocconeis* sp. | COCSP | BAP | 0.974 | 0.229 | -0.223 | 0.01 | 0.222 |
| *Bitrichia* sp. | BITSP | CHR | -0.983 | -0.184 | -0.101 | 0.01 | 0.231 |
| *Mallomonas* sp. | MALSP | CHR | 0.493 | -0.87 | 0.27 | 0.01 | 0.244 |
| *Glenodinium* sp. | GLESP | PYR | 0.415 | 0.91 | 0.124 | 0.01 | 0.306 |
| *Snowella* sp. | SNOSP | CYA | 0.873 | 0.487 | -0.03 | 0.01 | 0.277 |
| *Trachelomonas* sp. | TRASP | EUG | 0.189 | -0.982 | 0.717 | 0.01 | 0.352 |
| *Chrysamoeba* sp. | CRYSP | CHR | -0.951 | -0.308 | 0.014 | 0 | 0.561 |
| *Amphora* sp. | AMASP | BAP | -0.537 | 0.844 | 0.062 | 0 | 0.633 |
| *Synechocystis* sp. | SYCSP | CYA | -0.078 | -0.997 | -0.225 | 0 | 0.651 |
| *Entomoneis* sp. | ENTSP | BAP | -0.231 | 0.973 | 0.255 | 0 | 0.661 |
| *Chrysolykos* sp. | CRKSP | CHR | 0.746 | 0.666 | -0.376 | 0 | 0.66 |
| *Synura* sp. cf *adamsii* | SYRCADAM | CHR | 1 | 0.019 | -0.189 | 0 | 0.736 |
| *Aulacoseira* sp. cf *islandica* | AULCFISL | BAC | -0.9 | 0.436 | 0.231 | 0 | 0.79 |
| *Dinobryon* sp. | DINSP | CHR | -0.642 | -0.767 | 0.228 | 0 | 0.759 |
| *Quadrigula* sp. | QUDSP | CHL | 0.85 | -0.526 | -0.204 | 0 | 0.781 |
| *Closteriopsis* sp. | CHSSP | CHL | 0.896 | 0.444 | 0.167 | 0 | 0.819 |
| *Pedinella* sp. | PELSP | CHR | -0.119 | -0.993 | 0.041 | 0 | 0.919 |
| *Pseudokephyrion millerense* | PSKMILLE | CHR | -0.823 | -0.568 | 0.253 | 0 | 0.92 |
| *Diatoma* spp. | DIASP | BAP | -0.768 | -0.64 | -0.7 | 0 | 0.947 |
